# Amygdala astrocyte primary cilium mechanisms contribute to stress behaviours

**DOI:** 10.1038/s41586-026-10874-0

**Published:** 2026-08-05

**Authors:** Sara G. Pelaz, Katsukuni Mitsui, Natalia Kolosowska, Haley Fritch, Chiranjivi Neupane, Vanessa H. Casha, Marta Alonso-Gardón, Vijaya Pandey, Lizheng Wang, Riki Kawaguchi, James A. Wohlschlegel, Jiami Guo, Steven A. McCarroll, Sabina Berretta, Baljit S. Khakh

**Affiliations:** 1 Department of Physiology, David Geffen School of Medicine, University of California Los Angeles, Los Angeles, CA USA; 2 Research Center of Neurology, ONO Pharmaceutical, Osaka, Japan; 3 McLean Hospital, Belmont, MA USA; 4 Department of Psychiatry, Program in Neuroscience, Harvard Medical School, Boston, MA USA; 5 Broad Institute of MIT and Harvard, Cambridge, MA USA; 6 Department of Biological Chemistry, David Geffen School of Medicine, University of California Los Angeles, Los Angeles, CA USA; 7 Department of Cell Biology and Anatomy, Alberta Children’s Hospital Research Institute, Hotchkiss Brain Institute, Cumming School of Medicine, University of Calgary, Calgary, Alberta Canada; 8 Program in Neurogenetics, Department of Neurology, David Geffen School of Medicine, University of California Los Angeles, Los Angeles, CA USA; 9 Center for Neurobehavioral Genetics, Semel Institute for Neuroscience and Human Behavior, University of California Los Angeles, Los Angeles, CA USA; 10 Howard Hughes Medical Institute and Harvard Medical School Department of Genetics, Boston, MA USA; 11 Department of Neurobiology, David Geffen School of Medicine, University of California Los Angeles, Los Angeles, CA USA; 12 UK Dementia Research Institute, Cardiff University, Cardiff, UK

**Keywords:** Astrocyte, Depression, Cellular neuroscience, Depression, Molecular neuroscience

## Abstract

Understanding how adverse life events trigger stress-related behavioural changes remains an unresolved challenge. The amygdala is integral to emotion and stress responses^1^ and comprises astrocytes, neurons and other cells. Here we show that amygdala astrocytes contribute to stress-related behaviours through signalling mechanisms related to their primary cilia^2^. Amygdala astrocytes are altered during stress at the protein and gene expression level, display reduced expression of molecules related to primary cilia^3,4^ and have morphologically short primary cilia^5,6^. G protein-coupled receptors (GPCRs) are central to astrocyte^7^ and primary cilia^2,8,9^ function. Therefore, we speculated that GPCR signalling activation might be beneficial in stress-related behavioural disorders. We identified amygdala astrocyte GPCRs as regulators of responses following stress. Chemogenetics and targeting of native sphingosine-1-phosphate receptor 1 (S1PR1) GPCRs led to the restoration of astrocyte primary cilia length, corrected molecular alterations and improved stress-related behaviours. Cilium-related genes were abundantly expressed in human amygdala astrocytes, with many displaying disrupted expression in stress-related brain disorders. S1PR1 was also highly expressed in amygdala astrocytes from human tissue. Selective genetic disruption of amygdala astrocyte primary cilia in mice altered some stress-related behaviours and gene expression of astrocytes and parenchymal cells. These data confirm that astrocytic cilia have important roles in this brain nucleus. In summary, amygdala astrocytes and their primary cilia are disrupted during stress, and their restoration is accompanied by stress-related molecular and behavioural improvements. Astrocyte primary cilia-related mechanisms may therefore provide new treatment strategies for stress-related and other brain disorders.

## Abstract (web summary)

Amygdala astrocyte primary cilia are disrupted during stress, and their restoration leads to improvements in stress-related behaviour in mice.

## Main

Unravelling the mechanisms that link adverse life events to stress-related behavioural changes and disorders such as depression and anxiety represent an unmet goal of neuroscience. Important advancements have identified several brain regions associated with stress-related disturbances of emotion, reward and cognitive function, including the basolateral amygdala (BLA)^1,10–12^. The BLA has a crucial role in associative learning, particularly for fear and reward, by encoding emotional valence of stimuli and by influencing motivated behaviours. The BLA is activated by stress and is altered in conditions of pathological stress^1,10–12^. However, the molecular and cellular basis of stress-related changes in the amygdala are not well understood. Moreover, because many past studies have focused on neurons, the contributions of non-neuronal cells such as astrocytes to stress-related behaviours remain incompletely explored^13^.

Astrocytes, a type of glial cell, are crucial components of neural circuits and have essential roles in physiology and disease^14^. Recent studies have shown that astrocytes are altered in psychiatric disorders and in animal models of psychiatric disorders^15–25^. These findings underscore the importance of exploring whether astrocytes contribute to stress-related pathophysiological processes in the central nervous system (CNS). Amygdala astrocytes are altered in rodent models of stress^26^, and both astrocytes and neurons contribute to amygdala function^25,27–31^. Stress-related behavioural disorders represent a major unmet clinical need^32^, but the possibility that amygdala astrocytes can contribute to and be used for new therapeutic strategies remains largely unexplored.

The primary cilium is an antenna-like membrane protrusion adorning cells that integrates extracellular cues and serves essential roles in signalling^2,8,9,33^. Relatively little is known about astrocyte primary cilia^5,34,35^; however, their deletion causes molecular and developmental changes^5^. In this study, we use various mouse models of stress and multiple approaches to elucidate how amygdala astrocytes change molecularly and cellularly. We then identify astrocyte-related signalling mechanisms that may enhance positive valence during aberrant stress-related behaviours. Unexpectedly, our evaluations reveal astrocyte primary cilium mechanisms associated with stress-related behavioural disturbances. Moreover, S1PR1 GPCRs, which are shared between humans and mice, are potential targets to restore primary cilia and astrocytic contributions. Complementary evidence from mice and humans indicate that amygdala astrocyte primary cilia are disrupted during stress and that their restoration is associated with stress-related improvements in mice. We therefore identify previously unknown mechanisms of amygdala astrocytes that can potentially be used to treat stress-related disorders.

## Stress-related behavioural phenotypes

**Fig. 1:**
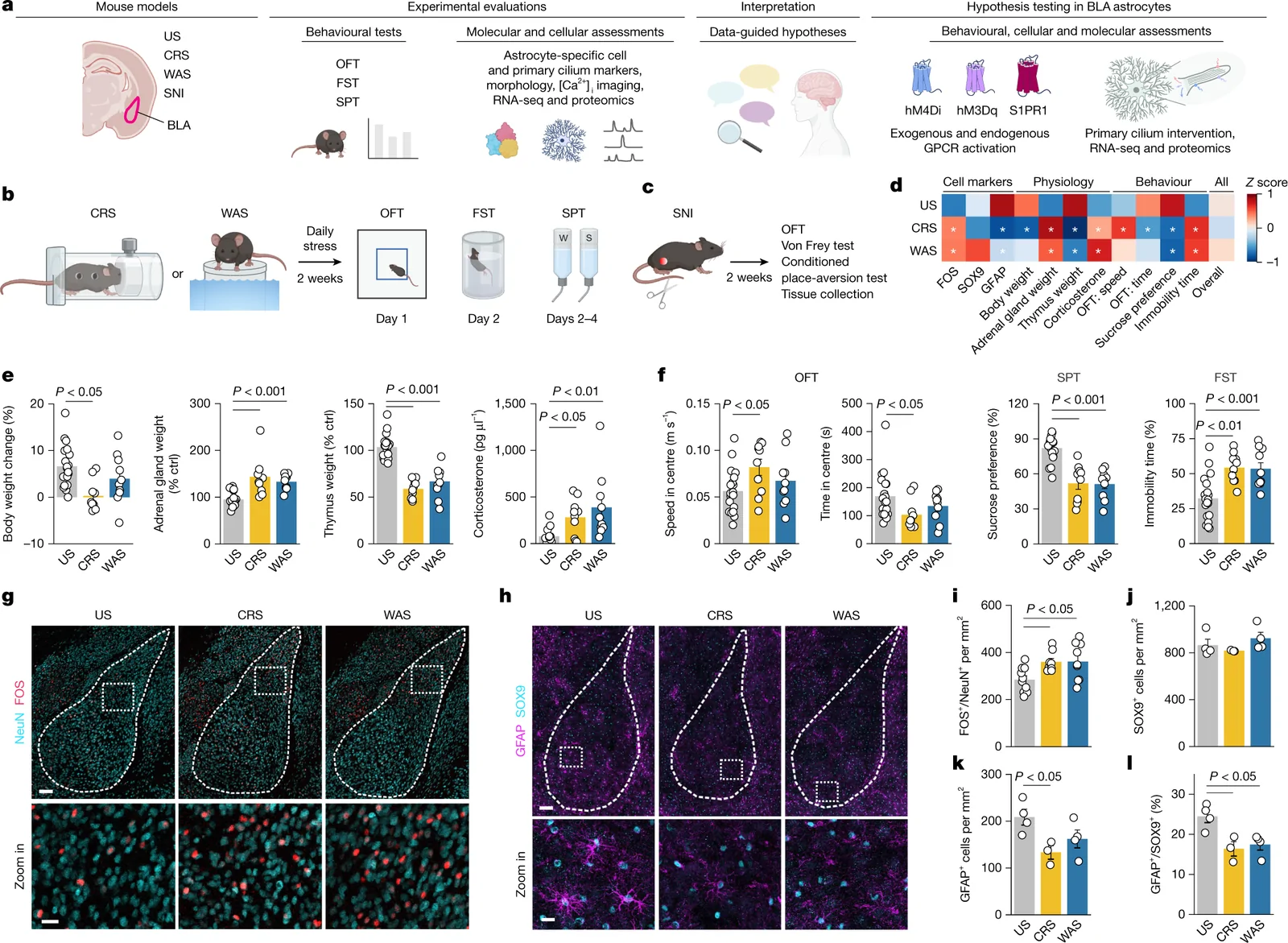
Stress-related behavioural phenotypes. **a**, Summary of the study to identify amygdala astrocyte mechanisms of relevance to stress-related behaviours. **b**,**c**, CRS and WAS (**b**) and SNI (**c**) stress models used, and subsequent readouts. **d**, Heatmap of cellular, physiological and behavioural readouts after stress. CRS induced stronger phenotypes than WAS. **e**, Physiological metrics. *n* (mice) = 18–20 (US), 10 (CRS) and 10 (WAS). *P* values versus US: body weight CRS *P* = 1.48 × 10^−3^; adrenal gland weight CRS *P* = 3.03 × 10^−4^ and WAS *P* = 3.64 × 10^−4^; thymus weight CRS and WAS *P* < 1 × 10^−4^; corticosterone CRS *P* = 2.30 × 10^−2^ and WAS *P* = 2.84 × 10^−3^. **f**, Behavioural metrics. *n* (mice) = 20 US, 10 CRS and 10 WAS. *P* values versus US: OFT (speed) CRS *P* = 3.63 × 10^−2^; OFT (time) CRS *P* = 2.84 × 10^−2^; SPT CRS and WAS *P* < 1 × 10^−4^; FST CRS *P* = 6.62 × 10^−4^ and WAS *P* = 1.06 × 10^−3^. **g**, Representative images of amygdala tissue sections showing NeuN (cyan) and FOS (red) immunostaining. Scale bars, 100 µm (top), 25 µm (bottom). **h**, Representative images of amygdala tissue sections showing GFAP (magenta) and SOX9 (cyan) immunostaining. Scale bars, 100 µm (top), 25 µm (bottom). **i**, Quantification of FOS-positive neurons in US (grey), CRS (yellow) and WAS (blue) mice. **j**–**l**, Quantification of SOX9-positive (**j**) cells, GFAP-positive cells (**k**) and the proportion of SOX9-positive cells that expressed GFAP (**l**). Bars represent the mean and error bars represent the s.e.m. Statistical tests and values are reported in Supplementary Table 1. Icons in **a**–**c** created in BioRender; Gutierrez Pelaz, S. https://biorender.com/evoeh1p (2026). Source data

We studied amygdala astrocytes in control unstressed (US) mice and in mice subjected to chronic restraint stress (CRS), water avoidance stress (WAS) or spared nerve injury (SNI), which model chronic, psychological and pain-mediated stress, respectively^36–42^ (Fig. 1a). After stress induction, we performed stress-related physiological and behavioural evaluations alongside comprehensive molecular and cellular profiling of astrocytes. In contrast to previous neuron-centric studies, our experimental design deliberately prioritized the identification of astrocyte mechanisms. We note that synaptic and neuronal circuit mechanisms of stress will require dedicated circuit-mapping approaches for full definition. However, the astrocyte-focused findings we report here provide molecular entry points to meaningfully assess the contributions of astrocytes to these circuits. Accordingly, on the basis of our findings, we formulated and tested hypotheses centred on astrocytes and their primary cilia (Fig. 1a).

**Extended Data Fig. 1:**
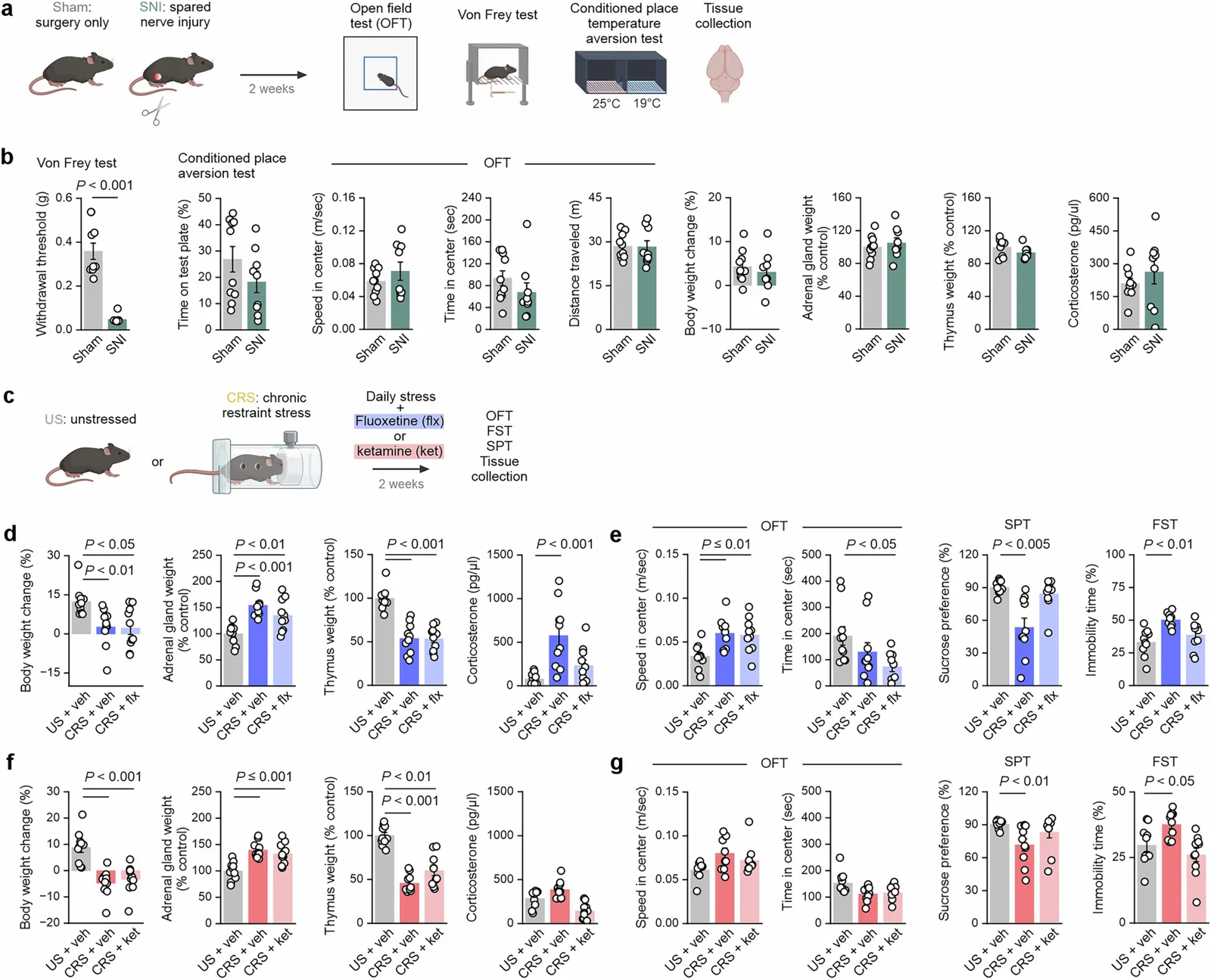
Additional behavioral data. **a**, **b**, Cartoon and data for readouts after SNI (n (mice) = 9 – 10 per group; *P* values v sham: Von Frey *P* = 2.31 × 10^−4^). **c**, Cartoon illustrating the experimental strategy for fluoxetine or ketamine treatment in CRS mice. Fluoxetine (10 mg/kg) was injected daily during the stress and the four measurement days, and ketamine (30 mg/kg) was injected daily for the four measurement days. **d**, Physiological metrics. Changes in body weight, adrenal gland weight, thymus weight and corticosterone levels (numbers of mice; 10 US + vehicle, 10 CRS + vehicle, 10 CRS + fluoxetine; *P* values v US + veh: body weight CRS + veh *P* = 7.52 × 10^−3^, CRS + flx *P* = 8.55 × 10^−3^, adrenal glands CRS + veh *P* < 1 × 10^−4^, CRS + flx *P* = 8.81 × 10^−3^, thymus CRS + veh, CRS + flx *P* < 1 × 10^−4^, corticosterone CRS + veh *P* = 3.95 × 10^−4^). **e**, Behavioral metrics. Speed and time in the center in OFT recordings, SPTs, and immobility time in FST (numbers of mice; 10 US + vehicle, 10 CRS + vehicle, 10 CRS + fluoxetine; *P* values v US + veh: OFT speed CRS + veh *P* = 5.47 × 10^−3^, CRS + flx *P* = 1.03 × 10^−2^, OFT time CRS + flx *P* = 2.59 × 10^−2^, SPT CRS + veh *P* = 6.26 × 10^−4^, FST CRS + veh *P* = 1.17 × 10^−3^). **f-g**, As in d-e, but for treatments with ketamine (n (mice) = 10 US + vehicle, 10 CRS + vehicle, 10 CRS + ketamine; *P* values v US + veh: body weight CRS + veh *P* < 1 × 10^−4^, CRS + ket *P* = 1.04 × 10^−4^, adrenal glands CRS + veh *P* < 1 × 10^−4^, CRS + ket *P* = 1.06 × 10^−3^, thymus CRS + veh *P* < 1 × 10^−4^, CRS + ket *P* = 8.17 × 10^−3^, SPT CRS + veh *P* = 7.52 × 10^−3^, FST CRS + veh *P* = 4.54 × 10^−2^). This figure should be used in conjunction with main Fig. 1 and the related text. Abbreviations: flx, fluoxetine; ket, ketamine. Bars represent the mean and error bars represent the s.e.m. Statistical tests and values are reported in Supplementary Table 1. Icons in **a** and **c** created in BioRender; Gutierrez Pelaz, S. https://biorender.com/evoeh1p (2026). Source data

We used CRS, WAS and SNI as stressors to evaluate subsequent physiological and behavioural changes in comparison to control US mice (Fig. 1b,c). For CRS and WAS, the mice underwent 6 h of restraint or 2 h of water avoidance on a small platform, respectively, for 14 days. The mice then performed open-field tests (OFTs), forced-swim tests (FSTs) and sucrose-preference tests (SPTs), which capture behaviours related to anxiety, depression and anhedonia, respectively (Fig. 1b). After the behavioural tests, brains were collected for analyses. For models of SNI stress, the common peroneal and tibial sciatic nerves were ligated and transected, whereas the sural nerve was spared (Fig. 1c). Mice with SNI displayed significantly increased sensitivity in the Von Frey test, which indicated hyperalgesia in the innervated paw (Extended Data Fig. 1a,b; *n* = 9–10 mice per group). However, this did not progress to the development of stress-related physiological changes or behaviours (Extended Data Fig. 1a,b; *n* = 9–10 mice per group).

Marked phenotypes were observed following CRS and WAS (Fig. 1d and Supplementary Table 1), as evidenced by decreased body weight, decreased thymus weight, increased adrenal gland weight and elevated corticosterone levels (Fig. 1e; *n* = 10–20 mice per group). These results reflect established changes^43–47^ caused by successful stress induction in the mice. Furthermore, CRS mice displayed significant changes in OFTs, and both CRS and WAS mice displayed significant behavioural alterations in SPTs and FSTs, which indicated stress-related behaviours related to anxiety, depression and anhedonia, respectively (Fig. 1d,f; *n* = 10–20 mice per group). Although CRS and WAS mice exhibited similar physiological readouts of stress, at the behavioural level, CRS mice were most robust (Fig. 1d–f). To broadly assess whether CRS and WAS lead to alterations in astrocytes and neurons in the amygdala, we performed immunohistochemistry (IHC) to evaluate protein expression levels of the immediate early gene *Fos*. FOS expression was increased in amygdala neurons (Fig. 1g,i) but not in astrocytes, as 94 ± 2, 99 ± 0.4 and 97 ± 1% of the FOS-positive cells were also NeuN-positive in US, CRS and WAS groups, respectively (*n* = 10, 8 and 9 mice, respectively, per group). GFAP levels generally decrease in amygdala astrocytes in models of early life stress^28^, depression and in postmortem human tissue from patients with major depressive disorder (MDD)^48^. Accordingly, the number of GFAP-positive astrocytes after CRS or WAS was decreased (Fig. 1h,k), with no changes in the number of SOX9-positive astrocytes^26^ (Fig. 1j–l). GFAP levels increase in other disease settings^49^.

Stress-related behaviours such as depression are often treated with antidepressants^50^. Therefore, we used two such drugs to determine which of the above-reported metrics are altered^50–52^: 30 mg kg^–1^ ketamine and 10 mg kg^–1^ fluoxetine (*n* = 10 mice per group, and *n *= 9–10 vehicle controls). We used the CRS model because it produced the most reliable behavioural changes (Fig. 1). Although both fluoxetine and ketamine improved some CRS-induced behaviours, ketamine was more effective (Extended Data Fig. 1c–g). However, the antidepressants were incompletely effective; complete rescue of the stress-related behavioural changes was not expected and did not occur.

## Stress-induced astrocyte molecular changes

**Fig. 2:**
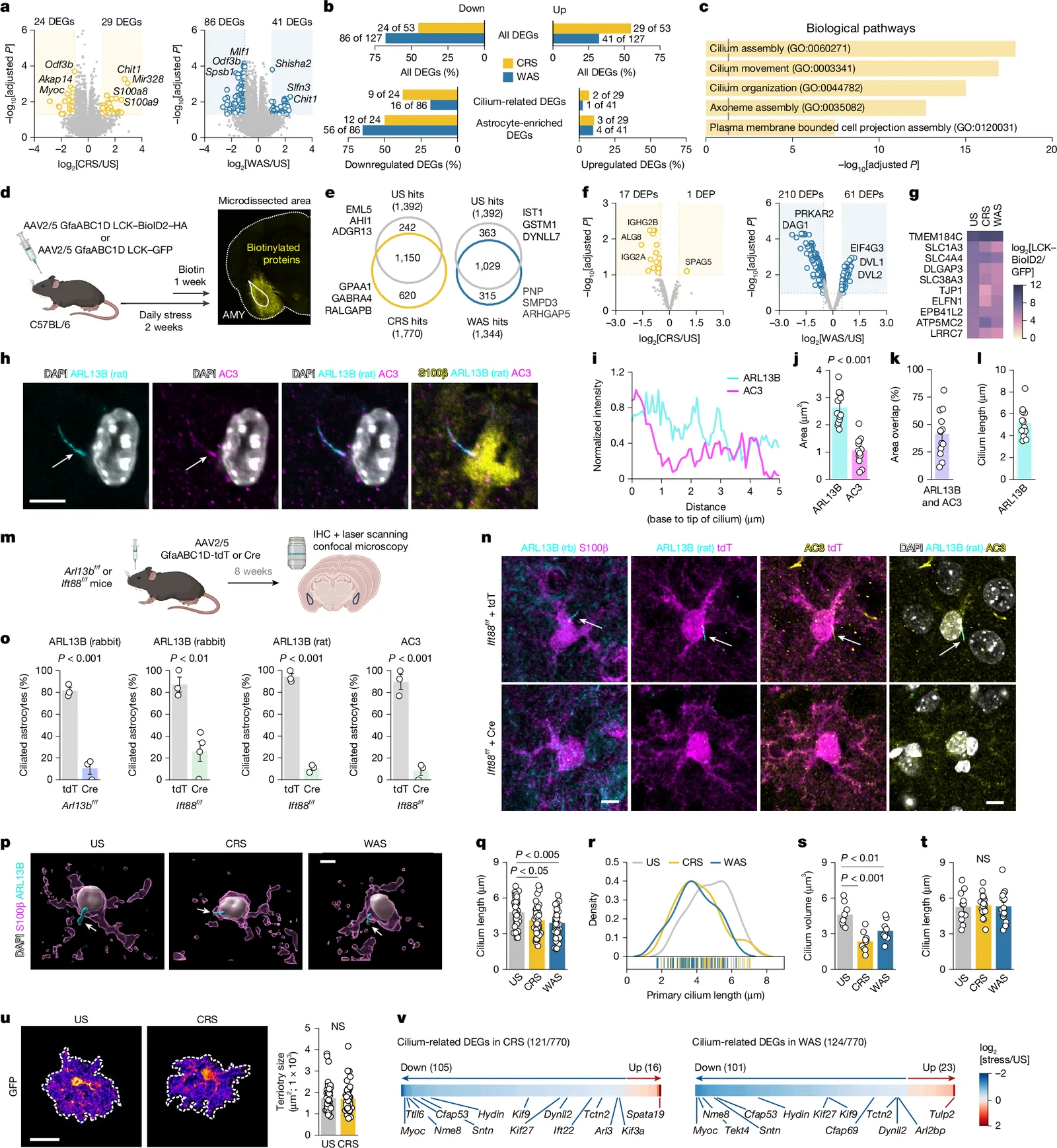
Molecular and cellular changes in amygdala astrocytes after CRS and WAS. **a**, Volcano plots for RiboTag RNA-seq data from amygdala astrocytes after CRS (left) or WAS (right) in *Aldh1l1*^*cre/ERT2*^;RiboTag mice (absolute log_2_[stress/US] > 1, adjusted *P* < 0.05). **b**, Proportion of DEGs, astrocyte-enriched DEGs and cilium-related DEGs in each stress model. In **a** and **b**, DEGs were obtained using limma-voom. Astrocyte-enriched DEGs: absolute log_2_[US IP/input] > 1 and adjusted *P* < 0.05. Cilium-related DEGs: DEGs filtered on the basis of UniProt and GeneCards (shown in Extended Data Fig. 2e). **c**, Astrocyte downregulated DEGs (adjusted *P* < 0.05) in CRS mice are enriched in cilium-related biological processes (obtained using Enrichr, Fisher’s exact test followed by Benjamini–Hochberg correction). **d**, Left, strategy for astrocyte near-membrane proteomics. Right, image showing biotinylation and the area microdissected as the amygdala (AMY). **e**, Venn diagram of shared and unique hits in each group. **f**, Volcano plots of proteomic data for CRS mice (left) and WAS mice (right) compared with US mice. DEPs were obtained using limma. **g**, Top ten shared proteomic hits between all groups. **h**, Representative example of high-resolution confocal imaging of a primary cilium (indicated by arrows) in a BLA astrocyte stained with ARL13B and AC3 (for **h**–**l**, *n* = 13 astrocytes from 4 mice). Scale bar, 5 µm. **i**, Line analysis of ARL13B and AC3 in the image from **h**. **j**, Area stained by ARL13B and AC3 (*P* < 1 × 10^−4^). **k**, Area overlap of ARL13B and AC3 staining. **l**, Primary cilium length based on ARL13B staining. **m**, Schematic of genetic cilium interventions in BLA astrocytes. **n**, Representative images of BLA astrocytes from *Ift88*^*f/f*^ mice that received tdTomato (tdT) or Cre AAVs. Note that astrocytes in the Cre group no longer possess an ARL13B^+^ or AC3^+^ primary cilium (indicated by arrows). Scale bars, 5 µm. **o**, Proportion of BLA astrocytes with an ARL13B or AC3 stained primary cilium in *Arl13b*^*f/f*^ and *Ift88*^*f/f*^ mice. *n* = 3–4 mice per group; *Arl13b*^*f/f*^ + anti-ARL13B (rabbit) *P* = 3.05 × 10^−4^; *Ift88*^f/f^ + anti-ARL13B (rabbit) *P* = 4.12 × 10^−3^; *Ift88*^*f/f*^ + anti-ARL13B (rat) *P* < 1 × 10^−4^; *Ift88*^*f/f*^ + anti-AC3 *P* = 5.06 × 10^−4^. **p**, Representative 3D-rendered images of immunostained astrocytes (S100β, magenta) showing the cell nucleus (DAPI, white) and the cilium (ARL13B, cyan; indicated by arrows). Scale bars, 4 µm. **q**, Astrocyte cilium length (number of astrocytes = 40 (US), 45 (CRS) and 45 (WAS); *n* = 4 mice per group). **r**, Distribution plot of the data shown in **q**. **s**, Astrocyte cilium volume measured in 3D-rendered representations based on IHC images (number of astrocytes = 8 (US), 10 (CRS) and 9 (WAS); *n* = 4 mice per group; *P* values versus US: CRS *P* < 1 × 10^−4^; WAS *P* = 8.36 × 10^−3^). **t**, Neuronal cilium length measured from ARL13B-stained IHC images (number of neurons = 11 (US), 21 (CRS) and 15 (WAS); *n* = 4 mice per group). NS, not significant. **u**, Representative images (left) of outlined astrocyte territories from US and CRS mice injected with AAV2/5 GfaABC1D–GFP and quantification (right) of territory size (number of astrocytes = 46 (US) and 53 (CRS); *n* = 4 mice per group). Scale bar, 20 µm. **v**, Cilium-related DEGs from BLA astrocyte RiboTag RNA-seq following stress. Bars represent the mean and error bars represent the s.e.m. Statistical tests and values are reported in Supplementary Table 1. Icons in **d** and** m** created in BioRender; Gutierrez Pelaz, S. https://biorender.com/evoeh1p (2026). Source data

**Extended Data Fig. 2:**
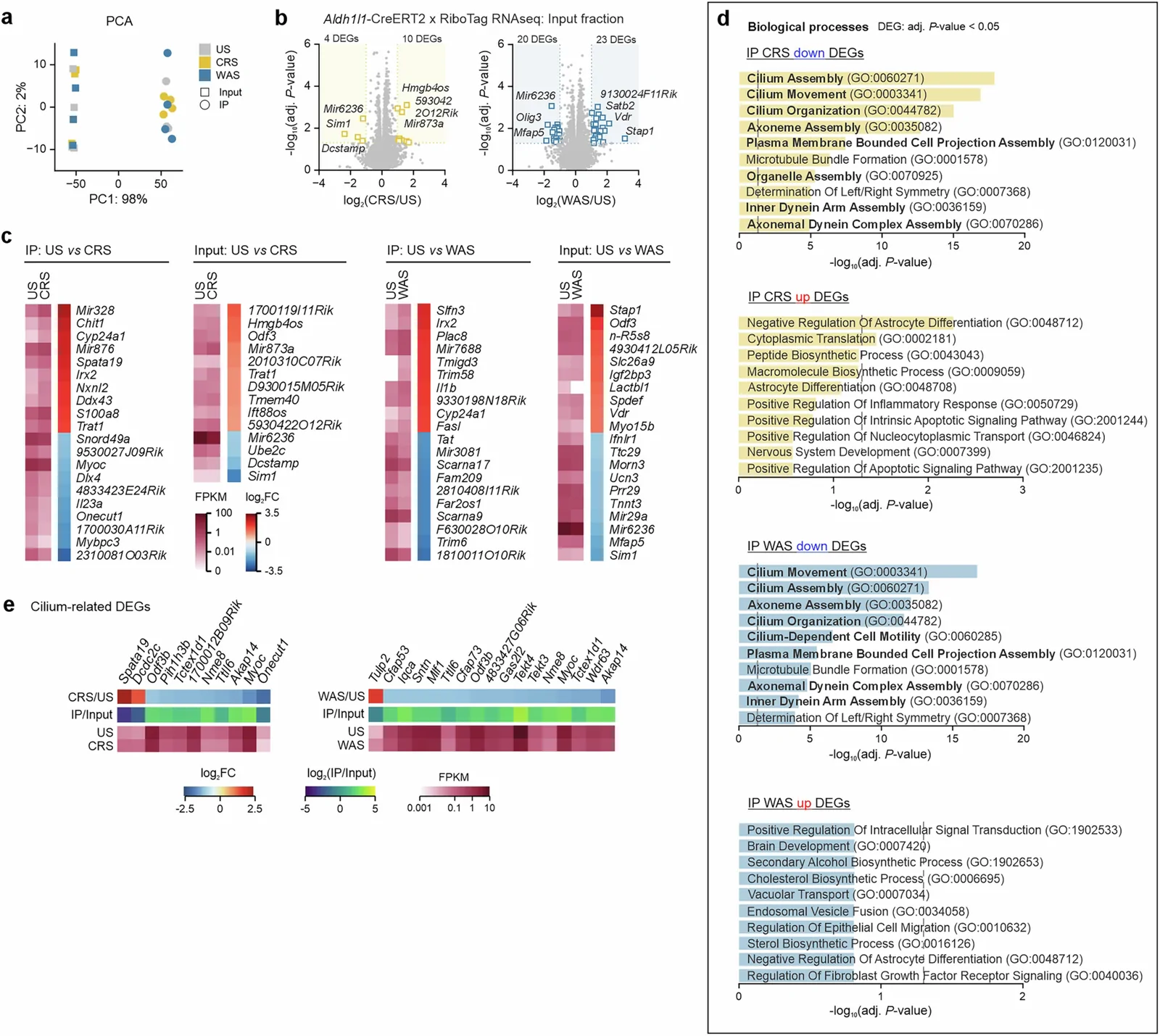
Additional RNAseq analyses of stress. **a**, PCA plot of US, CRS and WAS RNAseq data. **b**, Volcano plots for RNAseq data from amygdala input fractions after CRS or WAS. DEGs were obtained with limma-voom. **c**, Top up and down-regulated genes in IP and input fractions in CRS and WAS. **d**, Biological processes (obtained with Enrichr, Fisher’s exact test followed by Benjamini-Hochberg) from CRS and WAS IP fractions when DEGs were defined as those with adj. *P*-value < 0.05 (no fold change filter). **e**, Cilium-related DEGs in IP fractions of CRS and WAS.

We performed RNA sequencing (RNA-seq) and proteomics of amygdala samples to explore molecular mechanisms that may contribute to stress-related behavioural changes (Fig. 1). Astrocyte-specific RNA-seq of CRS and WAS mice (relative to US mice) revealed 24 downregulated and 29 upregulated differentially expressed genes (DEGs) in CRS mice, and 86 downregulated and 41 upregulated DEGs in WAS mice (absolute log_2_[stress/US] > 1 and adjusted *P* < 0.05; Fig. 2a, Extended Data Fig. 2a–c and Supplementary Table 2). Astrocyte-enriched DEGs were mostly downregulated and, unexpectedly, many downregulated DEGs were related to primary cilia (for example, *Nme8*, *Myoc*, *Cfap53i* and *Iqca*; Fig. 2b,c; *n* = 4 mice per sample, 3–4 samples per group). The primary cilium is a GPCR-laden antenna-like membrane protrusion that adorns cells, including astrocytes^5,35^, and integrates extracellular signals. Pathway analyses of downregulated DEGs in astrocytes (adjusted *P* < 0.05) revealed enrichment of pathways related to cilium assembly, movement and organization for both CRS and WAS mice (Fig. 2c and Extended Data Fig. 2d). This result underscores recent findings on astrocyte primary cilium biology^5,35^. Heatmaps for cilium-related DEGs in CRS and WAS mice are shown in Extended Data Fig. 2e.

**Extended Data Fig. 3:**
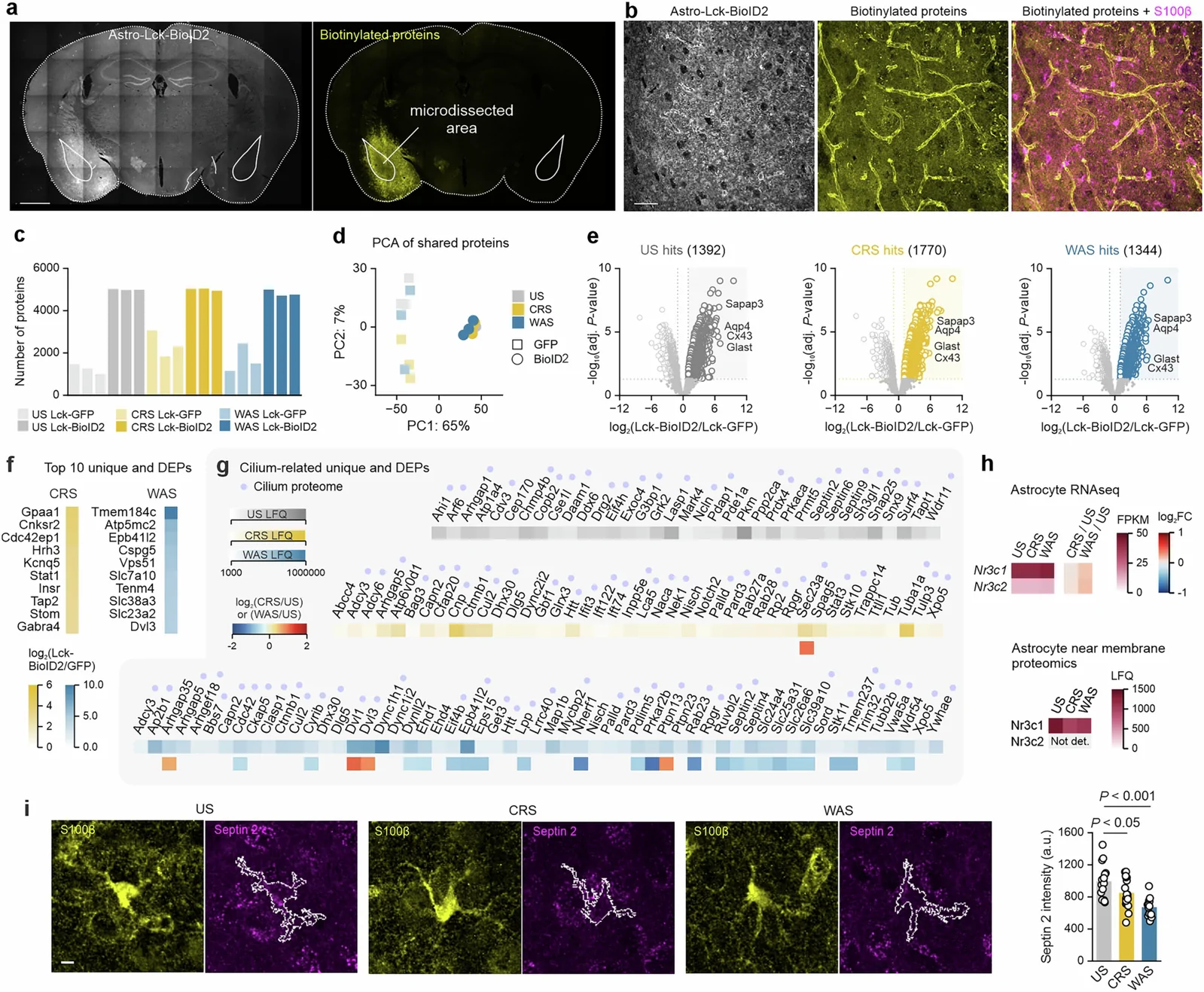
Additional near-membrane proteomics analyses and validations of BLA astrocytes following CRS and WAS. **a**, Representative images of immunostained brain sections showing *GfaABC*_*1*_*D*-Lck-BioID2 injection in BLA for micro-dissection and proteomic analyses (n = 4 mice). Scale bar: 1 mm. **b**, Representative images of immunostained brain sections showing *GfaABC*_*1*_*D*-Lck-BioID2 (immunodetected by HA tag) in white, biotinylated proteins (detected by streptavidin) in yellow, and S100β in magenta (n = 4 mice). Scale bar: 40 µm. **c**, Number of proteins detected in each group. **d**, PCA plot of US, CRS and WAS proteomics dataset. **e**, Volcano plot of hits of US, CRS and WAS groups. Hits were obtained with limma. **f**, Top unique and differentially expressed proteins (DEPs) in CRS and WAS mice. **g**, Cilium-related unique and DE proteins of US, CRS and WAS groups. **h**, Gene expression and protein levels of Nr3c1 and Nr3c2 in US, CRS and WAS BLA astrocytes. **i**, Representative images of IHC validation of a down-regulated DEP, Septin2, in US, CRS and WAS. Septin2 is shown in yellow and S100β in magenta. Quantification of Septin2 intensity (n = 4 mice per group; *P* values v US: CRS *P* = 4.85 × 10^−2^, WAS *P* < 1 × 10^−4^). Scale bar: 5 µm. Bars represent the mean and error bars represent the s.e.m. Statistical tests and values are reported in Supplementary Table 1. Source data

We next performed near-plasma membrane proteomics for amygdala astrocytes using LCK–BioID2 (Fig. 2d and Extended Data Fig. 3a–e; *n* = 8 mice per sample, 4 samples per group). We identified about 1,500 proteins in relation to endogenously biotinylated proteins with parallel controls for US, CRS and WAS groups (log_2_[BioID2/GFP] > 1 and adjusted *P* < 0.05; Fig. 2e, Extended Data Fig. 3c–e and Supplementary Table 3). These proteins represent those unique to the experimental groups and those shared between them, which can be differentially expressed proteins (DEPs) (Fig. 2e–g). In CRS mice, 620 proteins were unique, whereas 315 proteins were unique to WAS mice. Among the shared proteins, 18 were DEPs in CRS mice compared with US mice, and 271 were DEPs for WAS mice compared with US mice (Fig. 2f; absolute log_2_[stress/US] > 0.5 and adjusted *P* < 0.1). Consistent with the RNA-seq findings (Fig. 2a–c), analyses of DEPs revealed cilium-related proteins unique to each group or differentially expressed compared with the US group (Extended Data Fig. 3f,g). In contrast to the cilium-related genes and proteins, changes in corticosteroid *Nr3c1* and *Nr3c2* receptors, which are often implicated in stress responses, were not observed (Extended Data Fig. 3h). Downregulation of cilium-related protein SEPTIN2 (encoded by *Septin2*; also known as SEPT2) in CRS and WAS mice was confirmed by IHC (Extended Data Fig. 3i).

## Amygdala astrocytes have primary cilia

**Extended Data Fig. 4:**
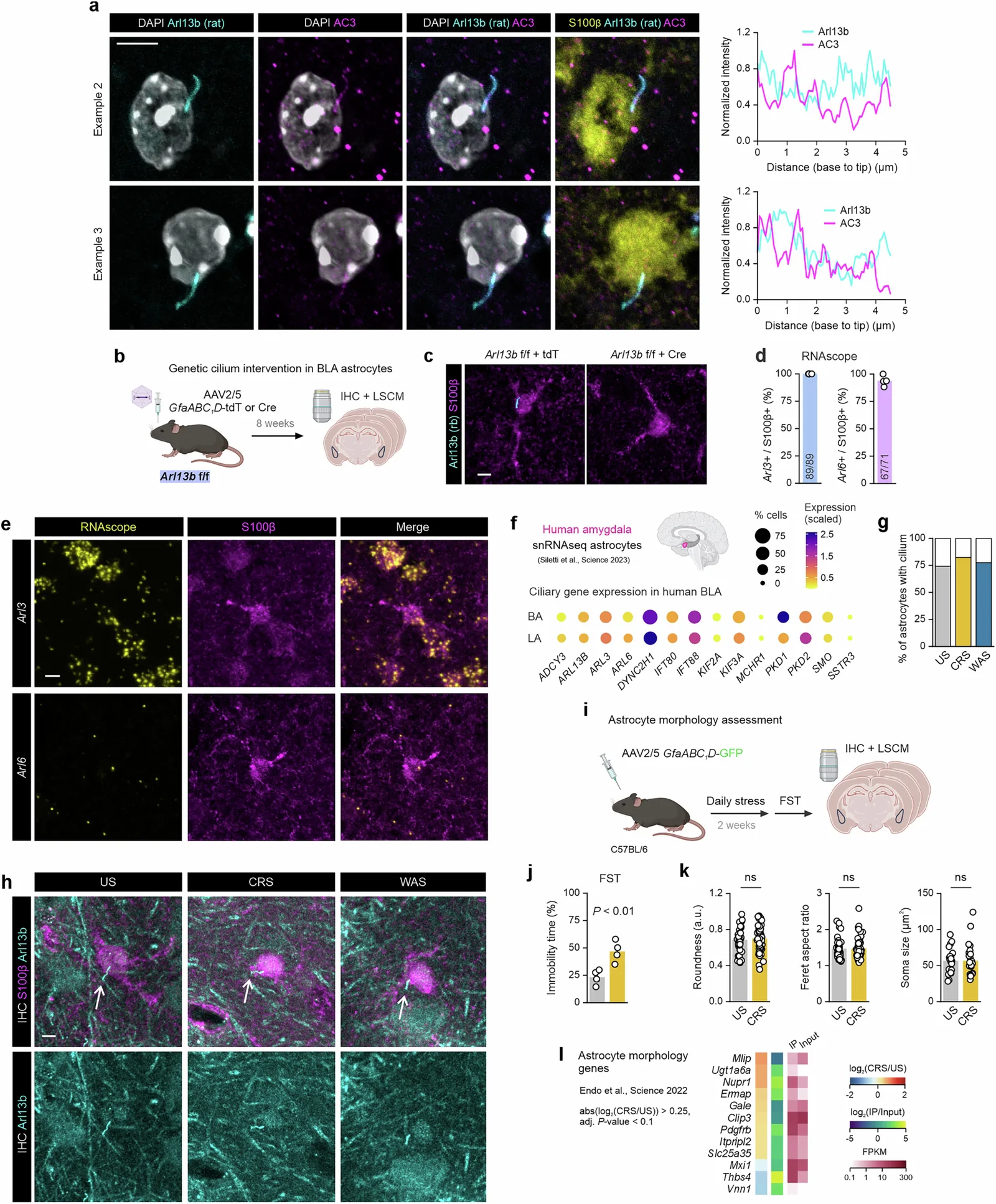
Additional BLA astrocyte cilium and morphology validations. **a**, More representative examples of high-resolution confocal imaging of the Arl13b and AC3-stained primary cilium in a BLA astrocyte (left) and line analysis of Arl13b and AC3 staining in each image (right) (n = 4 mice). Scale bar: 5 µm. **b**, Cartoon depicting the strategy for genetic cilium intervention in BLA astrocytes in *Arl13b*^*f/f*^ mice. **c**, Representative images of BLA astrocytes from *Arl13b*^*f/f*^ mice that received tdT (top) or Cre (bottom). Note that astrocytes in the Cre group no longer possess an Arl13b+ or AC3+ primary cilium. The quantification is shown in Fig. 2o (n = 3 mice per group). Scale bar: 5 µm. **d**, Quantification of *Arl3*+ and *Arl6*+ RNAscope in astrocytes (n = 4 mice per gene; numbers represent expression positive cells/total cells). **e**, Combined RNAscope and IHC of *Arl3* (yellow, RNAscope, top panel), *Arl6* (yellow, RNAscope, bottom panel) and S100β (magenta, IHC, both panels) in mouse BLA tissue (n = 4 mice per gene). Scale bar: 5 µm. **f**, Expression of cilium-related genes in human BLA (data from ref. ^57^). **g**, Percentage of ciliated astrocytes (n = 4 mice per group). **h**, Representative images of immunostained BLA from US, CRS and WAS mice showing S100β in magenta (astrocytes) and Arl13b in cyan (cilium) that were used to generate 3D rendered images in Fig. 2p (n = 4 mice per group). Scale bar: 4 µm. **i**, Cartoon illustrating the strategy used to label astrocytes for morphology analysis using AAVs to deliver *GfaABC*_*1*_*D*-GFP to the amygdala. **j**, Immobility time in forced swim test of mice used for astrocyte morphology analyses (n = 4 mice per group). **k**, Roundness, Feret aspect ratio (# astrocytes = 46 US, 53 CRS; n = 4 mice per group) and soma size (# astrocytes = 22 US, 25 CRS; n = 4 mice per group). **l**, Expression levels of morphology-related genes in RNAseq of CRS astrocytes. Bars represent the mean and error bars represent the s.e.m. Statistical tests and values are reported in Supplementary Table 1. Icons in **b**,** f** and** i** created in BioRender; Gutierrez Pelaz, S. https://biorender.com/evoeh1p (2026). Source data

To determine whether changes in cilium-related gene and protein expression following CRS and WAS (Fig. 2a–g) affect astrocyte primary cilia, we performed IHC using the cilium-enriched markers ARL13B and AC3 (refs. ^53–56^). We identified ARL13B-positive primary cilia emerging from S100β-positive amygdala astrocytes (Fig. 2h and Extended Data Fig. 4a). Consistent with past studies^34^, the primary cilium marker AC3 was located in the same structures but with patterns dissimilar to ARL13B. This result was apparent from line profiles (Fig. 2i), areas of ARL13B and AC3 staining (Fig. 2j) and their overlap (Fig. 2k). The more uniform expression of ARL13B enabled us to estimate the length of astrocyte primary cilia to be around 5 μm (Fig. 2l), which is consistent with previous reports^34^. To further validate the use of IHC to image astrocytic primary cilia, we selectively disrupted amygdala astrocyte primary cilia in *Arl13b*^*f/f*^ mice and *Ift88*^*f/f*^ mice^5^ injected with astrocyte-specific Cre adeno-associated viruses (AAVs) or control tdTomato AAVs (Fig. 2m). ARL13B is a small GTPase, whereas IFT88 is a core protein of the intraflagellar transport machinery that traffics proteins in and out of the cilium. Both are required for ciliary signalling, and genetic-mediated depletion of either causes cilial disruption^5^. ARL13B and AC3 immunostaining was observed in S100β-positive astrocytes from tdTomato mice, but not from Cre mice (Fig. 2n). Accordingly, the proportion of astrocytes with primary cilia decreased from >80% to about 10% in mice depleted of ARL13B and IFT88 (Fig. 2o and Extended Data Fig. 4b,c). RNAscope analyses of two cilium genes (*Arl3* and *Arl6*) revealed their expression in around 95% of amygdala S100β-positive astrocytes (Extended Data Fig. 4d,e). Furthermore, primary-cilium-related genes, including *Arl13b* and *Ift88*, were abundantly expressed in human postmortem amygdala astrocytes from published data^57^ (Extended Data Fig. 4f). Our validations with three antibody markers (rat anti-ARL13B, rabbit anti-ARL13B and anti-AC3) in control mice and in mice with genetic disruption of astrocytic primary cilia (*Arl13b* and *Ift88* deletions), as well as evaluations of gene expression in mice and humans (Fig. 2h–o and Extended Data Fig. 4a–f), provide evidence that amygdala astrocytes possess primary cilia.

## Stress-induced astrocyte primary cilium changes

**Extended Data Fig. 5:**
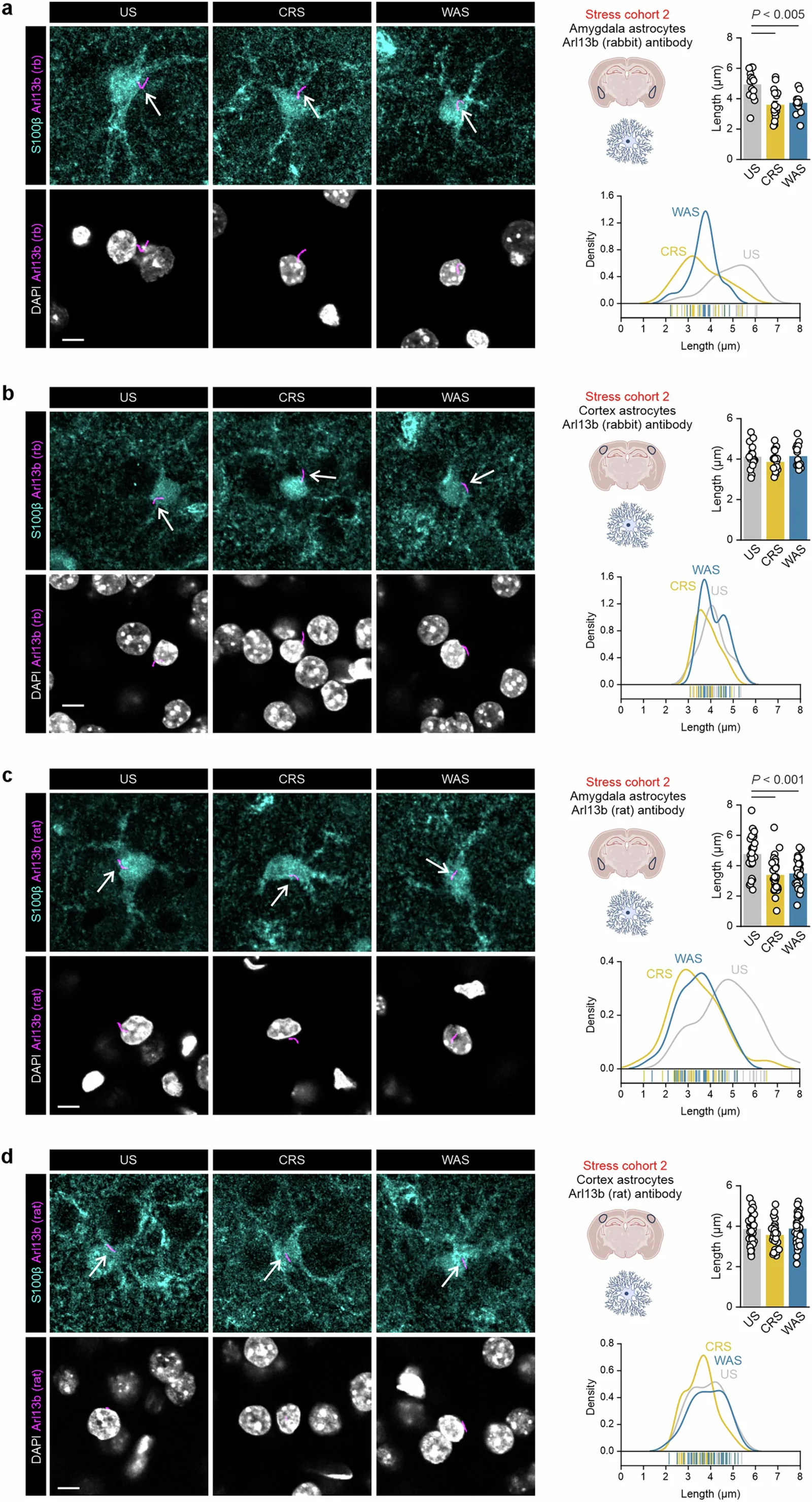
Primary cilium length in astrocytes from an additional cohort of stressed mice, with two different Arl13b antibodies and in two different brain regions (BLA and somatosensory cortex). **a**, **b**, Representative images of immunostained BLA (**a**) or somatosensory cortex (**b**) astrocytes from a separate second cohort of US, CRS and WAS mice showing S100β in cyan (astrocytes) and Arl13b (rabbit antibody) in magenta (cilium). n = 14-18 astrocytes from 4 mice per group. *P* values of panel a (v US): CRS *P* = 2.70 × 10^−4^, WAS *P* = 1.02 × 10^−3^. The bar plot and density plot show the length of the primary cilium based on Arl13b staining. Scale bar: 5 µm. **c,d**, Same as **a** and **b** but using an Arl13b rat antibody. **c** is for BLA astrocytes and **d** is for somatosensory cortex astrocytes. n = 27-31 astrocytes from 4 mice per group. *P* values of panel c (v US): CRS *P* < 1 × 10^−4^, WAS *P* = 1.32 × 10^−4^. Bars represent the mean and error bars represent the s.e.m. Statistical tests and values are reported in Supplementary Table 1. Icons in **a**–**d** created in BioRender; Gutierrez Pelaz, S. https://biorender.com/evoeh1p (2026). Source data

**Extended Data Fig. 6:**
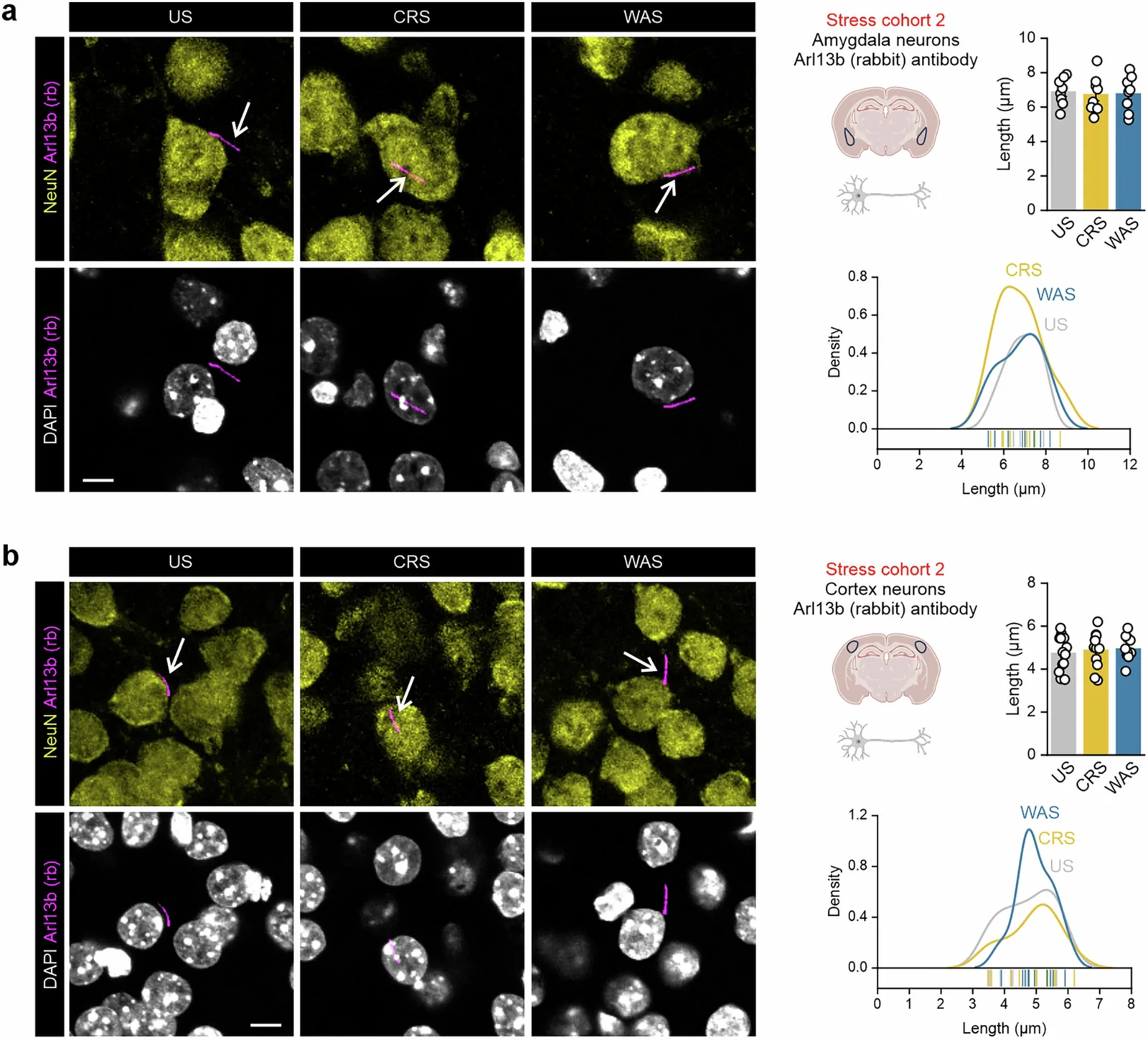
Primary cilium length in neurons from an additional cohort of stressed mice in two different brain regions (BLA and somatosensory cortex). **a, b**, Representative images of immunostained BLA (**a**) or somatosensory cortex (**b**) neurons from a separate second cohort of US, CRS and WAS mice showing NeuN in yellow (neurons) and Arl13b (rabbit antibody) in magenta (cilium). n = 8-13 neurons from 4 mice per group. The bar plot and density plot show the length of the primary cilium based on Arl13b staining. Scale bar: 5 µm bars represent the mean and error bars represent the s.e.m. Statistical tests and values are reported in Supplementary Table 1. Icons in **a** and **b** created in BioRender; Gutierrez Pelaz, S. https://biorender.com/evoeh1p (2026). Source data

IHC analyses revealed about 80% of amygdala astrocytes possessed ARL13B-positive cilia in US, CRS and WAS groups (Extended Data Fig. 4g; *n* = 4 mice per group). However, the lengths and volumes of astrocyte primary cilia were significantly reduced after CRS or WAS (Fig. 2p–s and Extended Data Fig. 4h; *n* = 4 mice per group). By contrast, ARL13B-positive cilia of neurons did not change in length (Fig. 2t; *n *= 4 mice per group). Furthermore, in a separate cohort of mice, CRS and WAS again resulted in shorter primary cilia on amygdala astrocytes, whereas primary cilia of cortical astrocytes were unaffected (Extended Data Fig. 5a–d; *n* = 4 mice per group with rabbit or rat ARL13B antibody IHC). Neuronal primary cilia in the amygdala or cortex were also unaffected (Extended Data Fig. 6a,b; *n* = 4 mice per group). As amygdala astrocyte primary cilia were reduced in length after stress, we determined whether astrocyte morphology^58^ was also altered after CRS; however, this was not so (Fig. 2u and Extended Data Fig. 4i–l; *n* = 4 mice per group). We also did not find marked changes in the expression of genes related to astrocyte territory size^58^ after stress (Extended Data Fig. 4l). However, of the 770 primary cilium-related genes (Supplementary Table 4), 121 and 124 were altered in amygdala astrocyte DEGs (adjusted *P* < 0.05) after CRS and WAS, respectively, when assessed by RNA-seq (Fig. 2v). Our evaluations provide evidence that amygdala astrocyte primary cilia are significantly smaller following stress in mice. Moreover, these changes are accompanied by molecular alterations of primary cilium genes and proteins (Fig. 2a–t).

**Extended Data Fig. 7:**
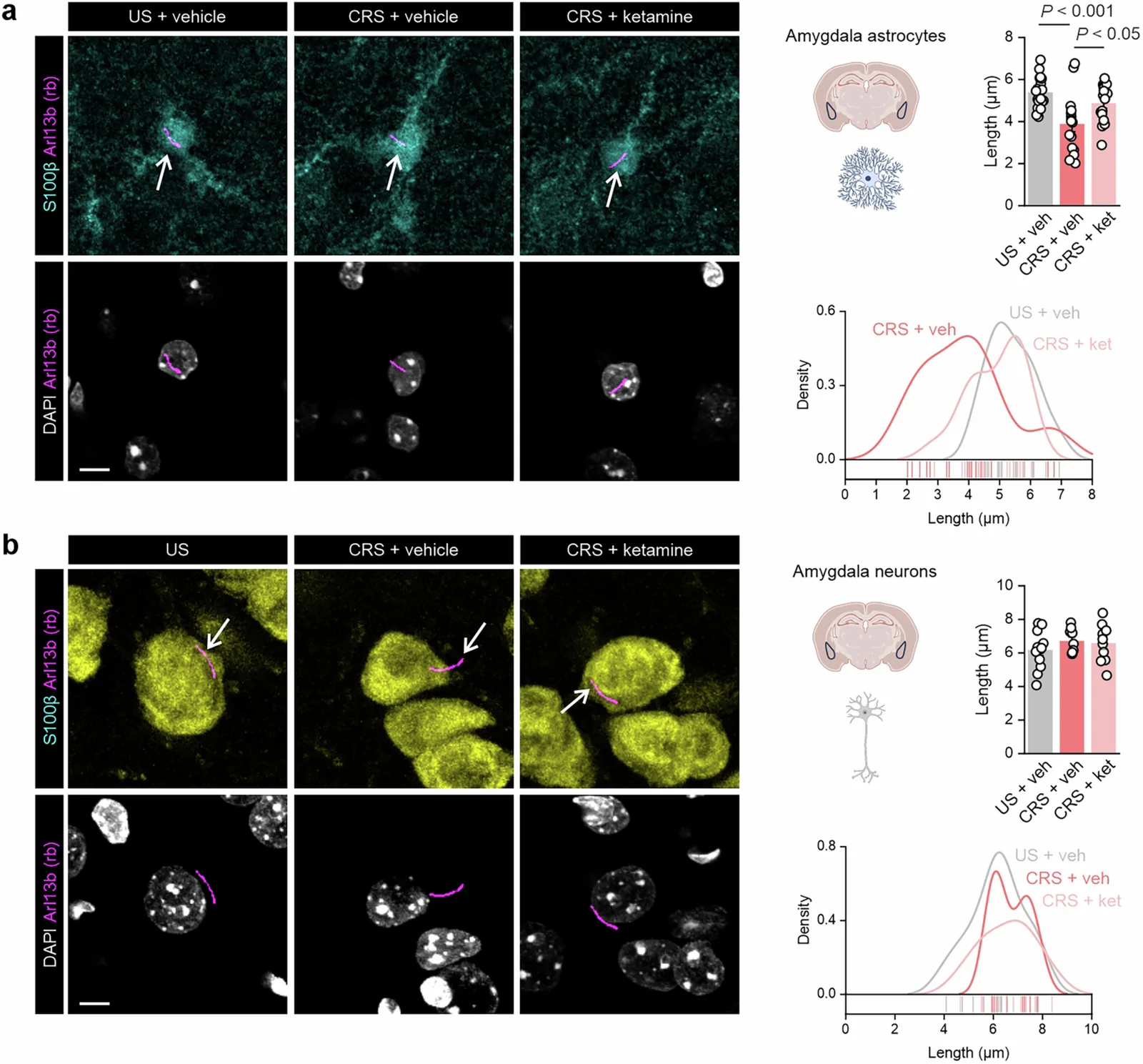
Ketamine restores CRS-induced shortening of primary cilium length in BLA astrocytes. **a, b**, Representative images of immunostained BLA astrocytes from US + vehicle, CRS + vehicle and CRS + ketamine mice showing S100β in cyan (astrocytes, a), NeuN in yellow (neurons, b) and Arl13b (rabbit antibody) in magenta (cilium). n = 16-18 astrocytes from 4 mice per group and 9-13 neurons from 4 mice per group. The bar plots and density plots show the length of the primary cilium for every group and cell based on Arl13b staining. Scale bar: 5 µm. Bars represent the mean and error bars (sometimes obscured by the data points) represent the standard error of the mean. Statistical tests and values are reported in Supplementary Table 1. Icons in **a** and **b** created in BioRender; Gutierrez Pelaz, S. https://biorender.com/evoeh1p (2026). Source data

We also performed a specific set of experiments to assess the effects of ketamine, which reduced some of the stress-related behavioural changes (Extended Data Fig. 1f,g). The effects of ketamine were accompanied by significant restoration of amygdala astrocyte primary cilium length. Conversely, no effects were observed for neuronal primary cilia (Extended Data Fig. 7). We do not interpret this result to suggest that ketamine works via the astrocyte primary cilium, but instead that the astrocyte primary cilium is recovered during the beneficial response to ketamine.

## Stress and neuroinflammation

**Extended Data Fig. 8:**
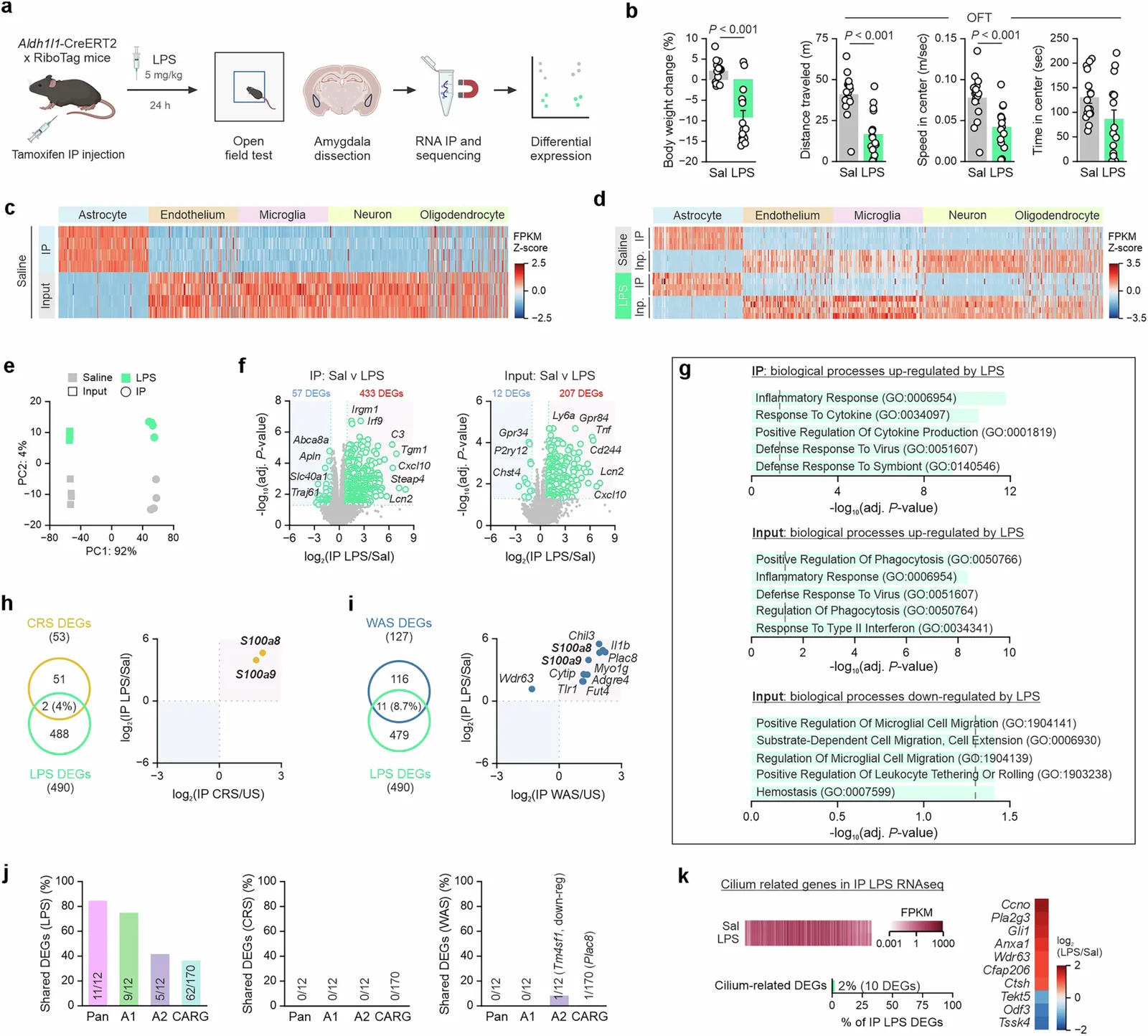
Comparison of neuroinflammation model to stress models. **a**, Cartoon illustrating the experiment. *Aldh1l1*^*cre/ERT2*^;RiboTag^2^ mice underwent peripherally induced neuroinflammation as in Diaz-Castro et al.^78^ and the amygdala was micro-dissected for RNAseq. **b**, Changes in body weight and open field test metrics after induction of neuroinflammation (n = 8 mice per group; *P* values v saline: body weight *P* = 1.12 × 10^−4^, OFT distance *P* < 0.0001, OFT speed *P* = 7.77 × 10^−4^). **c**, Expression of a panel of cell-type enriched genes^102^ in the IP and input samples from the saline group. **d**, Expression of a panel of cell-type enriched genes from Barres’ markers^102^ in the IP and input samples from the saline and LPS groups. **e**, PCA plot of the neuroinflammation RNAseq dataset. **f**, Volcano plot of saline v LPS groups for IP and input samples. DEGs were obtained with limma-voom. **g**, Biological processes (obtained with Enrichr, Fisher’s exact test followed by Benjamini-Hochberg) of IP and input DEGs. Those downregulated in the IP samples are not shown because they were not statistically significant. **h, i**, Overlap of CRS (h) or WAS (i) DEGs and LPS DEGs and correlation plot (DEGs defined by log_2_ fold change > 1 and adj. P-value < 0.05). Red area means up-regulated in both groups and blue area down-regulated in both groups. **j**, Overlap between astrocyte DEGs from two published neuroinflammation datasets^103,104^ and our astrocyte DEGs for neuroinflammation and stress. **k**, Changes in cilium-related genes in BLA astrocytes from the neuroinflammation model. DEGs found in our cilium-related gene list are shown in the heatmap (10 out of 490 DEGs, defined by log_2_ fold change > 1 and adj. *P*-value < 0.05). Bars represent the mean and error bars represent the s.e.m. Statistical tests and values are reported in Supplementary Table 1. Icons in **a** created in BioRender; Gutierrez Pelaz, S. https://biorender.com/evoeh1p (2026). Source data

**Extended Data Fig. 9:**
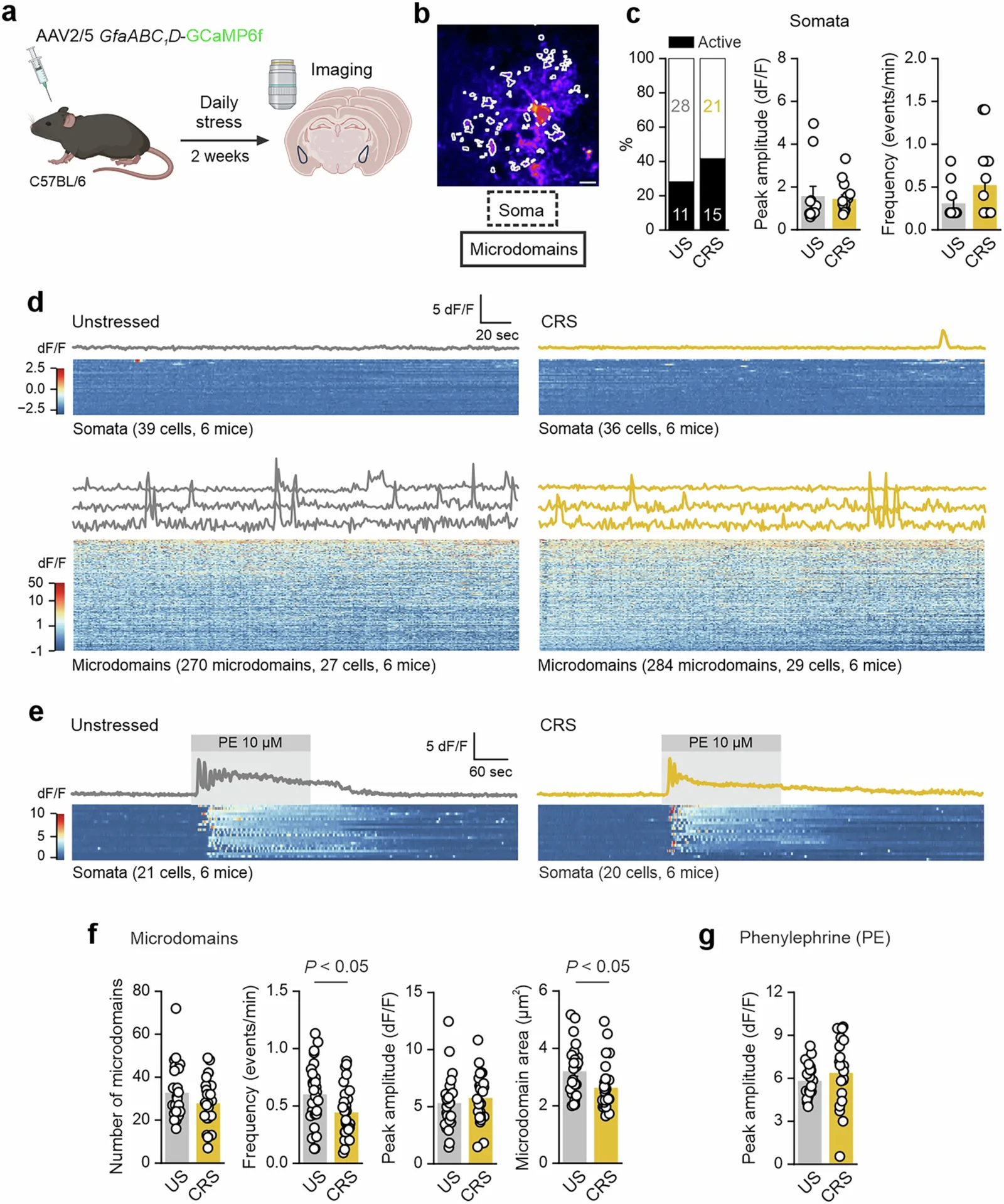
Amygdala astrocyte Ca^2+^ signaling was largely normal following CRS, but astrocytes displayed fewer Ca^2+^ microdomains. **a**, Cartoon of the protocol. **b**, Representative image showing ROIs for a soma and microdomains (detected using GECIquant; see Methods). **c**, Summary data for somatic signals showed no differences between US and CRS groups during 300 s long imaging sessions. **d**, Representative traces for somatic and microdomain Ca^2+^ signals in US and CRS mice. **e**, Representative traces for 10 μM phenylephrine-evoked (PE) Ca^2+^ signals from US and CRS mice. **f**, Average data for experiments shown in d (*P* values v US: frequency *P* = 2.31 × 10^−2^, area *P* = 1.41 × 10^−2^). **g**, Average data for experiments shown in e. The data shown are from n = 6 mice per group. Bars represent the mean and error bars represent the s.e.m. Statistical tests and values are reported in Supplementary Table 1. Icons in **a** created in BioRender; Gutierrez Pelaz, S. https://biorender.com/evoeh1p (2026). Source data

Neuroinflammation and astrogliosis are often associated with brain disorders^49^. However, the decreased GFAP expression (Fig. 1h,k,l) and normal astrocyte morphology (Fig. 2u) following stress observed here suggested a lack of astrogliosis and neuroinflammation, which are often associated with elevated GFAP and hypertrophy^49^. To explore the overlap between CRS, WAS and neuroinflammation more directly, we induced neuroinflammation in the absence of stress using bacterial lipopolysaccharide (LPS; 5 mg kg^–1^). This treatment induced sickness behaviour and expected neuroinflammation-related changes in astrocyte gene expression (Extended Data Fig. 8a–g and Supplementary Table 5). However, there was little evidence to indicate that the gene expression changes following CRS or WAS were related to those following LPS (Extended Data Fig. 8h–j). Furthermore, primary cilium-related genes were mostly unaltered by LPS (Extended Data Fig. 8k), which indicated that following stress, amygdala astrocytes display changes (Figs. 1 and 2) in the absence of overt astrogliosis. Astrocyte intracellular Ca^2+^ signalling changes are observed in some disease models^7,59^. Although somatic spontaneous and phenylephrine-evoked Ca^2+^ signals were normal following CRS, we recorded decreased spontaneous microdomain Ca^2+^ signalling in astrocyte territories (Extended Data Fig. 9a–g). Taking into account the large body of data on astrocyte Ca^2+^ signalling in behaviour and disease settings^7,59^, the effects of CRS were relatively small (Extended Data Fig. 9).

## GPCR signalling improves stress behaviours

**Fig. 3:**
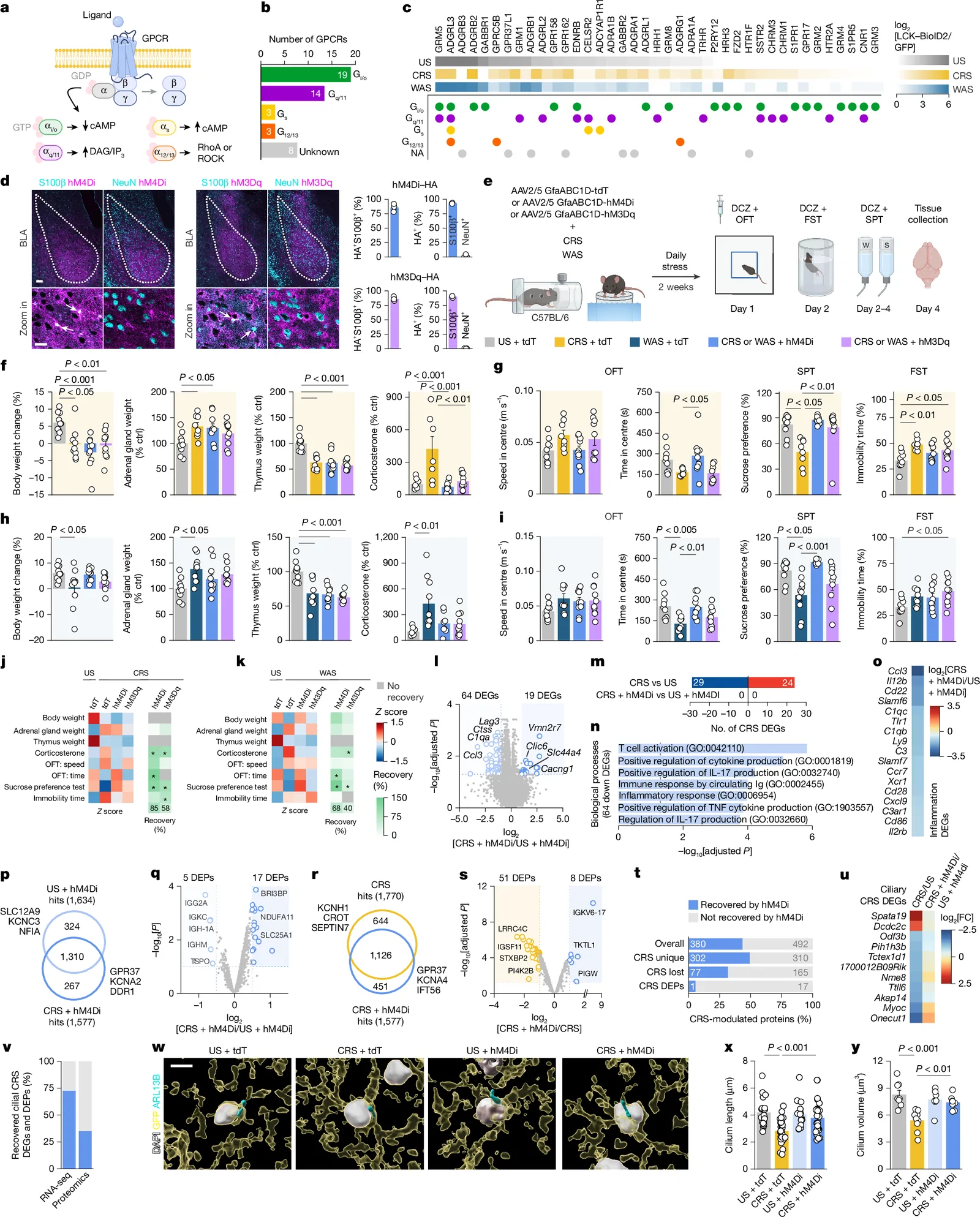
G_i_–GPCR signalling rescues astrocytic stress-related behavioural and molecular changes. **a**, Schematic of coupling and signalling of GPCRs. **b**,**c**, Number of GPCRs identified from amygdala astrocyte near-membrane proteomics (**b**) and downstream coupling relationships (**c**). NA, not available. **d**, Left, representative images of immunostained amygdala (dashed line) that had received astrocyte-specific DREADDs. The images show astrocytes or neurons (white arrows) in cyan as indicated and hM4Di (left) or hM3Dq (right) in magenta. Right, bar plots showing astrocyte penetrance and specificity for both DREADDs (*n* = 3 mice per group). Scale bars, 100 µm (top), 25 µm (bottom). **e**, Astrocyte-specific DREADDs were delivered to the amygdala and mice underwent stress and subsequent evaluations while receiving the DREADD agonist deschloroclozapine (DCZ) daily for 4 days. **f**, Physiological metrics for CRS groups. *n* (mice) = 8–10 (US + tdT), 9 (CRS + tdT), 10 (CRS + hM4Di) and 10 (CRS + hM3Dq). *P* values versus US + tdT: body weight CRS + tdT *P* = 1.21 × 10^−2^, CRS + hM4Di *P* = 2.31 × 10^−4^, CRS + hM3Dq *P* = 5.4 × 10^−3^; adrenal gland weight CRS + tdT *P* = 2.48 × 10^−2^, CRS + hM4Di *P* = 3.03 × 10^−2^; thymus weight CRS + tdT, CRS + hM4Di, CRS + hM3Dq *P* < 1 × 10^−4^; corticosterone weight US + tdT versus CRS + tdT *P* = 8.024 × 10^−4^, CRS + tdT versus CRS + hM4Di *P* = 1.47 × 10^−4^ and CRS + tdT versus CRS + hM3Dq *P* = 1.14 × 10^−3^. **g**, Behavioural metrics for CRS groups. *n* (mice) = 8–10 (US + tdT), 9 (CRS + tdT), 10 (CRS + hM4Di) and 10 (CRS + hM3Dq). *P* values: OFT time CRS + tdT versus CRS + hM4Di *P* = 3.21 × 10^−2^; SPT US + tdT versus CRS + tdT *P* = 1.21 × 10^−2^; CRS + tdT versus CRS + hM4Di *P* = 2.55 × 10^−3^; CRS + tdT versus CRS + hM3Dq *P* = 1.464 × 10^−2^; FST US + tdT versus CRS + tdT *P* = 1.53 × 10^−3^; US + tdT versus CRS + hM3Dq *P* = 4.38 × 10^−2^. **h**, Physiological metrics for WAS groups. *n* (mice) = 8–10 (US + dT), 10 (WAS + tdT), 8–10 (WAS + hM4Di) and 10 (WAS + hM3Dq). *P* values versus US + tdT: body weight WAS + tdT *P* = 3.806 × 10^−2^; adrenal gland weight WAS + tdT *P* = 5.91 × 10^−3^; thymus weight WAS + tdT, WAS + hM4Di, WAS + hM3Dq *P* < 1 × 10^−4^; corticosterone weight WAS + tdT *P* = 3.2 × 10^−3^. **i**, Behavioural metrics for WAS groups. *n* (mice) = 9–10 (US + tdT), 10 (WAS + tdT), 10 (WAS + hM4Di) and 10 (WAS + hM3Dq). *P* values: OFT time US + tdT versus WAS + tdT *P* = 3.81 × 10^−3^; WAS + tdT versus WAS + hM4Di *P* = 3.8 × 10^−3^; SPT US + tdT versus WAS + tdT *P* = 3.59 × 10^−2^; WAS + tdT versus WAS + hM4Di *P* < 1 × 10^−4^; FST US + tdT versus WAS + hM3Dq *P* = 1.58 × 10^−2^. **j**,**k**, Heatmaps summarizing changes and recovery in physiological and behavioural readouts after stress with or without DREADD activation in CRS (**j**) and WAS (**k**) groups. **l**, Volcano plot of RiboTag RNA-seq data from amygdala astrocytes after US or CRS, both with hM4Di activation. DEGs were obtained using limma-voom. **m**, DEGs in CRS and US mice and in CRS + hM4Di mice and US + hM4Di mice. **n**, Inflammation-related biological processes downregulated in CRS + hM4Di mice (obtained using Enrichr, Fisher’s exact test followed by Benjamini–Hochberg correction). **o**, DEGs that contributed to the biological processes identified in **n**. **p**, Venn diagram of shared and unique proteomic hits for US + hM4Di and CRS + hM4Di mice. **q**, Volcano plot comparing GfaABC1D–LCK–BioID2 proteomic data from CRS + hM4Di mice and US + hM4Di mice. DEPs were obtained using limma. **r**, Venn diagram for shared and unique proteomic hits for CRS versus CRS + hM4Di mice. **s**, Volcano plot comparing proteomic data from CRS + hM4Di mice with CRS mice. **t**, DEPs recovered by hM4Di activation. **u**, Changes in cilium-related DEGs from **l** in CRS and CRS + hM4Di mice. **v**, Percentage of cilium-related DEGs and DEPs recovered by hM4Di activation. **w**, Representative 3D-rendered images of immunostained astrocytes (GfaABC1D–GFP, yellow) showing the cell nucleus (DAPI, white) and the cilium (ARL13B, cyan). Scale bar, 5 µm. **x**, Astrocyte cilium length measured in IHC images. Number of astrocytes = 20 (US + tdT), 34 (CRS + tdT), 14 (US + hM4Di) and 29 (CRS + hM4Di);* n* = 4 mice per group. *P* values: US + tdT versus CRS + tdT *P* < 1 × 10^−4^; CRS + tdT versus CRS + hM4Di *P* = 1.06 × 10^−3^. **y**, Astrocyte cilium volume measured in 3D-rendered representations based on IHC images. Number of astrocytes = 8 (US + tdT), 9 (CRS + tdT), 6 (US + hM4Di) and 8 (CRS + hM4Di); *n* = 4 mice per group. *P *values: US + tdT versus CRS + tdT *P* = 1.09 × 10^−4^; CRS + tdT versus CRS + hM4Di *P* = 6.83 × 10^−3^. Bars represent the mean and error bars represent the s.e.m. Statistical tests and values are reported in Supplementary Table 1. Icons in **a** and** e** created in BioRender; Gutierrez Pelaz, S. https://biorender.com/evoeh1p (2026). Source data

As we observed reduced astrocyte primary cilium lengths, we next considered signalling pathways that are associated with this organelle. Primary cilia are well-established hubs for GPCR-mediated signalling^8^, and GPCR pathways have key roles in astrocyte function^7^, including in the amygdala^31^. On the basis of this overlap, we analysed our proteomic datasets (Fig. 2d–f) from US, CRS and WAS groups to identify GPCRs expressed in amygdala astrocytes and to determine their predicted G protein coupling^17,60^ (Fig. 3a–c). On the basis of these data, we speculated that activation of GPCR signalling pathways might influence primary cilium morphology. Among the GPCRs detected in astrocytes by proteomics, most were predicted to couple to G_i_ and/or G_q_ proteins^61–64^ (Fig. 3a–c; for example, G_i_-coupled receptors S1PR1, FZD2, GRM2 and GRM3; G_q_-coupled receptors GRM5, GRM1 and SSTR2, CHRM3). These data led us to test whether astrocyte-specific activation of GPCR signalling pathways could influence stress-induced behaviours.

**Extended Data Fig. 10:**
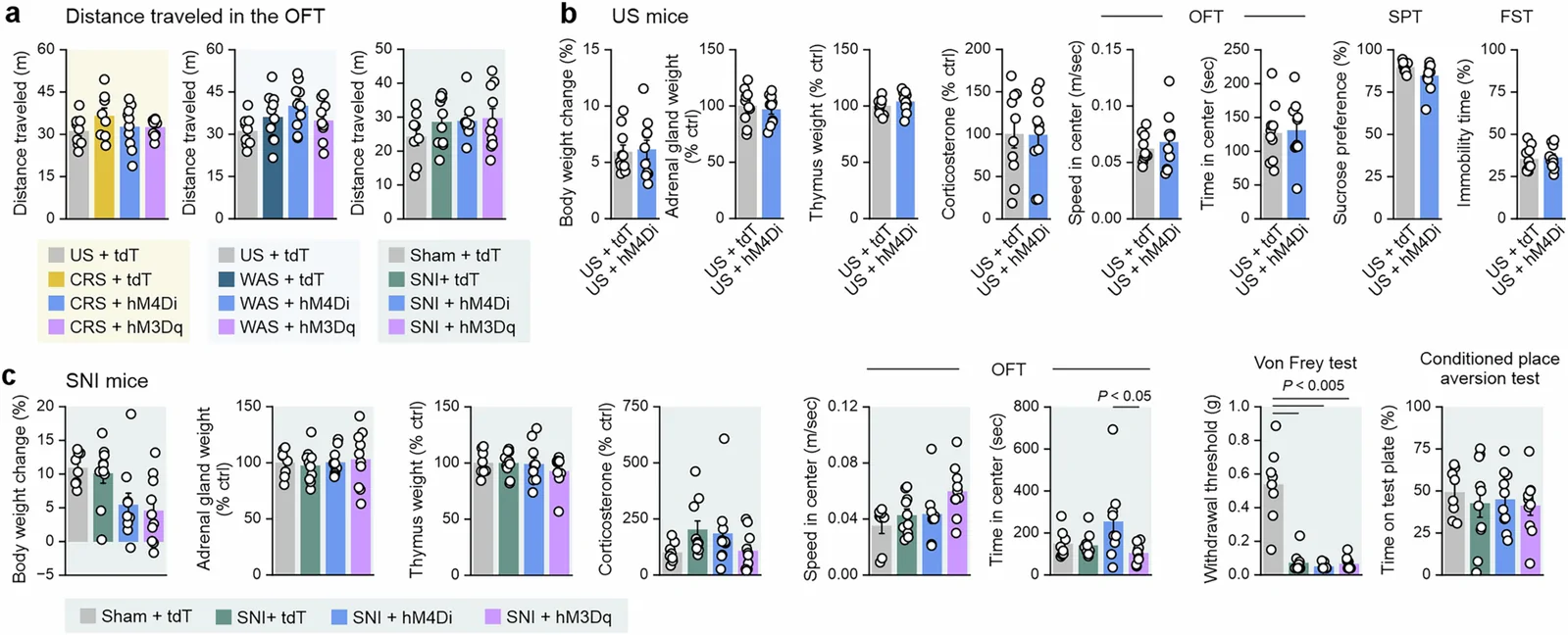
Additional behavioral data and controls for DREADD experiments. **a**, Total distance traveled in OFT. **b**, Changes in body weight, adrenal glands weight, thymus weight, corticosterone, OFT, SPT and FST in US mice after hM4Di activation (n = 10 mice per group). **c**, Changes in body weight, adrenal glands weight, thymus weight, corticosterone, OFT, Von Frey test and conditioned place temperature aversion test in SNI mice after hM4Di activation (n = 8-10 mice per group). Bars represent the mean and error bars represent the s.e.m. Statistical tests and values are reported in Supplementary Table 1. Source data

We expressed hM4Di (G_i_-coupled) and hM3Dq (G_q_-coupled) designer receptors exclusively activated by designer drugs (DREADDs)^65^ in amygdala astrocytes via AAV microinjections (Fig. 3d; *n* = 7–10 mice per group). Two weeks later, the mice were subjected to CRS (Fig. 3e–g) or WAS (Fig. 3e,h,i), followed by physiological and behavioural assessments (as in Fig. 1). Control mice received tdTomato in place of DREADDs, and all the mice received deschloroclozapine (10 μg kg^–1^) in vivo as the DREADD ligand (Fig. 3e). Neither hM4Di nor hM3Dq DREADD activation rescued the decrease in body weight, increase in adrenal gland weight or decrease in thymus weight induced by CRS (Fig. 3f). This result implies that induction of stress and its related physiological deficits are unaffected by amygdala astrocyte GPCR signalling. However, hM4Di activation reduced corticosterone levels, which indicated that astrocytes contribute to this stress-related outcome (Fig. 3f). We also observed significantly improved measures of behaviours of CRS mice in OFTs, SPTs and FSTs after both hM4Di and hM3Dq activation (Fig. 3g and Extended Data Fig. 10a). Similarly, activation of hM4Di and hM3Dq improved stress-related outcomes following WAS (Fig. 3h,I and Extended Data Fig. 10a). To assess hM4Di and hM3Dq GPCR activation across metrics in CRS and WAS, we calculated *z* scores and percentage recovery (Fig. 3j,k) and found that hM4Di activation was the most effective^17^. In terms of controls, hM4Di activation did not affect stress-related physiological and behavioural readouts in US mice (Extended Data Fig. 10b). Moreover, consistent with the lack of stress-related behaviours after SNI (Extended Data Fig. 1b), neither hM4Di nor hM3Dq activation affected any physiological or behavioural readouts after SNI (Extended Data Fig. 10a,c). Therefore, amygdala astrocyte-specific G_i_–GPCR signalling activation significantly improves behavioural responses caused by stress.

## Ciliary changes and improvement in stress

**Extended Data Fig. 11:**
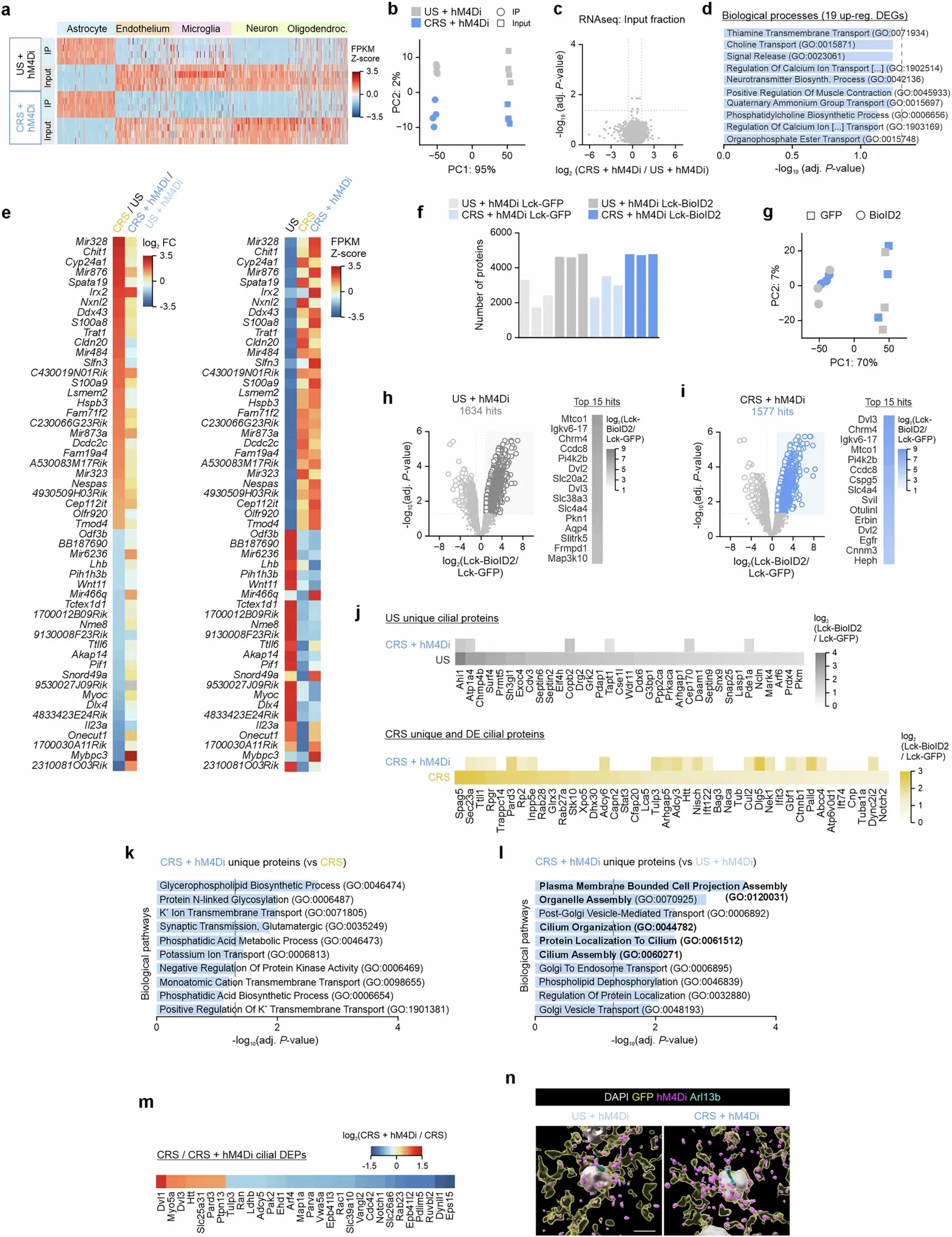
Additional astrocyte RNAseq and near-membrane proteomics analyses following hM4Di activation. **a**, Expression of a panel of cell-type enriched genes^105^. **b**, PCA plot of RNAseq following hM4Di activation**. c**, Volcano plot of Input fraction of US+hM4Di vs CRS+hM4Di RNAseq data. DEGs were obtained with limma-voom. **d**, Biological processes (obtained with Enrichr, Fisher’s exact test followed by Benjamini-Hochberg) of IP up-regulated genes after hM4Di activation. **e**, Change in gene expression of DEGs from US vs CRS upon hM4Di activation. **f**, Number of proteins detected in near-membrane proteomics of BLA astrocytes after hM4Di activation. **g**, PCA plot of near-membrane proteomics following hM4Di activation. **h-i**, Volcano plot of hits of US+hM4Di (h) and CRS+hM4Di groups (i). DEGs were obtained with limma-voom. **j**, Change in protein levels of US and CRS cilium-related unique and DE proteins. **k**, Biological pathways (obtained with Enrichr, Fisher’s exact test followed by Benjamini-Hochberg) of CRS+hM4Di unique proteins (vs US+hM4Di). **l**, Biological pathways (obtained with Enrichr, Fisher’s exact test followed by Benjamini-Hochberg) of CRS+hM4Di unique proteins (vs CRS). **m**, Cilium-related DEPs from CRS vs CRS+hM4Di. **n**, Representative three-dimensional rendered images of immunostained astrocytes (*GfaABC*_*1*_*D*-GFP, yellow) showing hM4Di (magenta), the cell nucleus (DAPI, white) and the cilium (Arl13b, cyan). Scale bar: 5 µm.

**Extended Data Fig. 12:**
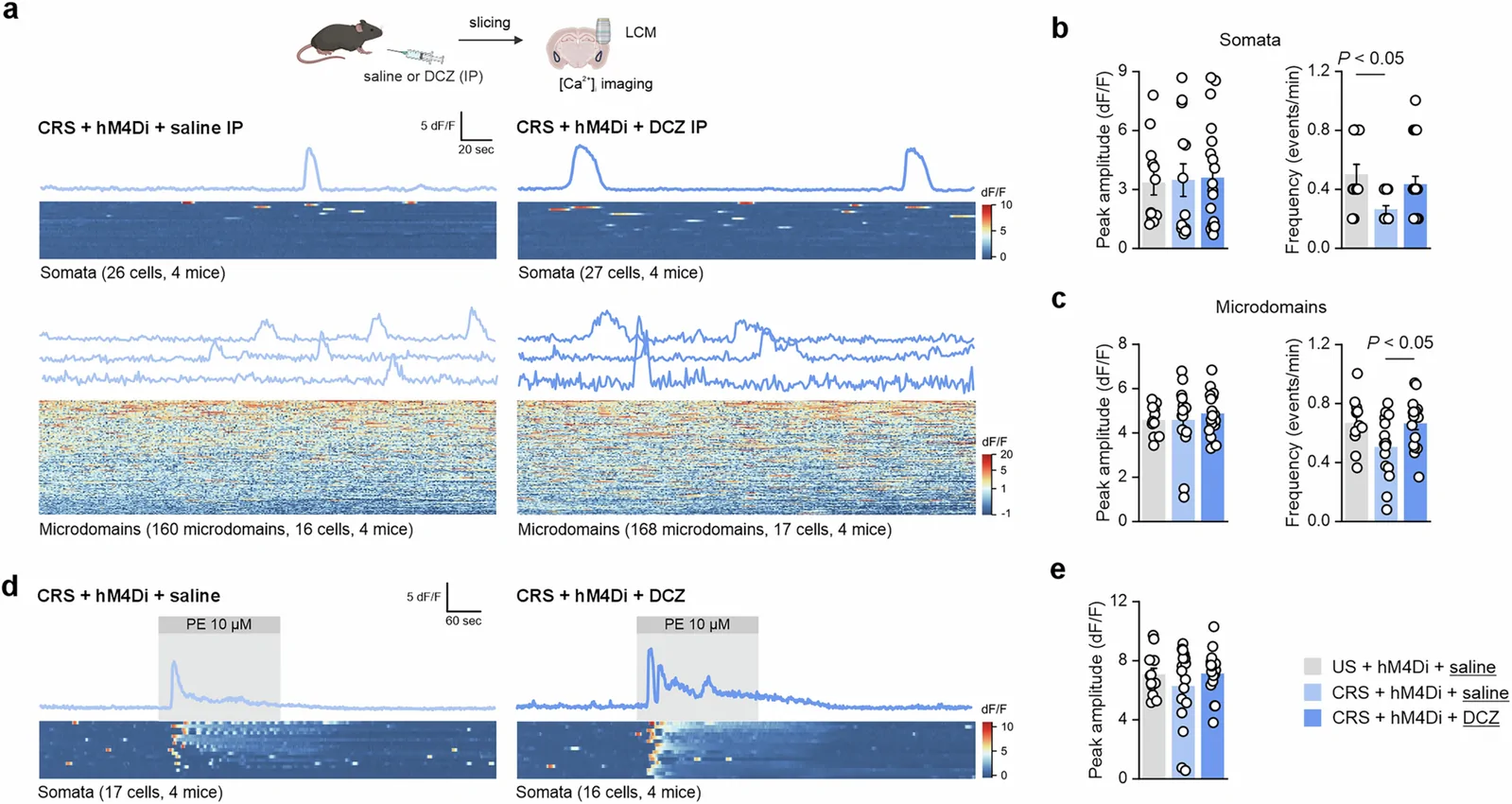
In vivo hM4Di activation with DCZ increased the frequency of microdomain Ca^2+^ signals in amygdala astrocytes from CRS mice relative to saline controls. **a**, WT mice received GCaMP6f as in Extended Data Fig. 9, followed by two weeks of CRS. The day after the last CRS day, they were injected with saline or DCZ and sacrificed for imaging one hour later. Representative traces for somatic and microdomain Ca^2+^ signals in US and CRS mice following hM4Di activation. **b, c**, Average data for experiments shown in a (*P* value US + hM4Di + saline v CRS + hM4Di + saline soma frequency *P* = 1.14 × 10^−2^, CRS + hM4Di + saline v DCZ microdomain frequency *P* = 4.39 × 10^−2^). **d**, Representative traces for 10 μM phenylephrine-evoked (PE) Ca^2+^ signals from US and CRS mice following hM4Di activation. **e**, Average data for experiments shown in d. The data shown are from n = 4 mice per group. Bars represent the mean and error bars represent the s.e.m. Statistical tests and values are reported in Supplementary Table 1. Icons in **a** created in BioRender; Gutierrez Pelaz, S. https://biorender.com/evoeh1p (2026). Source data

We next used astrocyte-specific RNA-seq and near-plasma membrane proteomics to explore the molecular changes induced by hM4Di activation following stress (Fig. 3l–u and Supplementary Table 5). We focused on CRS, because this offered clear improvement by hM4Di activation (Fig. 3j). Astrocyte RNA-seq of CRS and US mice (both following hM4Di activation) revealed 83 DEGs (Fig. 3l and Extended Data Fig. 11a–d), and all the DEGs from the CRS condition were no longer DEGs after hM4Di was activated (Fig. 3m and Extended Data Fig. 11e). Pathways from the upregulated DEGs were diverse, whereas those from the downregulated DEGs (that is, lower in the CRS + hM4Di group) converged on inflammatory processes (Fig. 3n,o), which indicated that hM4Di activation reduced astrocyte neuroinflammatory signalling^17^. Consistent with this, astrocyte near-plasma membrane proteomics of the same experimental groups showed a similar trend (Fig. 3p–s, Extended Data Fig. 11f–i and Supplementary Table 5). Furthermore, around 50% of the DEGs and DEPs in the CRS group were no longer so in the CRS + hM4Di group (Fig. 3t–v and Extended Data Fig. 11j). Compared with CRS alone, the unique proteins found in the CRS + hM4Di group (absent in the CRS group) were related to pathways such as biosynthetic processes, K^+^ transport and glutamatergic synaptic transmission (Extended Data Fig. 11k), which implied that these pathways are induced by hM4Di activation. Notably, proteins found in the CRS + hM4Di group but absent in the US + hM4Di group were related to cilium-related pathways (Extended Data Fig. 11k–m). Moreover, 75% and 30% of primary cilium-related DEGs and DEPs, respectively, measured in the CRS group were rescued after hM4Di activation (Fig. 3v). To evaluate whether recovery of primary cilium-related genes and proteins affected the reduced primary cilium length following stress (Fig. 2), we evaluated primary cilium characteristics in the CRS group after hM4Di activation. The reduction in primary cilium length and volume observed in CRS was completely rescued by astrocyte hM4Di activation (Fig. 3w–y and Extended Data Fig. 11n; *n* = 4 mice per group). Astrocyte microdomain Ca^2+^ signals were slightly reduced in CRS (Extended Data Fig. 9). By contrast, hM4Di activation led to increased numbers of microdomain Ca^2+^ signals relative to saline, which returned to control levels with no effect on phenylephrine-evoked Ca^2+^ signals (Extended Data Fig. 12a–e; *n* = 4 mice per group). Thus, in vivo G_i_–GPCR activation restores multiple effects triggered by stress, including stress-related behaviours, changes in primary cilium-related gene and protein expression and astrocyte primary cilium length and volume (Fig. 3).

## S1PR1 activation is beneficial in stress

**Fig. 4:**
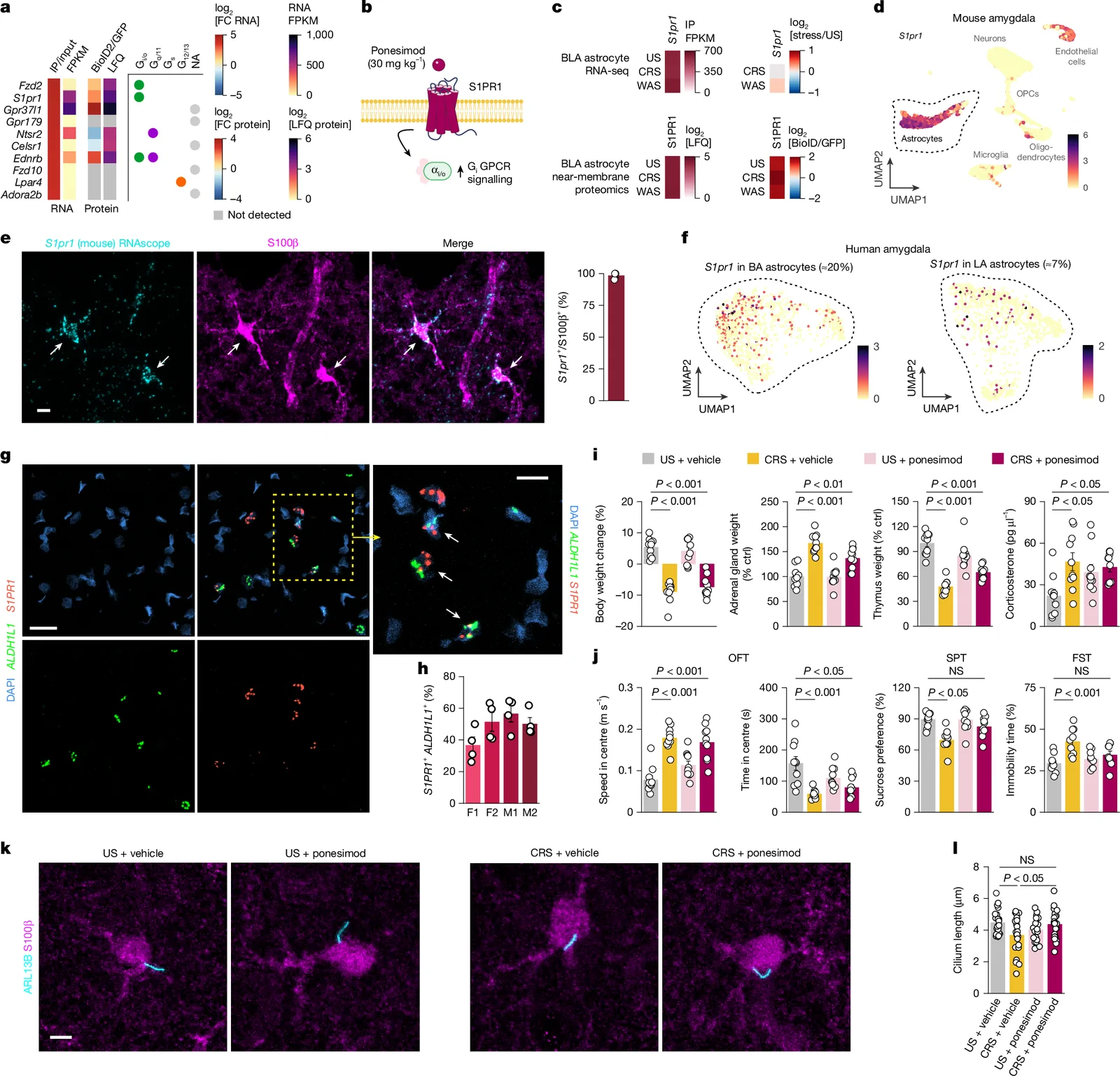
S1PR1 signalling rescues amygdala stress-induced changes. **a**, Top ten astrocyte GPCRs enriched in the amygdala from RNA-seq analyses. Membrane protein levels and GPCR coupling are shown. FC, fold change; LFQ, label-free quantification protein intensity. **b**, Schematic of the mechanism of action of the S1PR1 agonist ponesimod. **c**, *S1pr1* RNA-seq levels and astrocyte enrichment (top), and S1PR1 protein levels and near-membrane enrichment (bottom) in US, CRS and WAS mice in amygdala astrocytes. **d**, scRNA-seq analysis of *S1pr1* expression in amygdala astrocytes (re-analysis of data from ref. ^29^). OPCs, oligodendrocyte precursor cells. **e**, Combined RNAscope and IHC of *S1pr1* (cyan, RNAscope) and S100β (magenta, IHC) in mouse amygdala tissue sections (left) and quantification (right) (*n* = 3 mice). Arrows indicate *S1pr1*^+^ astrocytes. Scale bar, 5 µm. **f**, scRNA-seq analysis of *S1PR1* gene expression in human basal amygdala (BA) and lateral amygdala (LA) astrocytes (re-analysis of data from ref. ^57^). **g**, *S1PR1* (red) expression in human amygdala astrocytes (*ALDH1L1*, green) by RNAscope. Arrows indicate *S1PR1*^+^ astrocytes. Cell nuclei stained with DAPI are shown in blue. Scale bar, 25 µm (4 image panel) and 12.5 µm (zoom-in panel). **h**, Percentage of *ALDH1L1*^+^ astrocytes that express *S1PR1* in the human amygdala (*n* = 2 female (F1 and F2) and 2 male (M1 and M2) neurotypical individuals). **i**, Physiological metrics with ponesimod (pon) treatment. *n* = 10 mice per group. *P* values versus US + vehicle (veh): body weight CRS + veh *P* = 4.51 × 10^−4^; CRS + pon *P* = 6.70 × 10^−4^; adrenal gland weight CRS + veh *P* < 1 × 10^−4^; CRS + pon *P* = 1.36 × 10^−3^; thymus weight CRS + veh, CRS + pon *P* < 1 × 10^−4^; corticosterone CRS + veh *P* = 1.24 × 10^−2^, CRS + pon *P* = 2.96 × 10^−2^. **j**, Behavioural metrics. *n* = 10 mice per group. *P* values versus US + veh: OFT (speed) CRS + veh *P* < 1 × 10^−4^, CRS + pon *P* = 6.69 × 10^−4^; OFT (time) CRS + veh *P* < 1 × 10^−4^, CRS + pon *P* = 1.17 × 10^−3^; SPT CRS + veh *P* = 2.10 × 10^−4^, CRS + pon *P* = 3.7 × 10^−1^; FST CRS + veh *P* = 8.39 × 10^−4^, CRS + pon *P* = 3.51 × 10^−1^. **k**, Representative images of immunostained BLA astrocytes (S100β, magenta) and their cilium (ARL13B, cyan) from US and CRS mice treated with vehicle or ponesimod (30 mg kg^–1^; *n* = 4 mice per group). Scale bar, 5 µm. **l**, Quantification of the effect of CRS and ponesimod on the length of the primary cilium in BLA astrocytes (*n* = 21–23 cells from 4 mice per group). Bars represent the mean and error bars represent the s.e.m. Statistical tests and values are reported in Supplementary Table 1. Icons in **b** created in BioRender; Gutierrez Pelaz, S. https://biorender.com/evoeh1p (2026). Source data

**Extended Data Fig. 13:**
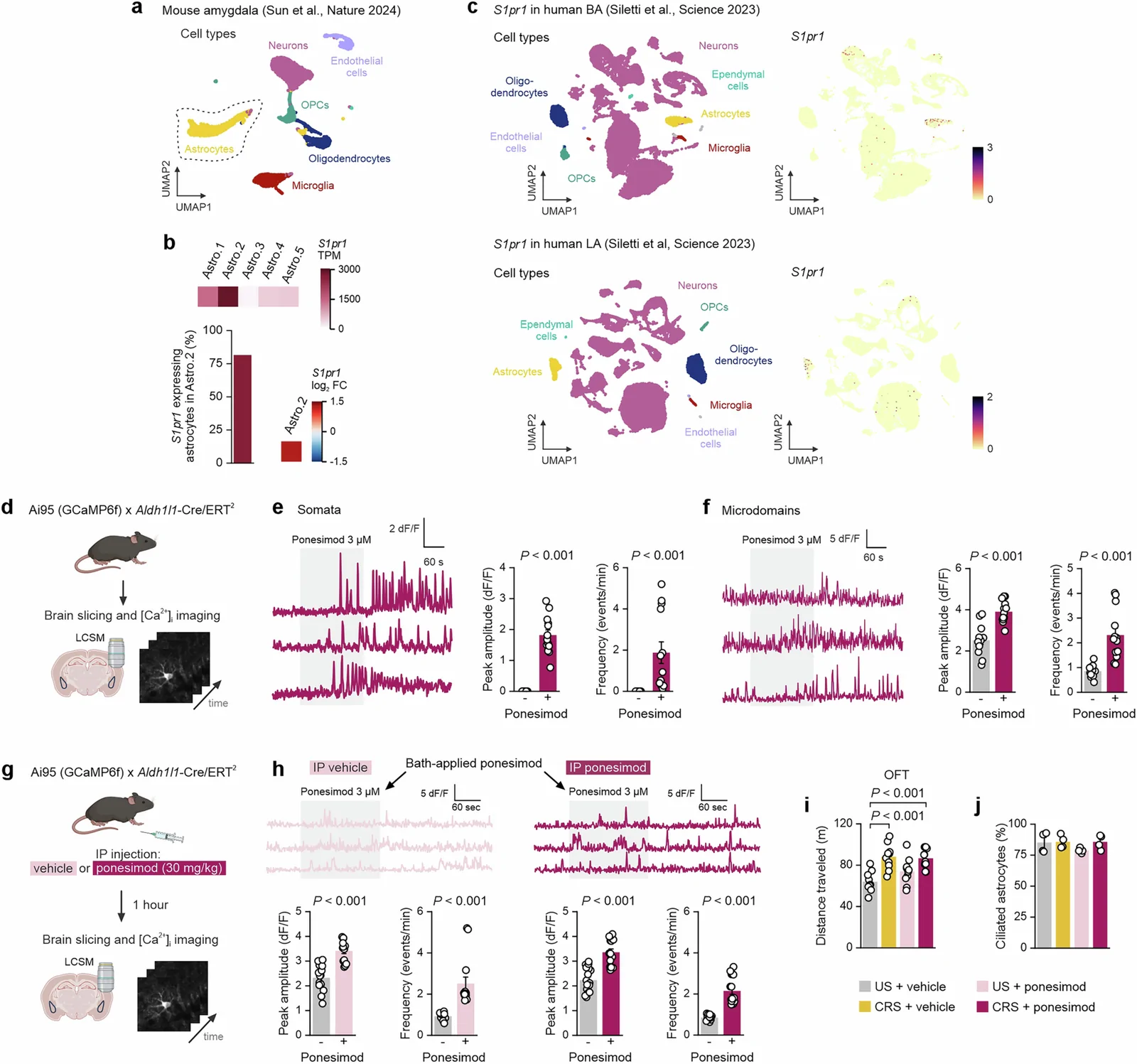
Additional S1pr1-related data. **a**, UMAP showing mouse amygdala scRNAseq from Sun et al.^29^ colored by cell type. **b**, Expression of *S1pr1* in mouse scRNAseq BLA astrocyte data from Sun et al.^29^. **c**, *S1PR1* scRNAseq gene expression in human basal amygdala (BA) and lateral amygdala (LA) astrocytes (reanalysis of data from ref. ^57^). **d**, Cartoon illustrating experimental strategy to image astrocyte calcium signaling in brain slices. **e, f**, Amygdala astrocyte Ca^2+^ signals evoked by ponesimod in the somata (e) and the microdomains (f) (n = 13 astrocytes from 6 mice; *P* values e and f: amplitude, frequency *P* < 1 × 10^−4^). **g**, Cartoon illustrating experimental strategy to image astrocyte calcium signaling in tissue slices after mice were injected intraperitoneally with ponesimod (IP injection) to assess whether repeated ponesimod treatment still induced agonism. **h**, Amygdala astrocyte microdomain Ca^2+^ signals evoked by bath-applied ponesimod in vehicle and ponesimod IP-injected mice (n = 14-17 astrocytes from 3 mice per group; *P* value all panels: *P* < 1 × 10^−4^). The ‘−’ symbol indicates before and the ‘+’ symbol indicates following ponesimod 3 µM bath application. **i**, Proportion of ciliated astrocytes in the behavioral ponesimod experiment (n = 4 mice per group; *P* values v US + veh: CRS + veh *P* = 1.19 × 10^−4^, CRS + pon *P* = 2.95 × 10^−4^). **j**, Distance traveled in the OFT (n = 10 mice per group). Bars represent the mean and error bars represent the s.e.m. Statistical tests and values are reported in Supplementary Table 1. Icons in **d** and **g** created in BioRender; Gutierrez Pelaz, S. https://biorender.com/evoeh1p (2026). Source data

**Extended Data Fig. 14:**
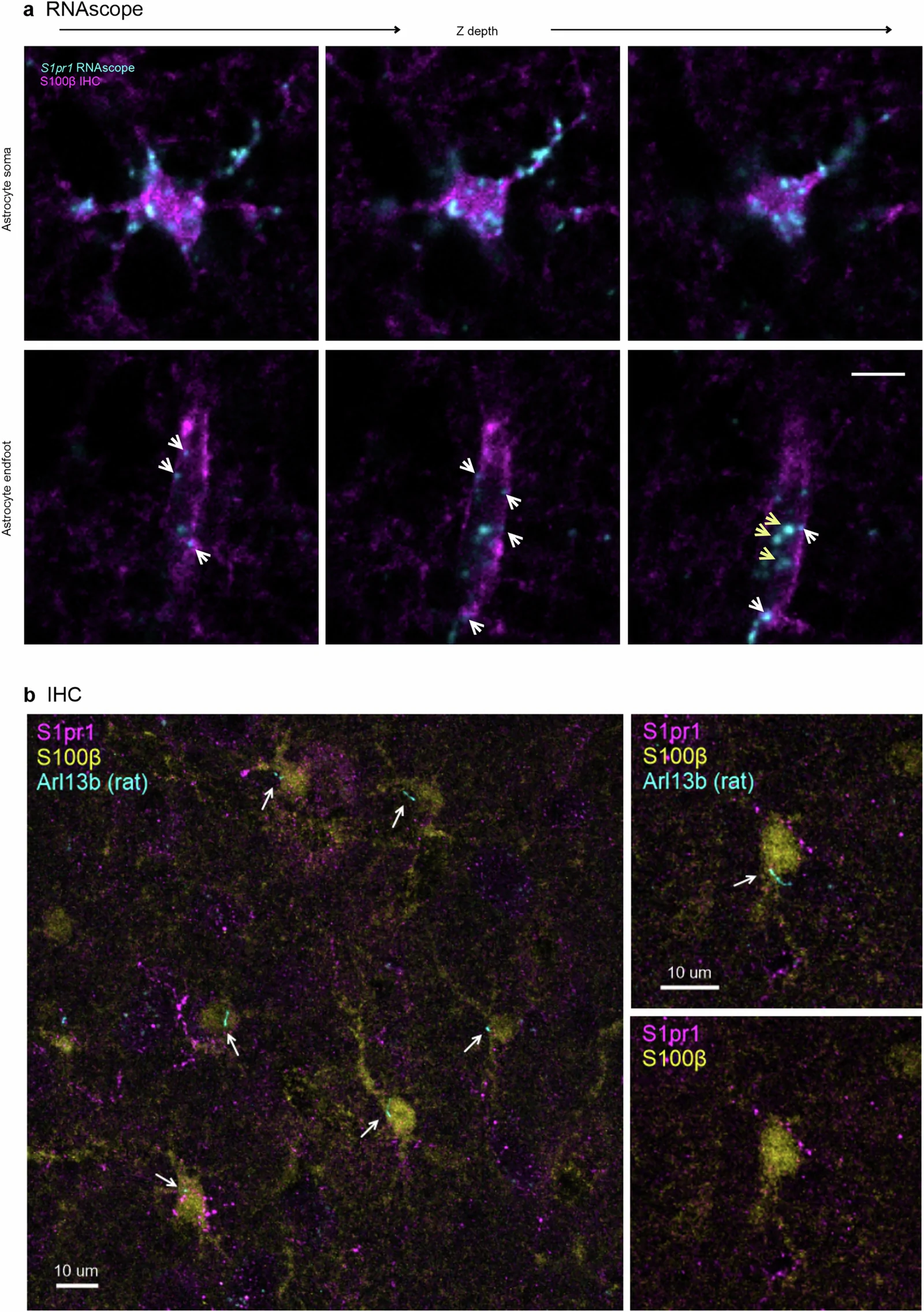
S1pr1 gene and protein expression. **a.** Single Z-stack images taken 1 µm apart from Fig. 4e. *S1pr1* expression was detected by RNAscope (cyan) and S100β was detected by IHC (magenta) (n = 3 mice). An astrocyte soma (top) and endfoot (bottom) are shown. Note that *S1pr1* colocalized with S100 β in the end foot as indicated by the white arrows, and some non-colocalized *S1pr1* was detected too (yellow arrows), which suggests some expression within endothelial cells. Scale bar: 5 µm. **b.** S1pr1 IHC. S1pr1 expression was observed for S100β positive astrocytes but was not within Arl13b positive primary cilia (n = 4 mice). Scale bar: 10 µm.

As G_i_–GPCR activation markedly improved stress-related molecular, cellular and behavioural phenotypes (Fig. 3) and because amygdala astrocytes express GPCRs (Fig. 3a–c), we mined amygdala data from US mice (Fig. 2) to identify natively expressed GPCRs^17,60^. We identified multiple GPCRs (about 25) that were highly expressed and enriched in astrocytes (Fig. 4a). Of the top ten, three were predicted to be G_i_–GPCRs that may produce effects akin to G_i_–DREADDS (Fig. 3). We chose to explore S1PR1 (encoded by *S1pr1*, also known as *Edg1*), which was both enriched in astrocytes and exhibited the highest expression levels at the RNA and protein levels (Fig. 4a,b). Existing S1PR1 agonists and functional antagonists for this G_i_–GPCR are clinically used for multiple sclerosis^66^, and it is the only member of the sphingosine-1-phosphate receptor family expressed in astrocytes^58^. The importance of S1PR1 for astrocytes has been noted^17,60,67^. We confirmed that S1PR1 was highly and specifically expressed in astrocytes using RNA-seq and near-plasma membrane proteomics of samples from US mice (Fig. 4c). Moreover, its expression was not altered by stress at the RNA or protein level (Fig. 4c). Accordingly, S1PR1 was detected in amygdala astrocytes from a mouse single-nucleus RNA-seq (snRNA-seq) database^29^ (Fig. 4d and Extended Data Fig. 13a,b). Using RNAscope, all S100β-positive amygdala astrocytes expressed *S1pr1* transcripts (Fig. 4e; *n* = 3 mice). Expression was dense in astrocyte somata but was also found in end feet and in end foot-adjacent endothelial cells^68^ (Extended Data Fig. 14a). S1PR1 protein was not found in astrocyte primary cilia (Extended Data Fig. 14b). In accord with the mouse data, *S1PR1* was expressed in human astrocytes from snRNA-seq databases^57^ (Fig. 4f and Extended Data Fig. 13c). RNAscope experiments revealed that about 50% of *ALDH1L1*-positive amygdala astrocytes from neurotypical human individuals expressed *S1PR1* (Fig. 4g,h; *n* = 4 human donors). Furthermore, S1PR1 activation with the selective agonist ponesimod (3 μM) increased Ca^2+^ signals in astrocytes in brain slices from control mice and those treated with ponesimod ex vivo and in vivo (Extended Data Fig. 13d–h; *n* = 6 mice ex vivo, and 3 mice per group in vivo), thereby demonstrating functional expression of the receptor. On the basis of these findings, we explored whether S1PR1 activation with ponesimod (30 mg kg^–1^) improves CRS-related physiological and behavioural changes and astrocyte primary cilia that are disrupted following stress (Figs. 2 and 3). As expected, the stress-related physiological changes were unaffected by ponesimod (Fig. 4i; *n* = 10 mice per group). However, ponesimod significantly improved performance in SPTs and FSTs across groups to levels equivalent to US + vehicle controls (Fig. 4j and Extended Data Fig. 13i; *n* = 10 mice per group). Furthermore, the reduction in astrocyte primary cilium length observed following CRS was recovered after ponesimod treatment (Fig. 4k,l and Extended Data Fig. 13j; *n* = 4 mice per group). Therefore, targeting native astrocyte S1PR1 G_i_–GPCRs (Fig. 4a–g) produced similar restorative effects to those observed with astrocyte G_i_–GPCR chemogenetics (Fig. 3).

## Molecular properties of amygdala astrocytes

**Fig. 5:**
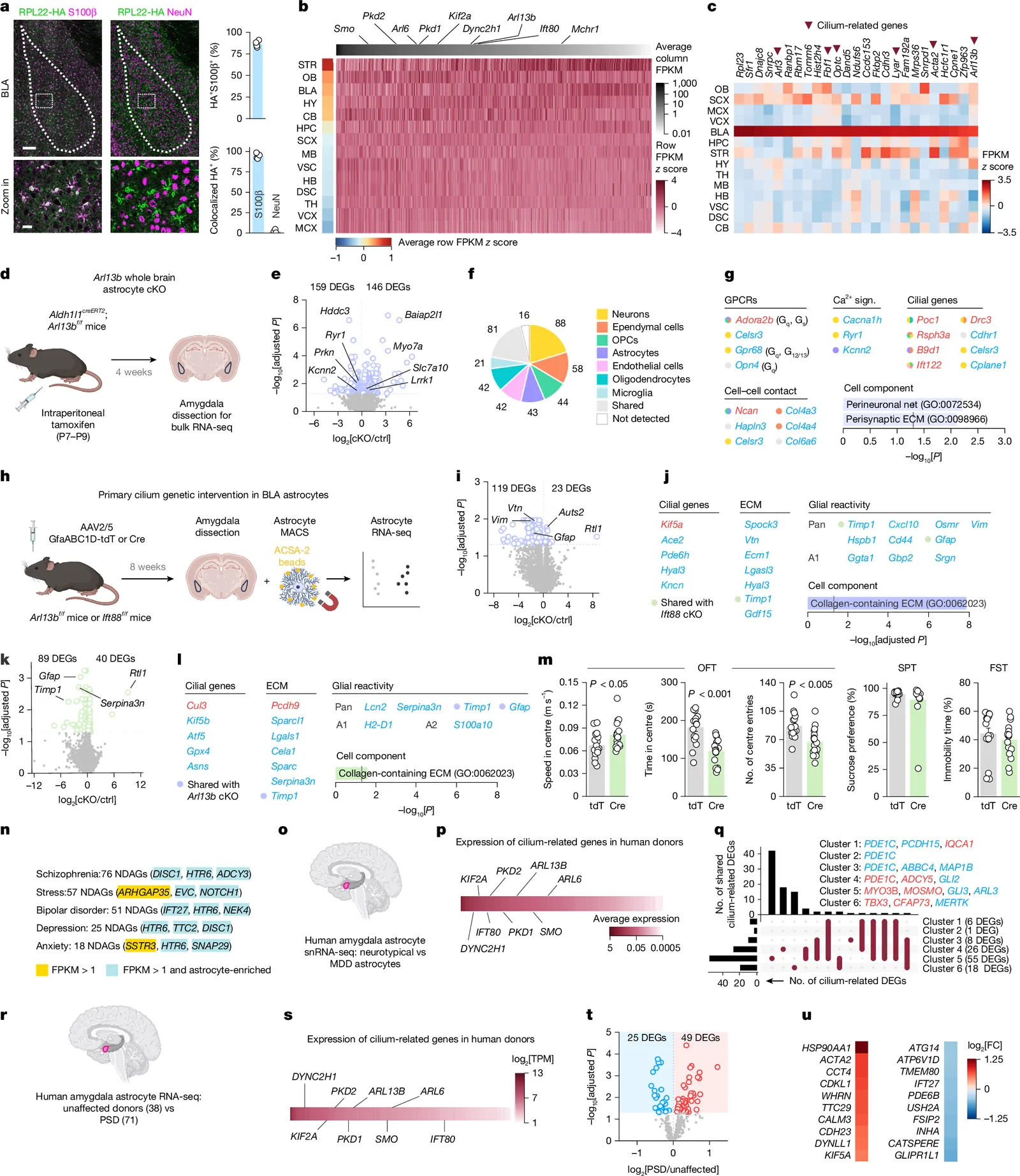
Molecular mechanisms of amygdala astrocyte primary cilia in relation to stress-induced behaviour. **a**, Left, IHC images of amygdalae (dashed outlines) from *Aldh1l1*^*cre/ERT2*^;RiboTag mice. RPL22–HA (green), astrocytes (S100β, magenta, left), neurons (NeuN, magenta, right) and nuclei (DAPI, white) are shown. Right, bar plots show astrocyte penetrance and specificity of RPL22–HA expression (*n* = 5 mice). Scale bar, 200 µm (top), 40 µm (bottom). **b**, Primary cilium-related genes (770) and their expression in astrocytes across 14 regions. CB, cerebellum; DSC, dorsal spinal cord; HB, hind brain; HPC, hippocampus; HY, hypothalamus; MB, midbrain; MCX, motor cortex; OB, olfactory bulb; SCX, sensory cortex; STR, striatum; TH, thalamus; VCX, visual cortex; VSC, ventral spinal cord. **c**, Top 25 astrocyte-enriched genes specific to the amygdala compared with the other 13 brain regions. log_2_[IP/input] > 1; adjusted *P* [IP/input] < 0.05; IP FPKM > 1 versus 13 other regions ranked by FPKM *z* score. **d**, Schematic of the experiment to conditionally and cell-selectively delete *Arl13b* in astrocytes from the entire brain. **e**, Volcano plot of control versus *Arl13b* deletion bulk RNA-seq data in mouse BLA. **f**, Mapping of bulk RNA-seq DEGs onto cell types based on scRNA-seq data from ref. ^29^. **g**, Key upregulated (red) and downregulated (blue) genes and pathways from bulk RNA-seq data shown in **e**. **h**, Schematic of the experiment to delete *Arl13b* or *Ift88* only in BLA astrocytes using AAVs to perform RNA-seq via MACS. **i**, Volcano plot of control versus *Arl13b* deletion RNA-seq data of BLA astrocytes. **j**, Key upregulated (red) and downregulated (blue) genes and pathways in astrocytes following AAV-mediated BLA astrocyte deletion of *Arl13b* shown in **i**. **k**, Volcano plot of control versus *Ift88* deletion RNA-seq data of BLA astrocytes. **l**, Key upregulated (red) and downregulated (blue) genes and pathways in astrocytes following AAV-mediated BLA astrocyte deletion of *Ift88* shown in **k**. **m**, Behavioural metrics of OFTs, SPTs and FSTs for control and AAV-mediated BLA astrocyte deletion of *Ift88*. *n* = 17 mice per group. *P *values versus tdT: OFT (speed) *P* = 2.66 × 10^−2^; OFT (time) *P* < 1 × 10^−4^; OFT entries *P* = 1.02 × 10^−3^; SPT *P* = 2.49 × 10^−1^; and FST *P* = 2.28 × 10^−1^. **n**, Cilium-related genes associated with neuropsychiatric disorders in Phenopedia. **o**, Human amygdala (pink area in the cartoon) astrocyte data of MDD^38^ assessed for expression of cilium-related genes. **p**, A total of 682 of the 770 mouse cilium-related genes were detected in human amygdala astrocytes from the MDD study. **q**, UpSet plot showing the number of cilium-related genes that were DEGs in human amygdala astrocytes. Red, upregulated; blue, downregulated. **r**, New human amygdala astrocyte (pink area in the cartoon) snRNA-seq dataset we report from donors with PSD (including bipolar disorder and schizophrenia). **s**, A total of 457 of the 770 mouse cilium-related genes were detected in human amygdala astrocytes from the PSD cohort. **t**, Volcano plot showing changes in gene expression in cilium-related genes from the PSD cohort. DEGs were obtained using edgeR. **u**, Top upregulated (red) and downregulated (blue) cilium-related genes in the PSD cohort. Bars represent the mean, and error bars represent the s.e.m. For **e**, **g**, **h**, **j**, **k** and **l**, DEGs were obtained using limma-voom and pathways were obtained using Enrichr (Fisher’s exact test followed by Benjamini–Hochberg correction). Statistical tests and values are reported in Supplementary Table 1. Icons in **d**,** h**,** o** and **r** created in BioRender; Gutierrez Pelaz, S. https://biorender.com/evoeh1p (2026). Source data

**Extended Data Fig. 15:**
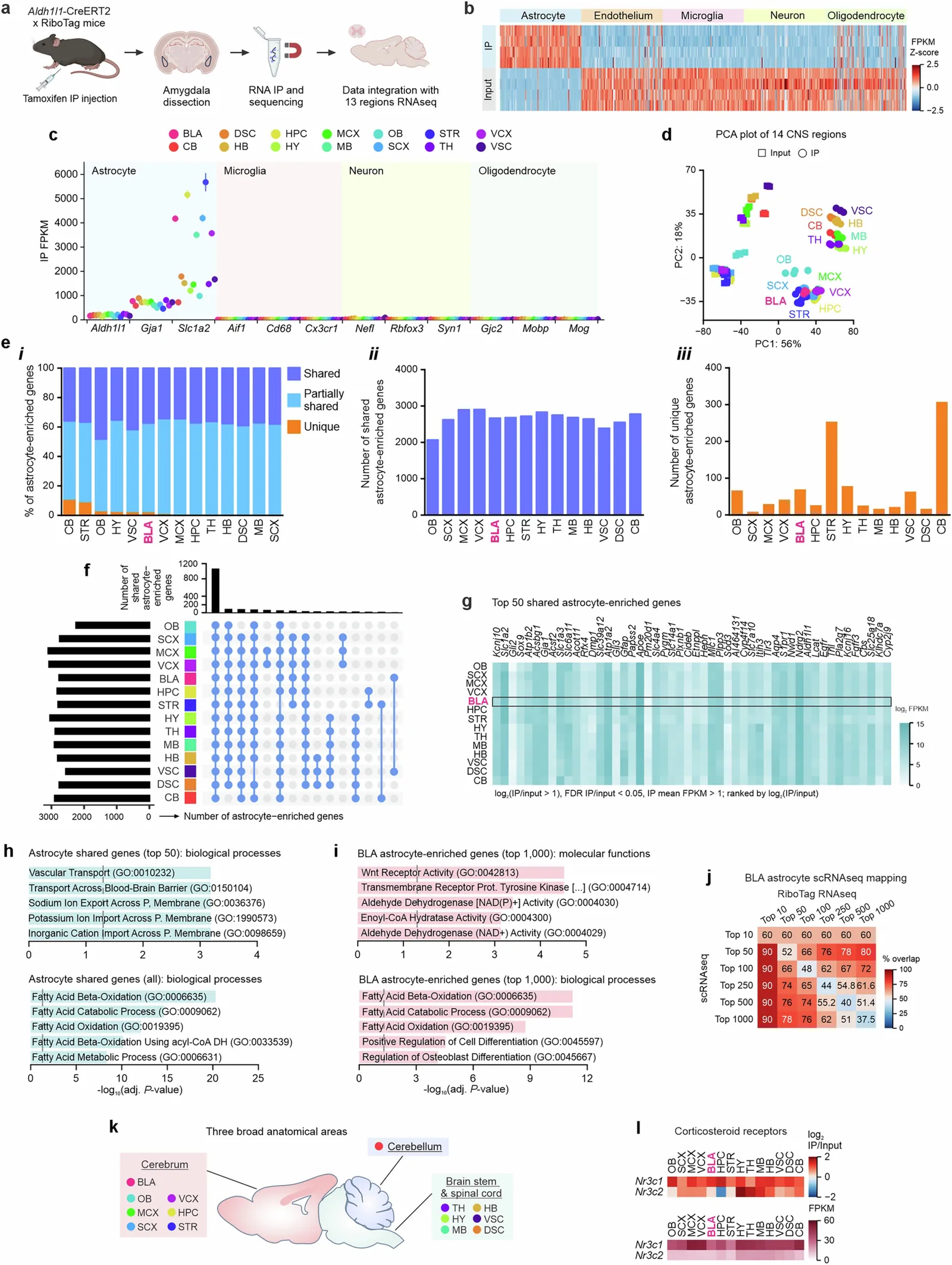
Amygdala astrocyte transcriptome analysis. **a**, Cartoon illustrating the experimental strategy for amygdala astrocyte RNAseq. **b**, Expression of selected cell-type enriched genes in the IP samples. **c**, Expression of a panel of cell-type enriched genes^105^. **d**, Principal component analysis (PCA) plot of the top 1,000 most variable genes in IP and input samples from 14 regions of the CNS. **e**, (*i*) Percentage of shared, partially shared and unique astrocyte-enriched genes among 14 brain regions. (*ii*) Number of shared astrocyte-enriched genes among 14 brain regions. (*iii*) Number of unique astrocyte-enriched genes among 14 brain regions. **f**, UpSet plot of shared astrocyte-enriched genes in 14 regions. **g**, Top 50 shared genes across 14 CNS regions. **h**, Biological processes (obtained with Enrichr, Fisher’s exact test followed by Benjamini-Hochberg) of the top 50 and of all astrocyte-shared genes. **i**, Molecular functions and biological processes (obtained with Enrichr, Fisher’s exact test followed by Benjamini-Hochberg) of the top 1,000 BLA astrocyte-enriched genes. **j**, Benchmarking of amygdala RNAseq data against recently published amygdala single-cell RNAseq^29^. **k**, Schematic of anatomical grouping of astrocytes in 14 CNS regions based on RNAseq. This figure should be used in conjunction with main Fig. 5 and the related text. **l**, Astrocyte enrichment and expression levels for the glucocorticoid (*Nr3c1*) and mineralocorticoid receptors (*Nr3c2*) in 14 CNS regions. Icons in **a** and **k** created in BioRender; Gutierrez Pelaz, S. https://biorender.com/evoeh1p (2026). Source data

**Extended Data Fig. 16:**
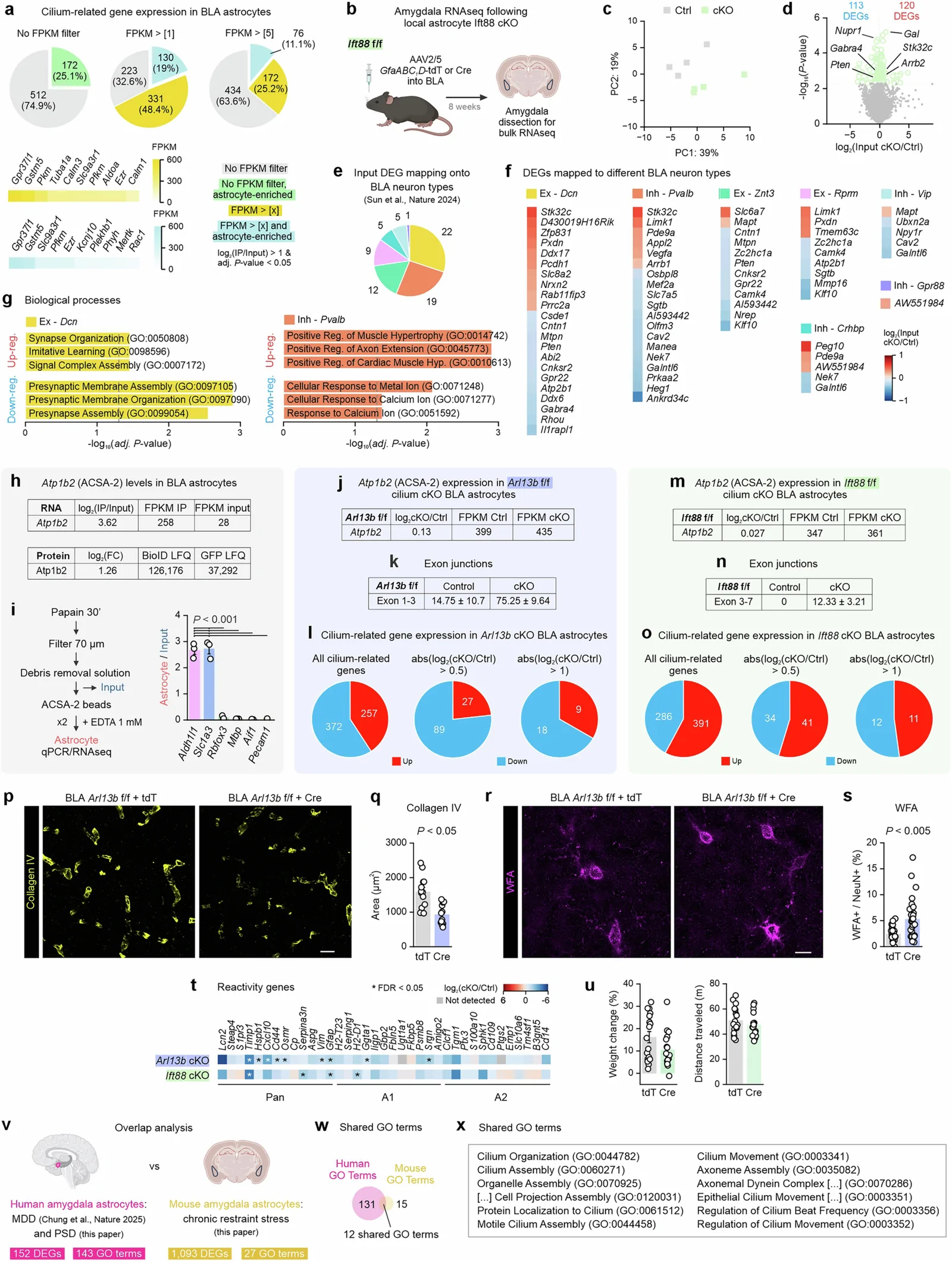
Additional primary cilium analysis. **a**, Pie charts report the numbers of primary cilium-related genes (from a list of 770) that were expressed in mouse amygdala astrocytes using the cut-off criteria shown. **b**, Cartoon illustrating the strategy to delete Ift88 only in BLA astrocytes and perform RNAseq on BLA tissue. **c**, PCA of Control and cKO samples. **d**, Volcano plot highlighting the number of up and down-regulated DEGs and some examples. DEGs were obtained with limma-voom. **e**, Mapping of bulk RNAseq DEGs onto BLA neuron types based on scRNAseq data from Sun et al.^29^. **f**, Heatmaps of DEGs mapped to each BLA neuron type. **g**, Up and down-regulated biological pathways (obtained with Enrichr, Fisher’s exact test followed by Benjamini-Hochberg) in Excitatory - *Dcn* and Inhibitory – *Pvalb* neurons. The up-regulated pathways of *Pvalb* neurons related to muscle arise from genes such as *Arrb1* (beta-arrestin-1, involved in downstream GPCR signaling) and *Pde9a* (Phosphodiesterase 9 A, involved in cGMP breakdown) and reflect GPCR biology. **h**, Gene expression and protein levels of Atp1b2 (ACSA-2) in BLA astrocytes. **i**, Summary of MACS protocol to isolate astrocytes from microdissected amygdalae (left) and validation of astrocyte enrichment relative to input by RT-qPCR (n = 3 mice). **j**, Gene expression of *Atp1b2* (ACSA-2) in BLA astrocytes from *Arl13b* f/f mice that received tdT (Ctrl) or Cre (cKO). **k**, Exon junctions in *Arl13b* f/f BLA astrocytes in control (tdT) and cKO (Cre) samples. Higher exon junction levels indicate successful removal of the *loxP*-targeted exon (in this case, exon 2). **l**, Expression of cilium-related genes in *Arl13b* cKO BLA astrocytes according to different thresholds as indicated. **m, n, o**, Same as panels j, k, l, respectively, for *Ift88*^*f/f*^-based experiments instead of *Arl13b* f/f. The *loxP*-targeted exons in *Ift88*^*f/f*^ mice are exons 4-6. **p, q**, Representative images and quantification of collagen IV staining by IHC in mouse amygdala tissue from *Arl13b* f/f mice that received tdT or Cre in the BLA (# fields of view = 13 tdT, 12 Cre, 3 mice per group; *P* = 3.07 × 10^−4^). Scale bar: 20 µm. **r, s**, Representative images and quantification of WFA staining by IHC in mouse amygdala tissue from *Arl13b* f/f mice that received tdT or Cre in the BLA (# fields of view = 23 tdT, 29 Cre, 3 mice per group; *P* = 4.41 × 10^−3^). Scale bar: 20 µm. **t**, Heatmaps showing widespread down-regulation of glial reactivity genes^103^ in BLA astrocytes from *Arl13b* and *Ift88* cKO mice. **u**, Weight change since surgery and distance traveled of BLA astrocyte-*Ift88* cKO mice (n = 17 mice per group). **v**, Cartoon illustrating the analysis of overlap between human and mouse DEGs. **w**, Shared GO terms of human and mouse experiments. **x**, List of shared GO pathways from panel w. Bars represent the mean and error bars represent the s.e.m. Statistical tests and values are reported in Supplementary Table 1. Icons in **b** and **v** created in BioRender; Gutierrez Pelaz, S. https://biorender.com/evoeh1p (2026). Source data

Relatively little is known about amygdala astrocytes, which prompted us to use astrocyte-selective *Aldh1l1*^*cre/ERT2*^;RiboTag mice to evaluate their molecular properties in relation to other CNS regions^58^ (Fig. 5a, Extended Data Fig. 15a and Supplementary Table 6; *n* = 4 mice per sample, 4 samples per region). Multidimensional scaling showed separation between major brain areas, and the astrocyte-specific immunoprecipitation (IP) fraction contained known astrocyte markers (Extended Data Fig. 15b–d). We identified the top 50 astrocyte enriched genes shared across CNS regions, including the amygdala (for example, *Kcnj10*, *Sox9*, *Gja1*, *Slc1a2* and *Apoe*), representing core features of amygdala astrocytes^58^ (Extended Data Fig. 15e–h). Amygdala astrocytes also showed 2,669 astrocyte-enriched genes relative to input (log_2_[IP/input] > 1, adjusted *P* < 0.05, IP fragments per kilobase million (FPKM) > 1) and 495 amygdala-unique DEGs relative to other CNS areas (log_2_[IP/average IP of other regions] > 1, adjusted *P* < 0.05, IP FPKM > 1). Furthermore, 69 genes were enriched in astrocytes and unique to the amygdala (log_2_[IP/input] > 1, adjusted *P* < 0.05, IP FPKM > 1), which enabled us to impute amygdala-enriched functions (Extended Data Fig. 15e,i). We also referenced amygdala astrocyte-enriched genes against mouse amygdala astrocyte scRNA-seq data^29^ and found about 90% overlap (Extended Data Fig. 15j). Overall, amygdala astrocytes clustered with those from the hippocampus, striatum and cortex (Extended Data Fig. 15d,k) and had shared and amygdala-specific features. For example, the corticosteroid receptors *Nr3c1* and *Nr3c2* were enriched in astrocytes for most regions of the CNS without dominance in the amygdala (Extended Data Fig. 15l). However, consistent with our findings related to the primary cilium (Figs. 1–4), amygdala astrocyte expression of cilium-related genes^5^ was higher than most of the other regions (Fig. 5b,c and Extended Data Fig. 16a), which suggests that astrocyte primary cilium biology may be region-specific. This finding is consistent with the field of astrocyte diversity and the functions of astrocyte subtypes. In this regard, the molecular profiles we report here enable mechanistic study of amygdala astrocyte responses in vivo^25,31^.

## Effects of astrocyte primary cilia disruption

As astrocytes regulate neurons and other cells, we deleted *Arl13b*^5^ in brain-wide astrocytes and performed bulk RNA-seq of the amygdala to shed light on how gene expression of amygdala cells was altered (Fig. 5d–g and Supplementary Table 7). This genetic disruption of astrocyte primary cilia resulted in 146 upregulated and 159 downregulated DEGs (Fig. 5e), which, on the basis of snRNA-seq^29^, we mapped to neurons, astrocytes and other cells (Fig. 5f). These tissue-level changes underscore important functions of primary cilia in astrocytes^5^ and consequences for neurons^5^. Key upregulated and downregulated DEGs included those related to GPCRs, Ca^2+^ signalling, primary cilia and cell–cell contacts (Fig. 5g). Next, we used our AAV strategy (Fig. 2m) to disrupt astrocyte primary cilia only in the amygdala in adult mice and performed amygdala bulk RNA-seq (Extended Data Fig. 16b–g) and astrocyte-specific RNA-seq with MACS (Fig. 5h–l and Extended Data Fig. 16h–o). *Arl13b* and *Ift88* deletion from amygdala astrocytes resulted in 142 and 129 DEGs, respectively (Fig. 5h–l). In both cases, the key pathways were glial reactivity, extracellular matrix (ECM), cilium biology and collagen containing ECM (Fig. 5j,l). We validated collagen by IHC and found that it was decreased, and we validated perineuronal nets known to be replete with ECM components by WFA staining and found a significant increase (Extended Data Fig. 16p–s); both of these changes were in agreement with the RNA-seq data (Fig. 5d–l). Furthermore, astrocyte reactivity genes were mostly downregulated in both mouse lines (Extended Data Fig. 16t), which was consistent with the decreased levels of GFAP following stress (Fig. 1k,l). Next, we performed behavioural tests on mice in which amygdala astrocyte *Ift88* was deleted and assessed with three markers (Fig. 2o) and RNA-seq (Fig. 5k,l and Extended Data Fig. 16). There were significant deficits in stress-related behaviours measured by OFTs, but not in those measured by SPTs and FSTs (Fig. 5m and Extended Data Fig. 16u; 16–17 mice per group). Overall, these data provide evidence that loss of primary cilia in amygdala astrocytes alone can alter astrocytes and surrounding cells and can lead to some stress-related behaviours. These data imply that amygdala astrocyte primary cilia signalling contributes to anxiety states, in accord with recent work^25^, whereas other stress-related behaviours (Fig. 1) require convergence of additional changes induced by CRS and WAS.

## The primary cilium in neuropsychiatric disease

Mining of the Phenopedia (v.10.0) database revealed numerous cilium-related genes associated with neuropsychiatric disorders in humans, including depression, stress, bipolar disorder and anxiety (Fig. 5n and Supplementary Table 8). Furthermore, 682 out of 770 primary cilium-related genes were expressed in human amygdala astrocytes identified from a previous study^38^ (Fig. 5o,p and Supplementary Table 9). Of the six clusters of human astrocytes^38^ defined by snRNA-seq, all of them displayed cilium-related DEGs in patients with MDD relative to unaffected individuals^38^, with astrocyte cluster 5 showing the largest number (Fig. 5q). We next performed a specific set of studies by gathering and analysing amygdala astrocyte snRNA-seq data from unaffected neurotypical donors and those with psychosis spectrum disorders (PSDs), which included donors with schizophrenia or bipolar disorder (Fig. 5r). Overall, 457 out of 770 primary cilium-related genes were expressed in human amygdala astrocytes in neurotypical humans (Fig. 5s and Supplementary Table 10), and 74 cilium-related genes were significant DEGs in astrocytes in psychosis-related disorders relative to unaffected individuals (Fig. 5t,u). Accordingly, there was marked overlap in pathways between mouse CRS DEGs and human cilium-related neuropsychiatric DEGs (Extended Data Fig. 16v–x). Differences between the human and mouse datasets probably reflect methodological and biological factors, as well as redundancy among individual cilium-related genes for common mechanisms. Along with our mouse studies (Figs. 1–5), our findings show that primary cilium biology is a feature of both mouse and human amygdala astrocytes. Moreover, cilium-related genes are disrupted in mouse astrocytes following stress (Figs. 1–5) and in human astrocytes from stress-related disorders (Fig. 5n–u). Consistent with the mouse data (Fig. 4c), *S1PR1* was not a DEG in the neuropsychiatric disorders analysed (Fig. 5n–u).

## The astrocyte primary cilium proteome

**Fig. 6:**
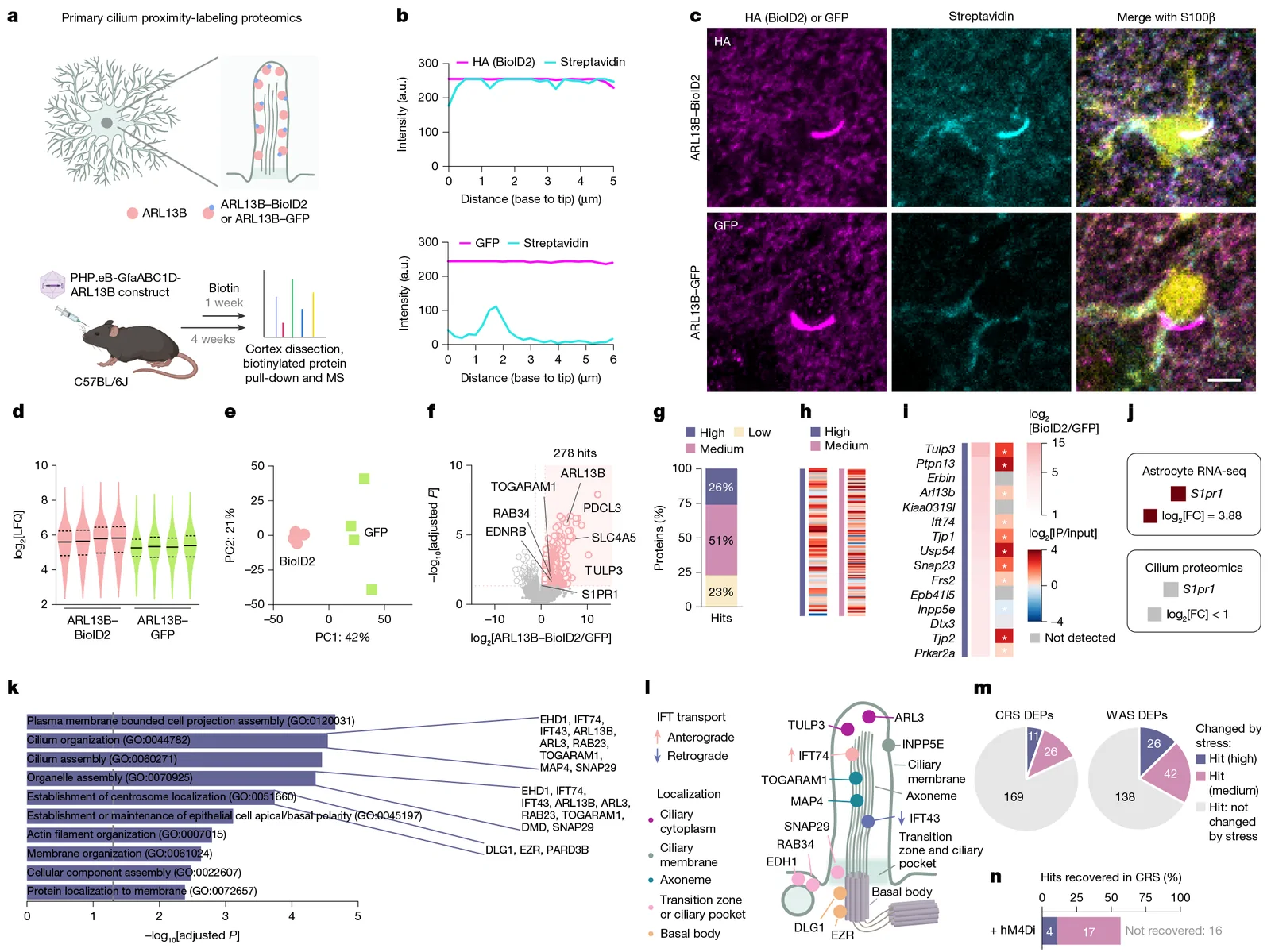
ARL13B-based proximity-dependent proteomics of astrocytic primary cilia. **a**, Schematic of the experiment for cillium proximity-labelling proteomics. The entire cortex was collected and used for streptavidin-mediated pull-down of biotinylated proteins. **b**, Line profile analysis (in arbitrary units (a.u.)) of HA and GFP labelling in the primary cilium of images shown in **c**. **c**, Representative images of HA and GFP labelling in cortex astrocytes (*n* = 3 mice per group). Note that HA and GFP (and streptavidin in the BioID2 construct only) accumulate in the primary cilium relative to outside it. Scale bar, 5 µm. **d**, Intensity of the proteins detected in each sample. Solid lines represent the median and the dotted lines represent 25th and 75th percentiles. **e**, PCA of proteomic samples. **f**, Volcano plot showing 278 hits are enriched in ARL13B–BioID2 compared with ARL13B–GFP. Hits were obtained using limma and defined by log_2_(BioID2/GFP) > 1 and adjusted *P* < 0.05, as in the rest of the paper. **g**, Hits were classified according to probability of being cilium-related in the database CilioGenics. **h**, Heatmaps showing cortex RNA-seq-based astrocyte enrichment for high and medium CilioGenics probability hits. The colour scale (red, highly enriched; blue, not highly enriched) is the same as in **i**. **i**, Top 15 high CilioGenics probability hits and their astrocyte-enrichment from **f** and **g**. Asterisks indicate hits with adjusted *P* < 0.05. **j**, *S1pr1* expression was highly enriched in cortex astrocytes (data from ref. ^58^) but was not detected as a hit in the cilium proteome. **k**, Gene ontology (GO) biological processes of the ARL13B high probability hits and example proteins (obtained using Enrichr, Fisher’s exact test followed by Benjamini–Hochberg correction). **l**, Putative localization in the primary cilium of high probability hits from **f** and **g** based on published work (Methods). **m**, Cilium high- and medium-probability proteome hits (206) that changed in the CRS and WAS near-membrane astrocyte proteome coloured by CilioGenics probability. **n**, Hits from **m** that were rescued by hM4Di activation. Icons in **a** created in BioRender; Gutierrez Pelaz, S. https://biorender.com/evoeh1p (2026).

To address the limited knowledge of the molecular composition of astrocyte primary cilia, we profiled the astrocyte primary cilium proteome using proximity-dependent biotinylation with ARL13B–BioID2, relative to ARL13B–GFP, delivered with astrocyte-selective AAVs (Fig. 6a; *n* = 4 mice per group). Owing to its small size, we could not obtain sufficient protein from the amygdala. Instead, we targeted cortical astrocytes, which clustered with amygdala astrocytes in multidimensional scaling (Extended Data Fig. 15d,k), have primary cilia (Extended Data Fig. 5) and provided larger tissue yields. Streptavidin staining revealed strong biotinylation along the primary cilia of S100β-positive astrocytes that colocalized with ARL13B–BioID2 immunoreactivity, whereas little biotinylation was observed in the ARL13B–GFP group (Fig. 6b,c). Mass spectrometry analyses identified significantly higher intensity and more proteins in the ARL13B–BioID2 group than in the GFP control, and principal component analysis (PCA) clearly separated the two conditions, which enabled detection of 278 proteins as hits in the ARL13B–BioID2 samples (log_2_[BioID2/GFP] > 1), adjusted *P* < 0.05), including known primary cilium proteins (for example, ARL13B, TOGARAM1 and TULP3; Fig. 6d–f and Supplementary Table 11). We scored these 278 proteins using CilioGenics, which revealed that 206 out of 278 (74%) were classified as high or medium probability (Fig. 6g,h). Many were also enriched in cortex astrocyte RNA-seq data (Fig. 6h, and top 15 shown in Fig. 6i). Notably, S1PR1 was absent from the 278 hits comprising this putative astrocyte primary cilium proteome (Fig. 6f,j) despite its strong detection in the astrocyte plasma membrane proteome and in RNA-seq and RNAscope analyses (Fig. 4). These findings suggest that S1PR1 exerts its beneficial effects on stress-related behaviours from the plasma membrane, where its activation is associated with increased primary cilium length (Fig. 4), rather than by localizing to the primary cilium itself. Consistent with bona fide ciliary identification, the 206 CilioGenics high and medium probability hits were enriched for biological processes related to primary cilium function (for example, cilium organization, cilium assembly, centrosome assembly and actin filament organization; Fig. 6k). In Fig. 6l, we illustrate the putative subciliary localization of selected proteins based on previous work; spatial proteomics^69^ of astrocyte primary cilia will be an important goal for future work. Among the 206 CilioGenics high and medium probability proteins, 18% and 26% were altered by CRS and WAS, respectively (Fig. 6m), and of the 37 proteins significantly disrupted by CRS, approximately 50% were restored by G_i_–GPCR activation (Fig. 6n). These data provide a putative astrocyte primary cilium proteome and bolster findings (Figs. 1–5) that stress disrupts astrocyte primary cilia and that G_i_–GPCR activation can partially restore defects.

## Summary and discussion

Elucidating the mechanisms by which stressful life events precipitate enduring behavioural changes that result in psychiatric disease remains a fundamental challenge. Astrocytes, as integral components of neural circuits, engage in developmental, structural, signalling and homeostatic interactions with neurons throughout the brain, including in the amygdala^25,29–31,38^, a region implicated in the integration of valence and the orchestration of emotional and stress responses^1,10–12^. However, astrocytic mechanisms of relevance to stress-induced behavioural changes have remained largely unexplored, although there has been progress with neuroimmune interactions and psychedelics^38^.

In this study, we identified an important role for amygdala astrocytes in the modulation of stress-related behaviours mediated via signalling pathways associated with their primary cilia^2^. Exposure to stress produced marked alterations in amygdala astrocytes at both the proteomic and transcriptomic levels, assessed using astrocyte-selective reagents. These changes reflected dysregulation of genes and proteins associated with the structure and function of the primary cilium. Consistent with this molecular signature, morphological analyses revealed a reduction in primary cilium length on astrocytes following stress, which indicates that ciliary dysfunction has a role in response to stress-induced changes in the amygdala.

Although a precise threshold for functionally meaningful changes in primary cilium length has not been established, the primary cilium is recognized as a morphologically dynamic organelle in which even modest alterations in length, either increases or decreases, can influence signalling and cellular function. In addition to altering the amount, spatial distribution or effective density of signalling components in the cilium, changes in ciliary length may influence cellular signalling through several other mechanisms^2,33^. Shortening of the primary cilium may reduce the available membrane surface for receptor localization, which may affect signal sensitivity to extracellular ligands. It may also influence intraflagellar transport dynamics, which in turn may alter the trafficking, delivery or turnover of signalling proteins in the ciliary compartment. Changes in length could also modify the spatial organization of subciliary domains (for example, base, shaft and tip)^2,33^, potentially affecting interactions among receptors and scaffolding proteins. Because the primary cilium can function as a mechanosensor, shorter cilia may also alter the mechanical properties of the organelle and its ability to transduce physical stimuli. Finally, cilium length may influence cellular states linked to ciliary dynamics, such as quiescence, which could contribute to altered downstream signalling outcomes. These possibilities may contribute to the changes reported in this study, but we are not aware of any cases in which these mechanisms have been teased apart in vivo and are not aware of genetic tools to do so systematically. Consistent with a role for primary cilia in neurobiological responses, genetic ciliopathies frequently include neurological manifestations^2^. Together, our findings indicate that stress induces coordinated changes in cilium-associated genes, proteins and morphology in amygdala astrocytes, consistent with a role for astrocytic ciliary alterations in stress-related pathology. Further mechanistic studies are now needed to unlock the signalling and biophysics of the primary cilium.

Given the established roles of primary cilia as signalling hubs^2^, particularly for GPCR pathways, the consequential alterations of cilial genes in disease^70^ and the importance of GPCRs in astrocyte biology^7^, we explored amygdala astrocyte GPCRs and their responses after stress. Through chemogenetic manipulation or targeted modulation of endogenous S1PR1, we were able to restore primary cilium length, normalize stress-induced molecular perturbations and produce substantial improvements in stress-related behavioural phenotypes. S1PR1 expression was strongly detected in amygdala astrocytes in both mouse and human tissue, which highlights the translational potential of these findings related to S1PR1 drugs approved for use in humans^66^. The high level of S1PR1 in the plasma membrane proteome of amygdala astrocytes suggests that it exerts its actions from that location rather than from the primary cilium. However, further work on the relationship between astrocytic S1PR1 and cilia is needed. Blood sphingosine-1-phosphate levels have been reported to increase when stress-related behaviours were treated^71^, but the relevance of such clinical data will require further study. We also identified multiple other astrocyte GPCRs as potential targets, including several pharmacologically tractable orphan receptors expressed on astrocytes (for example, GPR37L1, GPR162, GPR158 and GPR179).

Genetic disruption of primary cilia through the deletion of key genes (*Arl13b* and *Ift88*) in amygdala astrocytes resulted in astrocytic and additional effects, such as perturbing the molecular properties of neighbouring parenchymal cells, including neurons. This finding highlights the multicellular regulatory capacity of astrocytes in the amygdala microenvironment and suggests that astrocytic cilia serve as modulators of local circuit functions during stress. Primary cilia are observed in most astrocytes^35^ and have developmental roles^5^; thus, we found that in humans, cilia-related genes were abundant in amygdala astrocytes and were altered in several psychiatric disorders. Although studies of how astrocyte cilia influence neural circuits, to our knowledge, do not yet exist, our findings and tools now enable such circuit-level dissection.

In summary, our findings establish that stress disrupts primary cilia in amygdala astrocytes and that GPCR-mediated restoration of cilium integrity can reverse several molecular and behavioural sequelae of stress. These results advance our understanding of the fundamental mechanisms of glial cells that underlie stress adaptation in vertebrates. By defining a link between astrocyte primary cilia dysfunction and stress, our work opens new avenues for the development of targeted strategies to ameliorate maladaptive behavioural responses due to stress. As stress and depression-related phenotypes are common in neurodegenerative diseases, future studies could determine whether primary cilium mechanisms contribute to neurological disease.

## Methods

### Mouse models

All animal experiments at the University of California Los Angeles (UCLA) were conducted in accordance with the US National Institutes of Health (NIH) Guide for the Care and Use of Laboratory Animals and were approved by the Chancellor’s Animal Research Committee at UCLA. The UCLA ARC protocol numbers for the experiments were 2009-043, 2020-002, 2017-039 and 2022-016. All mice were housed with food and water available ad libitum in a 12-h light–dark environment at temperatures of 20–22 °C with 40–60% humidity. All mice were 2–5 months old when used for experiments, were healthy with no obvious behavioural phenotype, were not involved in previous studies and were euthanized during the light cycle. Wild-type C57BL/6N mice were maintained in an in-house breeding colony or purchased from The Jackson Laboratory (JAX) or Taconic Biosciences. Approximately equal numbers of male and female mice were used and no differences were observed between sexes. RiboTag mice (B6N.129-Rpl22tm1.1Psam/J, JAX stock 011029) were acquired from JAX and crossed with *Aldh1l1*^*cre/ERT2*^ BAC mice (B6N.FVB-Tg(Aldh1l1-Cre/ERT2)1Khakh/J, JAX stock 029655) fully backcrossed to C57BL/6NTac background in an in-house breeding colony. GCaMP6f mice (Ai95D (C57BL/6J), JAX stock 028865) were acquired from JAX and crossed with *Aldh1l1*^*cre/ERT2*^ BAC mice (B6N.FVB-Tg(Aldh1l1-Cre/ERT2)1Khakh/J, JAX stock 029655) in an in-house breeding colony. Ponesimod behavioural experiments were performed at ONO Pharmaceutical and were approved by the Institutional Animal Care and Use Committee. *Arl13b*^*f/f*^;*Aldh1l1*^*cre/ERT2*^ mice were group housed (4–5 mice of the same sex per cage) in a controlled environment (12-h light–12-h dark cycle at 21 °C) with unrestricted access to water and a standard chow diet at the University of Calgary, and experiments were approved by the University of Calgary Health Sciences Animal Care Committee, Health Sciences Animal Care Committee, and all experiments were completed in accordance with the Canadian Council of Animal Care guidelines. *Arl13b*^*f/f*^ and *Ift88*^*f/f*^ mice^72,73^ were a gift from J. Guo and were maintained in an in-house breeding colony at UCLA. All mice were assigned to experimental groups at random. Because of the experimental design and availability of resources, blinding was not always feasible. For behavioral experiments, we minimized subjectivity as much as possible by using automated analysis software whenever possible.

### Ethics statement

Human postmortem brain tissue samples were obtained from the Harvard Brain Tissue Resource Center, a NIH NeuroBioBank repository. All procedures were approved by the Mass General Brigham Institutional Review Board and conducted in accordance with institutional guidelines, NIH regulations and the Health Insurance Portability and Accountability Act. Written informed consent for brain donation was obtained from the legal next-of-kin or legally authorized representative. All samples were de-identified before distribution.

### Tamoxifen injections for inducible gene expression

To selectively express RPL22–HA in astrocytes, hemizygous *Aldh1l1*^*cre/ERT2*^;RiboTag mice^74,75^ were injected with 75 mg kg^–1^ tamoxifen (Sigma-Aldrich, T5648) dissolved in corn oil once per day for 5 consecutive days at 6–8 weeks of age and were euthanized 2–3 weeks after tamoxifen injections for astrocyte RNA-seq experiments. *Arl13b*^*f/f*^;*Aldh1l1*^*cre/ERT2*^ mice were administered tamoxifen (200 μg per mouse per day) from postnatal days 7–9 and were euthanized 4 weeks later for RNA-seq experiments.

### CRS

Mice were weighed and placed in restrainers (diameter of 2.5 cm × 9.5 cm, 551-BSRR, Plas-Labs) for 6 h each day (from 10:00 to 16:00) for 14 consecutive days. US mice were weighed and kept in their home cages. Light intensity during restraint stress was maintained at approximately 50 lux. All mice were returned to their home cages at the end of the stress period.

### WAS

Mice were weighed and placed on a platform (diameter of 5 cm × 10 cm) attached to the bottom of a plastic tank (45-cm long × 25-cm wide × 25-cm high) for 2 h each day (from 10:00 to 12:00) for 14 consecutive days. The plastic tank was filled with water (25 °C) up to 1 cm below the top of the platform. US mice were weighed and kept in their home cages. Light intensity during WAS was maintained at approximately 50 lux. All mice were returned to their home cages at the end of the stress period.

### SNI model

All surgical procedures were conducted under general anaesthesia using continuous isoflurane (induction at 5%, maintenance at 1–2% v/v). The depth of anaesthesia was monitored continuously and adjusted as necessary. After the induction of anaesthesia, mice were administered with 0.1 mg kg^–1^ buprenorphine (Buprenex) subcutaneously before surgery. The surgical incision site was then cleaned 3 times with 10% povidone iodine and 70% ethanol (v/v). The skin and muscles were incised to expose the sciatic nerve. The common peroneal nerve and tibial nerve, two of the three terminal branches of the sciatic nerve, were tightly ligated with 6-0 silk sutures and then cut distally, whereas the sural nerve was left intact. The muscles and skin were then sutured with 4-0 silk sutures. In the sham surgery, only the sciatic nerve was exposed, and afterwards, the muscles and skin were sutured. After surgery, mice were allowed to recover overnight in cages placed partially on a low-voltage heating pad. Buprenorphine was administered twice a day for up to 2 days after surgery. The mice were allowed to recover for 2 weeks after SNI before performing physiological and behavioural evaluations comprising OFTs, conditioned-thermal place-aversion tests and Von Frey tests. As expected^76^, SNI resulted in the development of mechanical and thermal allodynia, as well as hyperalgesia, in the area innervated by the sural nerve^76^.

### OFT

The open-field chamber consisted of a square arena (40 cm × 40 cm) enclosed by plastic walls (30 cm in height). The centre of the arena was defined as the area 10 cm away from the chamber walls. The light intensity in the centre of the open field was maintained at 50 lux. The locomotor activity of the mice was recorded for 15 min using a camera located above the open-field chamber. The time spent, centre entries and speed in the centre, as well as the total distance travelled, were analysed using the automated video tracking software ANY-maze (v.6.3, Stoelting).

### SPT

The mice were switched to individual housing and given two water bottles to acclimate them to the bottles. On day 2, the mice were given two bottles: one containing water and the other containing 1% sucrose. On day 3, the mice were fasted and again given two bottles, one with water and the other with 1% sucrose, both of which were pre-weighed. The mice had access to these bottles for 16 h (from 18:00 to 10:00). After the test period, the weight of each bottle was measured, and sucrose preference (%) was calculated using the following formula: (1% sucrose intake/(water intake + 1% sucrose intake)) × 100. The mice were euthanized after the SPTs.

### FST

Mice were placed in a cylinder (diameter of 12 cm ×30 cm in height) filled with water to a depth of 20 cm, and their swimming behaviour was recorded with a video camera for 6 min. The light intensity of the test area was maintained at 50 lux. The time the mice remained immobile was recorded.

### Von Frey test

The pain threshold was measured on day 21 after SNI surgery using von Frey filaments. The following 7 different strengths of von Frey filaments were used: 0.04, 0.07, 0.16, 0.4, 0.6, 1 and 2 g. Mice were placed in individual small cages on a wire mesh floor and allowed to acclimate for up to 1 h. The von Frey filaments were applied vertically to the plantar surface of the hind paw on the SNI-treated side from underneath the wire mesh floor. A quick withdrawal response or paw flicking was considered a positive pain response, and pain response was measured using a previously described up–down method^77^.

### Conditioned-place temperature-aversion test

The conditioned-place temperature-aversion test was conducted using an apparatus (33 × 16.5 ×30 cm, Thermal Place Preference-2 (BIO-T2CT, Bioseb)) equipped with two plates for which temperatures can be individually controlled and enclosed by Plexiglass. On the first day, mice were placed in the centre of the apparatus with both plates set to 25 °C, and the mice were allowed to freely explore the plates for 10 min to acclimate to the apparatus. Subsequently, the test plate was set to 19 °C whereas the reference plate remained at 25 °C, and visual cues were placed on the outside of the apparatus on the test plate side. The mice were placed in the centre of the apparatus and allowed to freely explore the plates for 10 min during two conditioning trials, with an interval of approximately 3 h between trials. On the second day, both the test plate and the reference plate were set to 25 °C, with visual cues still placed on the outside of the apparatus on the test plate side. The mice were placed in the centre of the apparatus and allowed to explore the plates freely for 10 min. The locomotor activity of the mice was recorded using a camera located above the apparatus, and the time spent on each plate was automatically analysed using Bioseb software. Place preference (%) was calculated using the following formula: (time spent on test plate/(time spent on test plate + time spent on reference plate)) × 100.

### Blood and tissue collection

After anaesthetizing the mice with isoflurane, and following euthanasia, an incision was made in the abdomen to collect 0.5 ml blood from the abdominal vena cava, which was then placed into a 1.5 ml tube containing heparin. Subsequently, the adrenal glands from both sides and the thymus were excised and their wet weights were measured. The collected blood was centrifuged at 1,500*g* for 15 min to obtain plasma for corticosterone measurement. Plasma corticosterone levels were measured using an ELISA kit (Enzo, ADI-900-097), according to the manufacturer’s instructions.

### Ponesimod treatment

Ponesimod (30 mg kg^–1^) or vehicle (0.25% (w/v) methylcellulose with 0.05% (v/v) Tween 80) was intraperitoneally administered once daily, 1 h before the start of CRS. Moreover, on the days of the behavioural tests, a single dose was given 1 h before the start of each test. After the tests were completed, blood and brain tissue samples were collected for plasma corticosterone measurements and IHC, respectively. US and CRS mice received identical ponesimod doses, whereas the vehicle controls received just the vehicle. For [Ca^2+^]_i_ imaging experiments, the mice received a single injection of ponesimod or vehicle. We comment briefly on the known off-target effects of ponesimod. The FDA drug approval package for ponesimod (application number: 213498; www.accessdata.fda.gov/drugsatfda_docs/nda/2021/213498Orig1s000TOC.html) reports known off-target binding activity. Ponesimod showed greater than 50% inhibition of binding to three targets: endothelin ETA receptor, around 74% inhibition; monoamine oxidase MAO-B, about 62% inhibition; and protein serine/threonine kinase Ca^2+^/calmodulin-dependent II (CaMKII), about 63% inhibition. However, several factors were suggested to show that these off-target effects are negligible. Principally, the approximately 1,750-fold difference between the inhibition constant of S1PR1 (5.7 nM) and the screening concentration (10 μM) suggests that at clinically relevant doses, the effects of ponesimod will be dominated by S1PR1 activation. Nonetheless, off-target effects should be considered in future studies that develop S1PR1 or the other GPCRs we report here.

### Neuroinflammation induction with LPS

Neuroinflammation was induced with 5 mg kg^–1^ intraperitoneal injections of LPS, exactly as previously reported^78^. After 24 h, the effects of neuroinflammation were confirmed by reduced ambulation metrics in OFTs, and the mice were euthanized for tissue collection.

### Stereotaxic microinjections of AAVs

All surgical procedures were conducted under general anaesthesia using continuous isoflurane (induction at 5%, maintenance at 1–2% v/v). The depth of anaesthesia was monitored continuously and adjusted as necessary. After the induction of anaesthesia, the mice were fitted into a stereotaxic frame with their heads secured by blunt ear bars and their noses placed into a veterinary-grade anaesthesia and ventilation system (VetEquip). Mice were administered with 0.1 mg kg^–1^ buprenorphine (Buprenex) subcutaneously before surgery. The surgical incision site was then cleaned 3 times with 10% povidone iodine and 70% ethanol (v/v). Skin incisions were made, followed by two craniotomies of 2–3 mm in diameter using a small steel burr (Fine Science Tools) powered by a high-speed drill (K.1070, Foredom). Saline (0.9%) was applied onto the skull to reduce heating caused by drilling. Bilateral viral injections were performed using a stereotaxic apparatus (David Kopf Instruments) to guide the placement of bevelled glass pipettes (1B100-4, World Precision Instruments). For the BLA, the following coordinates were used: 1.45 mm posterior to bregma; 3.20 mm lateral to the midline; and 3.75 mm from the pial surface. AAVs were injected by using a syringe pump (Pump11 PicoPlus Elite, Harvard Apparatus). After AAV microinjections, glass pipettes were left in place for at least 10 min before being slowly withdrawn. Surgical wounds were closed with external 4-0 silk sutures. After surgery, mice were allowed to recover overnight in cages placed partially on a low-voltage heating pad. Buprenorphine was administered twice a day for up to 2 days after surgery. AAV-injected mice were used for experiments at least 2 weeks after surgery. All AAV titres were adjusted to 1.0 × 10^13^ genome copies per ml with sterile 0.1 M PBS. The following viruses were used: 0.15 μl AAV2/5 GfaABC1D-tdTomato (Addgene, 44332); 0.15 μl AAV2/5 GfaABC1D-hM4Di–mCherry (Addgene, 92286); 0.15 μl AAV2/5 GfaABC1D-hM3Dq–mCherry (Addgene, 92284); 0.15 μl AAV2/5 GfaABC1D-cyto–GCaMP6f (Addgene, 52925); 0.15 μl AAV2/5 GfaABC1D-LCK–GFP (Addgene, 105598); 0.15 μl AAV2/5 GfaABC1D-LCK–BioID2-BioID2–HA (Addgene, 176741); 0.15 μl AAV2/5 GfaABC1D-eGFP (Addgene, 176861); and 0.15 μl AAV2/5 GfaABC1D-Cre-4x6T (Addgene, 196410).

### IHC

For transcardial perfusion, mice were euthanized with isoflurane and perfused with 0.1 M PBS followed by 10% buffered formalin (Fisher SF100-20). After gentle removal from the skull, the brain was post-fixed in 10% buffered formalin overnight at 4 °C. The brains were then cryoprotected in 30% sucrose with 0.1 M PBS solution for at least 48 h at 4 °C until use. Serial coronal sections (40 μm) containing the amygdala were prepared using a cryostat microtome (Leica) at −20 °C and processed for IHC. Sections were washed 3 times in 0.1 M PBS for 5 min each and then incubated in a blocking solution consisting of 10% normal goat serum in 0.1 M PBS with 0.5% Triton-X100 for 1.5 h at room temperature with agitation. Sections were then incubated with agitation in primary antibodies diluted in blocking solution overnight at 4 °C. The following primary antibodies were used: guinea pig anti-NeuN (1:1,000; Synaptic Systems, 266004); rabbit anti-FOS (1:1,000; Synaptic Systems, 226008); chicken anti-mCherry (1:1,000; Abcam, ab205402); chicken anti-GFAP (1:1,000; Abcam, ab4674); rabbit anti-S100β (1:1,000; Abcam, ab41548); guinea pig anti-S100β (1:500; Nittobo, MSFR105350); chicken anti-S100β (1:500; Synaptic Systems, 287006), rabbit anti-HA tag (1:1,000; Abcam, ab9110); rabbit anti-septin 2 (1:100; Thermo Scientific, 11397-1-AP); rabbit anti-ARL13B (1:500; Proteintech, 17711-1-AP); rat anti-ARL13B (1:500; BiCell Scientific, 90413); rabbit anti-S1PR1 (1:50; Invitrogen, PA1-1040); rabbit anti-AC3 (1:4,000; Invitrogen, PA5-35382); rabbit anti-SOX9 (1:500; Millipore, AB5535); goat anti-collagen IV (1:500; Novus Bio, NBP1-26549); and biotinylated WFA (1:1,000; Vector labs, B-1355-2). For IHC of collagen IV, the sections were pre-treated with pepsin (Sigma, R2283) for 5 min at 37 °C and washed with PBS at 27 °C for 8 min. Sections were washed 4 times in PBS-T for 5 min and then incubated at room temperature for 1 h with secondary antibodies diluted in blocking solution. The following Alexa-conjugated secondary antibodies (1:1,000; Molecular Probes) were used: Alexa Fluor 488 goat anti-guinea pig (A11073), Alexa Fluor 546 goat anti-chicken (A11040), Alexa Fluor 647 goat anti-rabbit (A21244) and streptavidin Alexa Fluor 647 conjugate (S21374). The sections were rinsed 3 times in PBS-T for 5 min and then 3 times in 0.1 M PBS for 5 min before being mounted on microscope slides and sealed in ProLong Glass Antifade (P36984, Invitrogen). Fluorescent images were taken using UPlanXApo ×4 (NA 0.16), UPlanXApo ×10 (NA 0.40), UPlanFL N ×40 (NA 1.30) and UPlanXApo ×60 (NA 1.42) objective lenses on a confocal laser-scanning microscope (FV3000; Olympus) using Fluoview software (Olympus). Laser settings for imaging were kept the same in each experiment. Images represent maximum-intensity projections of optical sections with a step size of 0.5–1.0 μm. Images were processed usinf ImageJ (v1.53u–1.54p). Non-ciliary ARL13B rabbit antibody background staining was sometimes thresholded out of the figure images to improve identification of the primary cilium compared with the background. Example raw images from flattened *z* stacks are provided in Fig. 2n and Extended Data Fig. 4h. No thresholding was applied to IHC images showing primary cilium staining with ARL13B rat and AC3 antibody, or to any other images.

3D rendering of IHC images was performed using Imaris software (Bitplane, Oxford Instruments). Raw image stacks (0.5 µm) were imported into Imaris and visualized using the Surpass mode to generate 3D representations of the labelled structures. Then, using Imaris software, the following steps were performed to reconstruct the surface and to calculate the volume: selection of a region of interest containing the cilium for segmentation, smoothing with a surface grain size detail of 0.3–0.4 µm (most often 0.4 µm), threshold using the background subtraction method with diameter of largest sphere of 1–1.6 µm (most often 1 µm). This was followed by manual value threshold selection guided by (1) maintaining maximum resemblance to IHC cilium staining captured and (2) ensuring the cilium was rendered as one volume. For rendering of the astrocyte soma surface, the same protocol was followed except that thresholding was performed with the absolute intensity method instead of the background subtraction method. 3D renderings were adjusted for transparency, colour and lighting to facilitate visualization. Volume analysis data were extracted from the 3D reconstructions using the Imaris MeasurementPro module.

### RNAscope of mice and human tissue

Fixed-frozen tissue was processed as described above. Serial coronal sections (20 μm) containing amygdala were prepared using a cryostat microtome (Leica) at −20 °C and mounted immediately onto glass slides. Dual ISH–IHC was performed using a Multiplex RNAscope (v.2) with integrated co-detection workflow (ACDBio, 323180 and 323110). Sections were baked for 30 min at 60 °C. Sections were washed for at least 15 min in 0.1 M PBS and then incubated in target retrieval reagents for 5 min at 95 °C. After washing with ddH_2_O twice, the sections were dehydrated with 100% ethanol and dried at room temperature. Sections were then incubated with primary antibody guinea pig anti-S100β (1:500; Nittobo, MSFR105350) or chicken anti-S100β (1:500; Synaptic Systems, 287006) overnight at 4 °C. Sections were then incubated with protease (ACDBio, 322330) for 30 min at 40 °C. The sections were washed with ddH_2_O twice for 1 min each and then incubated with the corresponding probe for 2 h at 40 °C: Mm-Arl6-C2 (ACDBio, 492301-C2), a custom probe for mouse ARL3-C1 (ACDBio) or Mm-S1PR1-C2 (ACDBio, 426001-C2).

The sections were incubated in AMP 1-FL for 30 min, AMP 2-FL for 30 min and AMP 3-FL for 15 min at 40 °C while washing in wash buffer (ACDBio, 310091) between incubations. The HRP-C1 signal was developed with Opal 520 fluorophore (Akoya Biosciences, FP1487001KT). The HRP-C2 signal was developed with Opal 570 fluorophore (Akoya Biosciences, FP1488001KT). All incubations at 40 °C or 60 °C were performed in a HybEZ hybridization system (ACDBio). Last, sections were incubated with Alexa Fluor goat secondary antibodies described in the IHC section for 30 min at room temperature. Images were obtained in the same way as for IHC (described above) with a step size of 1 μm. Images were processed using ImageJ (v.1.53u–1.54p). Astrocyte somata were labelled with S100β, and the presence of puncta in each soma was quantified.

For RNAscope of human brain tissue, a RNAscope Multiplex Fluorescent Reagent kit v.2 (all reagents from ACDBio) was used with the astrocyte-specific C1 probe *ALDH1L1* and the *S1PR1* target C2 probe according to the user manual with modifications. In brief, 18 μm coronal sections of fresh-frozen human amygdala samples from four neurotypical individuals (*n* = 2 male and *n* = 2 female individuals) were fixed with 4% paraformaldehyde in PBS for 45 min at 4 °C, rinsed with PBS and dehydrated with gradient ethanol solutions. Endogenous peroxidase activity was quenched with hydrogen peroxide solution for 10 min, and protease IV reagent was used for 30 min for proteolytic tissue digestion. After hybridization steps, the signal was developed with TSA Vivid Fluorophores 570 and 650, both at 1:1,000 dilution in TSA buffer. Slides were then counterstained with DAPI, and lipofuscin autofluorescence was quenched by incubating the slides for 1 min with 1× TrueBlack solution (Biotum, 23007). After autofluorescence quenching, slides were rinsed 3 times with PBS and mounted with ProLong Gold Antifade reagent (Thermo Fisher). Four *z* stack images under ×20 magnification with a step size of 1 μm were acquired per sample from the basolateral region of the amygdala with a Leica SP8 confocal microscope. A total of 632 *ALDH1L1*-positive nuclei were manually counted using Fiji. The data are expressed as the percentage of S1PR1-positive astrocytes per each case.

### Calcium imaging of astrocytes in brain slices

In brief, mice were anaesthetized with isoflurane and decapitated with sharp shears. The brains were placed and sliced in ice-cold modified artificial cerebrospinal fluid (aCSF) containing the following components: 194 mM sucrose, 30 mM NaCl, 4.5 mM KCl, 1 mM MgCl_2_, 26 mM NaHCO_3_, 1.2 mM NaH_2_PO_4_ and 10 mM d-glucose, saturated with 95% O_2_ and 5% CO_2_. A vibratome (DSK Microslicer; Ted Pella) was used to cut 300 μm brain sections. The slices were allowed to equilibrate for 30 min at 33 °C in normal aCSF containing the following components: 124 mM NaCl, 4.5 mM KCl, 2 mM CaCl_2_, 1 mM MgCl_2_, 26 mM NaHCO_3_, 1.2 mM NaH_2_PO_4_ and 10 mM d-glucose, continuously bubbled with 95% O_2_ and 5% CO_2_. Slices were then stored at 21–23 °C in the same buffer until use. All slices were used within 6 h of slicing. Brain-slice imaging was performed at room temperature (21–23 °C). Cells for all the experiments were imaged using a confocal microscope (Fluoview 1000; Olympus) with a ×40 water-immersion objective lens with 0.8 NA and a digital zoom of 2–3. We used the 488 nm line of an Argon laser, with the intensity adjusted to 5–10% of the maximum output of 10 mW. The emitted light pathway consisted of an emission high-pass filter (505–525 nm) before the photomultiplier tube. Astrocytes were typically around 25 μm below the slice surface and were scanned at 1 frame per s for imaging sessions. For pharmacological activation of endogenous GPCRs, agonists were dissolved in water. The DREADD agonist DCZ and the S1PR1 agonist ponesimod were dissolved in DMSO. Stock solutions were diluted in aCSF immediately before use. Analyses of time-lapse image series were performed using ImageJ (v.2.1, NIH). The *xy* drift was corrected using ImageJ, and cells with *z* drift were excluded from analyses. Time traces of fluorescence intensity were extracted from the regions of interest and converted to change in fluorescent (Δ*F/F*) values. Traces of the regions of interest were extracted using GECIquant software (v.1.0). Extracted Ca^2+^ signals were analysed using custom R (v.4.4.1) scripts. Events were identified on the basis of amplitudes that were at least threefold above the baseline noise of the Δ*F/F* trace and analysed using the findpeaks function (pracma v.1.9.9) for event identification and quantification, the AUC function (DescTools v.0.99.55) for area under the curve calculation and tidyverse (v.2.0.0) for data manipulation.

### Extraction of astrocytic RNA from *Aldh1l1*^*cre/ERT2*^;RiboTag mice

Extraction of astrocyte-derived RNA from *Aldh1l1*^*c*^^*re/ERT2*^;RiboTag mice was done as previously described^75^. In brief, tissue from the amygdala were collected from 16 animals of 2–3-month-old *Aldh1l1*^*cre/ERT2*^;RiboTag mice (8 males and 8 females, 4 mice pooled per sample, 4 samples per group) and homogenized. RNA was extracted from 10–20% of cleared lysate as input containing RNA from all cell types using a RNA extraction kit (Qiagen, 74034). The remaining lysate was incubated with 5 µl mouse anti-HA antibody (about 1:160, BioLegend, 901514) for 4 h at 4 °C followed by addition of magnetic beads (Thermo Scientific, 88817) and overnight incubation at 4 °C. RNA was extracted from the IP samples containing astrocyte-enriched RNA using an RNA extraction kit (Qiagen, 74034). RNA concentration and quality were assessed using an Agilent 2100 Bioanalyzer.

### Astrocyte RNA-seq and analysis

RNA samples with RNA integrity number greater than 7.8 were used for subsequent multiplexed library preparation with TruSeq Stranded Total RNA with Ribozero Gold, except the MACS and the *Ift88*^*f/f*^ bulk samples, for which library preparation was done with SMART-Seq mRNA and NexteraXT. For each experiment, all samples were multiplexed into a single pool to avoid batch effects. Sequencing was performed on NovaSeq 6000 or NovaSeq × Plus, which produced at least 80 million reads per sample. Demultiplexing was performed using Illumina Bcl2fastq2 (v.17). Reads were aligned to the mouse mm10 reference genome using the STAR spliced read aligner (v.2.7), and 75 ± 16% of the reads were uniquely mapped. Previously reported datasets of the 13 regions of the CNS^58^ were combined with the amygdala dataset by batch correction using Removing Unwanted Variation (RUVr). All DEGs were obtained using limma-voom. Differential gene expression analysis was performed with the Bioconductor package limmaVoom (v.3.60) with the adjusted *P* value threshold set at <0.05. Astrocyte-enriched genes were defined as those with log_2_[IP/input] > 1, adjusted *P* < 0.05 and IP FPKM > 1. For analysis of astrocyte region-specific genes, genes were analysed by comparing IP samples with the average of IP samples from the other 13 regions using limma-voom. GO pathway analysis was performed using Enrichr (https://maayanlab.cloud/Enrichr/). Unwanted variation in the US, CRS and WAS datasets was removed using RUVr. *S1pr1* RNA-seq expression in cortex astrocytes for Fig. 6 was calculated by averaging that of the SCX, the MCX and the VCX^58^.

### Cilium-related genes

The list of 770 cilium-related genes to compute region enrichment was obtained by combining genes from the GO terms ‘cilium’ (GO:0005929), ‘cilium assembly’ (GO:0060271), ‘cilium organization’ (GO:0044782) and ‘axoneme’ (GO:0005930). The final list of 770 genes shared 60% overlap with Syscilia Gold Standard (SCGv1) of known ciliary components^79^ and 42% overlap with a longer list of cilia-related genes from CiliaCarta^80^. The full list of genes is provided in Source Data Fig. 4.

### In vivo BioID2 protein biotinylation and pull-downs

Three weeks after AAV microinjection of BioID2 or GFP (control), mice were treated with a subcutaneous injection of biotin (24 mg kg^–1^; Millipore Sigma, RES1052B-B7) dissolved in sterile 0.1 M PBS once per day for 7 consecutive days. Mice were used 16 h after the last biotin injection.

Purification of biotinylated proteins was conducted as previously described^81,82^. Eight mice were used for each biotinylated protein purification. Amygdala tissue (8 mice pooled per sample, 4 samples per group, equal numbers of male and female mice) was dounce-homogenized in lysis buffer A (1 mM EDTA, 150 mM NaCl and 50 mM HEPES pH 7.5 supplemented with Halt protease inhibitor (Thermo Scientific, 78429)). Immediately after homogenization, lysis buffer B (2% sodium deoxycholate, 2% Triton-X, 0.5% SDS, 1 mM EDTA, 150 mM NaCl and 50 mM HEPES pH 7.5) was added. The lysed samples were sonicated for 5 min at 60% power and then centrifuged at 15,000*g* for 15 min at 4 °C. The resulting supernatant was then ultracentrifuged at 100,000*g* for 30 min at 4 °C. SDS was added to the supernatant to obtain a final concentration of 1%. The sample was then boiled at 95 °C for 5 min. The sample was cooled on ice and incubated with 35 μl equilibrated anti-pyruvate carboxylase (5 μg; Abcam, 110314) conjugated agarose beads (Pierce, 20398) for 4 h at 4 °C while rotating. Subsequently, the sample was centrifuged at 500*g* for 5 min at 4 °C and the supernatant was incubated with Pierce streptavidin magnetic beads (Thermo Scientific, 88817) at 4 °C overnight while rotating. The magnetic beads were then washed twice with 0.2% SDS, twice with wash buffer (1% sodium deoxycholate, 1% Triton-X and 25 mM LiCl), twice with 1 M NaCl and 5 times with 50 mM ammonium bicarbonate. Proteins bound to the beads were then eluted in elution buffer (5 mM biotin, 0.1% Rapigest SF surfactant and 50 mM ammonium bicarbonate) at 60 °C for a minimum of 2 h.

### Analysis of biotinylated proteins by mass spectrometry

Eluates obtained after IP were subjected to reduction and alkylation using 5 mM Tris(2-carboxyethyl)phosphine and 10 mM iodoacetamide, respectively, followed by treatment with single-pot, solid-phase-enhanced sample preparation protocol (SP3) for clean-up of protein. The eluates obtained after SP3 protein clean-up were subjected to overnight digestion with Lys-C and trypsin at 37 °C. The digested peptides were treated with SP3-based peptide clean-up protocol, dried and subsequently analysed by LC–MS/MS. In brief, reversed-phase chromatography was used to separate peptides on Bruker PepSep C18 columns (150 μm i.d., 15 cm in length and 1.5 μm particle size) using a gradient of increasing acetonitrile delivered by a Vanquish Neo UHPLC-system (Thermo Scientific) operating at a flow rate of 500nl min^–1^. Data acquisition was carried out on a Bruker timsTOF HT mass spectrometer using a diaPASEF mode of data acquisition. The dia-PASEF windows spanned a precursor range from 300 to 1,200 *m/z* using the following ion mobility parameters: 1/K0 start, 0.60 Vs cm^−2^; 1/K0 end, 1.60 Vs cm^−2^; ramp time, 100 ms; accumulation time, 50 ms.

LC–MS/MS analysis for ARL13B–BioID experiments was carried out on a Vanquish Neo UHPLC system coupled to an Astral mass spectrometer (Thermo Fisher Scientific). Peptide separation was performed using a trap-and-elute workflow on an ionopticks C18 reverse-phase column (8 cm × 150 µm, 1.7 µm particle size) maintained at 52 °C. Mobile phase A consisted of water containing 0.1% formic acid, whereas mobile phase B consisted of acetonitrile with 0.1% formic acid. A 15-min gradient was applied as follows: 5% B from 0 to 1 min (2.45 µl min^–1^); 5–15% B from 1 to 5 min (1.75 µl min^–1^); 15–25% B from 5 to 12.6 min (1.75 µl min^–1^); 25–38% B from 12.6 to 13.6 min (1.75 µl min^–1^); 38–80% B from 13.6 to 13.7 min (2.45 µl min^–1^); followed by a hold at 80% B for 15 min (2.45 µl min^–1^). Data-independent acquisition (DIA) was performed on an Orbitrap Astral mass spectrometer operating in positive electrospray ionization mode. MS1 spectra were acquired at 240,000 resolution across an *m/z* range of 380–980, with a normalized AGC target of 500% and a maximum injection time of 3 ms. DIA used sequential 4 *m/z* isolation windows covering a precursor range of 380–980 *m/z*. MS2 scans were acquired at 80,000 resolution using a normalized HCD collision energy of 25%, a normalized AGC target of 500% and a maximum injection time of 7 ms. For ARL13B proteomics, we had to use the cortex instead of the amygdala because the number of mice needed to perform the proteomic experiment (about 64) was prohibitive because of the small size of the amygdala. However, as there is limited information on the astrocyte primary cilium proteome, the cortex was a good place to start because astrocytes have ARL13B-positive primary cilia in the cortex and multidimensional scaling has shown that amygdala and cortical astrocytes cluster together at a molecular level.

The dia-PASEF files and the Thermo RAW files obtained after LC–MS/MS analysis were searched using DIA-NN (v.1.8.1) with an in silico library generated from a UniProt database containing all mouse proteins^83^. Statistical analysis of hits and DEPs was done using the Bioconductor package limma (v.3.60). A protein was declared a hit when it satisfied the following filters: log_2_[BioID2/GFP] > 1 and adjusted *P* < 0.05. GO pathway analysis was performed using Enrichr (https://maayanlab.cloud/Enrichr/). Putative localization in the primary cilium of high probability hits from proteomics was based on the following published work: TULP3 (ref. ^84^), ARL3 (ref. ^85^), IFT74 (ref. ^86^), TOGARAM1 (ref. ^87^), MAP4 (ref. ^88^), SNAP29 (ref. ^89^), RAB34 (ref. ^90^), EHD1 (ref. ^89^), DLG1 (ref. ^91^), EZR^92^, INPP5E^93^ and IFT43 (ref. ^94^).

### Molecular cloning and PHP.eB AAV generation

To generate the ARL13B–BioID2 and GFP plasmids, the Astro-BioID2 (Addgene plasmid 176740) or Astro-GFP (Addgene plasmid 176861) plasmid was cut by restriction digestion at the XhoI site. cDNA for *ARL13B* was amplified from Addgene plasmid 232880 (ref. ^95^) and cloned into the XhoI site using In-fusion cloning (Takara Bio). The plasmids were packaged into PHP.eB AAVs in-house following a published protocol^96^.

### Retroorbital delivery of PHP.eB AAVs

Brain-wide expression of ARL13B–BioID2 and ARL13B–GFP in astrocytes in 8-week-old mice was achieved by retro-orbital injection into the eye sinus with 10^12^ genome copies per mouse of AAV-PHP.eB-GfaABC1D-ARL13B-BioID2-HA or AAV-PHP.eB-GfaABC1D-ARL13B-GFP^97^. The biotin injections were performed as indicated above. The entire cortex was dissected and used for streptavidin-mediated pull-down of biotinylated proteins as indicated above. The plasmids are available from Addgene (identifiers 253927 and 253928).

### CilioGenics probability

Data were downloaded^98^ from https://ciliogenics.com/?page=Home. Proteins were assigned a probability on the basis of the assigned CilioGenics score as per the website: high = 1, medium = 1–0.5, low < 0.5.

### MACS-based astrocyte isolation and qPCR

The protocol was adapted from a previous study^99^. Amygdala tissue collected from 3–4 adult mice was homogenized together for each sample. The tissue was incubated in papain at 37 °C for 30 min with a constant supply of 95% O_2_ and 5% CO_2_ on the liquid surface, then centrifuged and resuspended in cold PBS, filtered through a 70 µm cell strainer and debris was removed with Debris Removal solution (130-109-398, Miltenyi Biotec) following the manufacturer’s instructions. The resulting supernatant was resuspended in 90 µl 0.5% BSA (A7030, Millipore Sigma) containing 1 mM EDTA (15575-020, Invitrogen) and incubated with 10 µl FcR blocking beads (130-097-678, Miltenyi Biotec) for 10 min at 4 °C, followed by the addition of 10 µl ACSA-2 beads and incubation for 15 min at 4 °C. Then, 2 ml BSA–EDTA was added, the solution was centrifuged, resuspended in BSA–EDTA and filtered through a MS column (130-042-201, Miltenyi Biotec) placed on a MACS magnet. The column was eluted with BSA–EDTA and RNA was isolated with a RNA extraction kit (Qiagen, 74034). cDNA was obtained from RNA with SuperScript IV (18090010, Invitrogen) following the manufacturer’s instructions. qPCR was performed with the following primers: *Aldh1l1*, *Rbfox3* and *Aif1* sequences have been previously described^100^; *Slc1a3* fw 5′-GCGATTGGTCGCGGTGATAATG-3′; *Slc1a3* rv 5′-CGACAATGACTGTCACGGTGTAC-3′; *Mbp* fw 5′-ATTCACCGAGAGGCTGGAA-3′; *Mbp* rv 5′-TGTGTGCTTGGAGTCTGTCACC-3′; *Ppia* fw 5′-CATACAGGTCCTGGCATCTTGTC-3′; and *Ppia* rv 5′-AGACCACATGCTTGCCATCCAG-3′. Gene expression was calculated as *C*_*t*_ values relative to the housekeeping gene *Ppia* in each fraction (astrocyte or input) and then plotted as the ratio of the *C*_*t*_ values of the two fractions.

### Human amygdala astrocyte snRNA-seq analysis

Frozen amygdala tissue samples from 120 brain donors were obtained from the Harvard Brain Tissue Resource Center. Sets of 20 brain tissue specimens (each consisting of donors with PSDs and unaffected individuals) were processed at once as a single pooled sample, including nucleus extraction, generation of gel beads-in-emulsion and library preparation performed according to the 10x Chromium Single Nuclei 3′ v3.1 protocol. Raw sequencing reads were aligned to the hg38 reference genome using the standard Drop-seq (v.2.5.4) workflow. Reads were assigned to annotated genes if they mapped to exons or introns of those genes. Ambient or background RNA was removed from digital gene expression matrices using CellBender (v.0.3.0) remove-background. Dropulation (v.2.5.4) was used to assign each nucleus to its donor of origin on the basis of transcribed single-nucleotide polymorphisms. Cell-type assignment was accomplished with cell-classification models trained using scPred (v.1.9.2).

scRNA-seq data from astrocytes were pseudo-bulked by summing raw gene counts across all astrocyte cells (with ≥200 detected genes, to exclude low-quality nuclei) from each donor. Donors with fewer than 30 astrocytes were excluded, which resulted in 109 donors (71 with PSDs and 38 unaffected individuals; mean age 65.07 ± 17.21 years; *n* = 53 women). Differential expression analyses were performed in edgeR (v.4.2.1) using quasi-likelihood negative binomial regression with glmQLFit(), and testing was carried out with glmQLFTest() for the contrast between PSD and control donors. Normalization was conducted using the TMM method via calcNormFactors(), and lowly expressed genes were filtered with filterByExpr(). Age, sex and data collection batch were included as covariates in the design matrix, and multiple testing correction was performed using the Benjamini–Hochberg method to control the false discovery rate. We report the astrocyte data for the primary cilium-related genes here, but a follow-up study from the laboratory of S.A.M. will report all cell types of the amygdala in due course.

### Statistical analysis and data presentation

Data from every experiment represent at least three replicates. Sample sizes were based on previous experiments using similar or identical experiments and the use of similar models by our laboratory. Statistical tests, unless otherwise stated, were run in OriginPro 2024. In bar plots, data are presented as the mean (bar) ± s.e.m. (error bars) along with the individual data points. The results of statistical comparisons, *n* numbers and significance levels are shown in the figure panels along with the average data. *n* is defined as the number of cells or mice on a case-by-case basis throughout the paper. We determined whether each set of data was normally distributed using OriginPro 2024. If the data were normally distributed, we used parametric tests; if they were not normally distributed, we used nonparametric tests. Unpaired Student’s two-tailed *t*-tests, two-tailed Mann–Whitney tests and one-way and two-way analysis of variance (ANOVA) tests were used for most statistical analyses comparing two groups. One-way ANOVA followed by Tukey’s post-hoc test or Kruskal–Wallis ANOVA followed by Dunn’s test were used for statistical analyses comparing three or more groups. Significance was declared at *P* < 0.05. When *P* values were greater than 0.05, they are stated as not significant. When the *P* value was less than 0.01, it is stated as <0.01. When the *P* value was less than 0.001, it is stated as <0.001. All transcriptomic and proteomic analyses used a statistical adjusted *P* < 0.05 unless otherwise stated. No data points were excluded from any experiment. Data used to generate the graphs shown in the figures are provided in the Source Data files. The results of all statistical tests are provided in Supplementary Table 1.

### Reporting summary

Further information on research design is available in the Nature Portfolio Reporting Summary linked to this article.

## Online content

Any methods, additional references, Nature Portfolio reporting summaries, source data, extended data, supplementary information, acknowledgements, peer review information; details of author contributions and competing interests; and statements of data and code availability are available at https://doi.org/10.1038/s41586-026-10874-0.

## Supplementary information

Reporting Summary

Supplementary Table 1 Statistical tests used and their results.

Supplementary Table 2 RNA-seq data for US, CRS and WAS.

Supplementary Table 3 Proteomic data for US, CRS and WAS.

Supplementary Table 4 List of predicted cilium-enriched genes used for data analyses.

Supplementary Table 5 RNA-seq data for the LPS experiment and RNA-seq and proteomic data for the hM4Di experiment.

Supplementary Table 6 Shared astrocyte DEGs and region-enriched DEGs for the 14 CNS regions.

Supplementary Table 7 RNA-seq data for cilium floxed mice.

Supplementary Table 8 Phenopedia cilium-related genes expressed in amygdala astrocytes.

Supplementary Table 9 Cilium-related gene expression changes in human amygdala astrocytes from patients with MDD.

Supplementary Table 10 Cilium-related gene expression changes in human amygdala astrocytes from patients with PSD.

Supplementary Table 11 Proteomic data for ARL13B–BioID2

Peer Review File

## Source data

Source Data Fig. 1

Source Data Fig. 2

Source Data Fig. 3

Source Data Fig. 4

Source Data Fig. 5

Source Data Extended Data Fig. 1

Source Data Extended Data Fig. 3

Source Data Extended Data Fig. 4

Source Data Extended Data Fig. 5

Source Data Extended Data Fig. 6

Source Data Extended Data Fig. 7

Source Data Extended Data Fig. 8

Source Data Extended Data Fig. 9

Source Data Extended Data Fig. 10

Source Data Extended Data Fig. 12

Source Data Extended Data Fig. 13

Source Data Extended Data Fig. 15

Source Data Extended Data Fig. 16

## Data Availability

All new astrocyte RNA-seq data generated in this study have been deposited into the Gene Expression Omnibus with accession identifier GSE285150. Previously published data for the 13 regions of the brain can be found with the accession identifier GSE198024. All new astrocyte proteomic data generated in this study have been deposited into the MassIVE repository and can be accessed using the identifier MSV000096647. Lists of DEGs, hits and DEPs for all experiments can be found in the Supplementary Information. Source data are provided with this paper.

## Code availability

For RNA-seq, differential gene expression and enrichment analysis used the R package limma-voom to process RNA counts (v.3.60; https://rdrr.io/bioc/limma/man/voom.html) and batch correction and removal of unwanted variation was done with RUVr^101^. scRNA-seq data were analysed using Seurat (https://CRAN.R-project.org/package=Seurat). Visualization of set intersections was done with UpSetR (v.1.4.0; https://github.com/hms-dbmi/UpSetR). For proteomics, label-free quantification was carried out using DIA-NN (v.1.8.1). Principal component data visualization was conducted with the R package DESeq2 (v.1.44.0; https://bioconductor.org/packages/release/bioc/html/DESeq2.html). Differential protein expression and enrichment analysis was conducted using limma (v.3.60; https://bioconductor.org/packages/release/bioc/html/limma.html). For calcium imaging, data were analysed in R using GECIquant (v.1.0), pracma (v1.9.9; https://cran.r-project.org/web/packages/pracma/index.html) and DescTools (v.0.99.55; https://cran.r-project.org/web/packages/DescTools/index.html).
