# Supplementary material for: Amygdala astrocyte primary cilium mechanisms contribute to stress behaviours
Source: Nature. 2026 Aug 5;657(8131):455–68. doi: 10.1038/s41586-026-10874-0 (PMC13558064; doi:10.1038/s41586-026-10874-0)
Supplement: Supplementary file 1 [file 41586_2026_10874_MOESM13_ESM.pdf]

nature portfolio

Peer Review File

Amygdala astrocyte primary cilium mechanisms contribute to stress behaviors

Corresponding Author: Professor Baljit Khakh

Any redactions in this file are there to maintain patient confidentiality, the confidentiality of unpublished data, or to remove third-party material.

This file contains all reviewer reports in order by version, followed by all author rebuttals in order by version.

Version 0:

Reviewer comments:

Referee #1

(Remarks to the Author)
In this study, the authors investigate the role of astrocytes in the mouse amygdala in mediating stress-related behaviours. They report a link between ciliated astrocytes in the amygdala and stress-related behaviours. Upon exposure of mice to distinct paradigms of stress, amygdala astrocytes undergo changes in gene expression and their cilia become shorter. They also show that the GPCR receptor S1PR1, expressed on amygdala astrocytes, when activated with Ponesimod mitigates the effect of stress on cilia length and leads to improved physiological and behavioural changes in mice upon exposure to stress. Although this study provides an interesting link between stress and ciliated astrocytes, the biological significance of the results is not clear and the relevance of these results to human neuropsychiatric disorders is unknown . Moreover, the study lacks sufficient molecular or circuit level mechanistic insights into the link between astrocytes, their cilia, and stress responses in the mouse brain.
Additional Major Concerns
1. What is the level of change in primary cilia length that would be biologically meaningful? In this study, the change in cilia length in amygdala astrocytes does not appear robust. For example, the changes shown in Figure 1 appear rather modest with large variation. Also, it is not clear whether and how the study was powered to detect biologically meaningful changes in primary cilia length.
2. What is the relevance of the distinct paradigms used in this study in human neuropsychiatric disorders? In other words, what is the predictive validity of finding a change in primary ciliary length in such a paradigm?
3. Are the changes in cilia genes and cilia length in mice exposed to stress specific to astrocytes or do the changes also occur in other cell types such as excitatory neurons or inhibitory interneurons? The authors should consider investigating the transcriptomic changes in their mouse models with single cell sequencing to evaluate how cilia gene changes in astrocytes impact the transcriptome of the other cell types that interact with astrocytes.
4. The authors do not provide a mechanistic molecular or circuit level link of how deregulation of primary cilia length might lead to functional changes that cause a behavioural outcome. In the absence of such insights, the study is premature and the claim that targeting astrocytic primary cilia might provide a novel treatment strategy for stress-related conditions is an overstatement.
5. Ponesimod is a specific agonist of the S1PR; however, in effect it acts as a functional antagonist (Kruger et al., 2023). Upon binding to the S1PR, it leads to internalisation of the receptor therefore acting as a functional antagonist and reduction in overall S1P signalling in the periphery. The authors should characterize how ponesimod activity on the S1PR on astrocytes links to changes in cilia. In addition, is expression

of S1P or S1PR1 deregulated in the amygdala of patients with neuropsychiatric disorders?

Minor Comments:

1. The authors describe chronic restraint stress (CRS) as having the more robust phenotype but the cilium length change was less significant than with water avoidance stress (WAS) ($p<0.05$ vs $p<0.005$), and the volume having a greater change in CRS than in WAS, are the differentially expressed cilium genes able to account for this morphological difference? Figure 2j.
2. Can the authors detect a reduction in GFAP in the CSF as they report a reduction in GFAP+ cells in the amygdala with CRS and WAS? Figure 1h.
3. Image 1h shows a reduction in GFAP intensity rather than a reduction in the number of GFAP+ cells in the nuclei.
4. Can the authors speculate why CRS and WAS produced cilium genes that were unique given that the significantly changed biological processes (Extended Figure 2e) were by and large the same but have different significance? Was the net outcome the same despite these unique gene differences?
5. The authors could consider moving the molecular properties of amygdala astrocytes to the start of the paper.
6. Error bars should be defined in the figure legends.

Reference:

1. Kruger, T.M., Valenzuela, B., Thompson, C.D., Ouwerkerk-Mahadevan, S., and Ruixo, J.J.P. (2023). Clinical Pharmacokinetics of Ponesimod, a Selective S1P1 Receptor Modulator, in the Treatment of Multiple Sclerosis. Clin. Pharmacokinet. 62, 1533–1550. https://doi.org/10.1007/s40262-023-01308-5.

Referee #2

(Remarks to the Author)
I co-reviewed this manuscript with one of the reviewers who provided the listed reports.

Referee #3

(Remarks to the Author)
Comments on ‘Amygdala astrocytes contribute causally to aberrant behaviors triggered by stress’

The molecular mechanisms linking exposure to stress and aberrant behavior are still mostly unknown. Understanding these mechanisms is important for our ability to treat multiple psychiatric disorders. Pelaz et al. investigated the mechanisms by which amygdala astrocytes influence stress-related behavior. The authors mice to various stress models such as chronic restraint stress (CRS) or water aversion stress (WAS), to induce stress-related behavior. These stressful conditions evoked transcriptional and proteomic responses in amygdala astrocytes. Using immunohistochemistry, the authors detected a reduction in the length and volume of primary cilia in amygdala astrocytes following stress. Moreover, given that importance of GPCR signaling in primary cilia signaling, the authors evaluated the ability of DREADD-driven Gi activation (hM4Di) to rescue stress-evoked behavioral deficits. hM4Di activation was sufficient to rescue the stress-evoked behavioral deficit and length reduction of primary cilia. Finally, based on their finding that the modulation of GPCR signaling is beneficial in the context of stress-related behavior, the authors evaluated the potential therapeutic effect of activating the astrocyte-expressed S1PR GPCR with the clinical-grade agonist Ponesimod, finding that it rescues the behavioral deficits induced by stress.

Given the significant impact of stress on global health in modern society, studying stress-induced behavioral changes and the mechanisms responsible for them is timely. In addition, this study is particularly exciting because as it identifies contributions of amygdala astrocytes to stress-evoked behavioral deficits. Indeed, linking astrocytic primary cilia to behavioral output is highly novel and original. Moreover, the beneficial effects of Ponesimod, a drug already in clinical use, open unexpected therapeutic avenues. The techniques used are cutting-edge, and the data produced is informative and resourceful. The authors are leaders in the field, and the studies are properly controlled and described. However, I believe that some issues have to be addressed before this work is ready for publication.

Major comments

1. Given that cilia are very small structures (~4 µm in length), it would be helpful if the authors could further justify the use of immunohistochemistry for their visualization. Alternatively, reproducing the key findings with methods such as electron microscopy could further strengthen the impact of the study.
2. I am curious about the biological significance of cilia shortening. From a signaling perspective, how does this change affect cellular function, particularly in astrocytes, and what would be expected under homeostatic conditions? Adding such a discussion to the manuscript would provide valuable context and make the paper even more engaging for readers.
3. It would be helpful if the authors could better explain the transition from astrocyte cilia studies to GPCRs. While it is true that GPCR signaling is important in cilia, GPCRs are also broadly critical in many other contexts. Please expand the justification on why the study moves from cilia to GPCRs.
4. In the stress-induced model, Gi-DREADD activation restores cilia length and rescues behavior. However, to establish a stronger link and exclude the potential contribution of other brain regions, it would be valuable to examine how AAV-driven Arl13b inactivation in amygdala astrocytes affects behavior and/or neuronal circuit function.

5. Since much of the work focuses on manipulating and staining for Arl13b, it would be helpful if the authors could clarify why this target was selected. Does Arl13b detected in downregulated cilia-related pathways?
Minor comments
1. In ED Fig. 4a, Arl13b immunoreactivity is a bit dim, and overlay panels can be visually improved for the readers.
2. I cannot find ED Fig. 8a in the main manuscript.
3. Could you please discuss downregulated astrocyte DEGs in Fig. 2b?
4. The authors may want to move some non-significant data from main Figs to Extended Data Figs to bring focus on the significant differences detected.
5. Making the subheadings more specific could the readability of the manuscript.

Referee #4

(Remarks to the Author)
In the current study, Pelaz et al. present findings that amygdala astrocyte GPCRs, specifically S1pr1, contribute casually to molecular and behavioral phenotypes in response to stress. The authors also find that in response to chronic stressors, astrocyte cilia present as smaller and correlate with increased anxiety like behavior, while RNA sequencing of astrocytes demonstrates dysregulated gene expression, including those related to cilia. Near-proteomic approaches, also identify cilium related proteins. Chemogenetic targeting of GPCRs and pharmacological modification of S1pr1 rescued anxiety like behavior and astrocyte cilia changes. Human RNAscope and RNA-seq data also demonstrates alterations in cilia related genes. Finally, the authors demonstrate knocking out the cilia protein Arll3b globally in astrocytes modified gene expression in astrocytes and neurons. This is an interesting paper, providing new information related to gene and protein expression in amygdala astrocytes and how these change in the context of stress. The authors also provide novel and intriguing information related to astrocyte cilia. Finally, this work causally links amygdala astrocyte S1pr1 to the stress response, as noted in the title. The methodological and statistical approaches used are appropriate, and the quality of the data are high. The major concern noted to this manuscript, in its current iteration, is that the results presented do not support the conclusion that astrocyte cilia are causally linked to the stress response. This and several additional concerns are noted below.

MAJOR CONCERNS
1. The authors provide limited background information on cilia and their known functions in astrocytes. Considering the paper is centered around astrocyte primary cilia, additional details regarding this should be included in the Main section of the text and discussion.
2. Similarly, there is no discussion of S1pr1 gene or protein expression in the context of stress, or its ligand S1p- does it change, or is it known to change in the context of stress? The vast majority of S1p comes from blood, it seems this data should exist.
3. The authors demonstrate 1) cilia are different in stress 2) astrocytes have altered gene expression, including cilia related genes 3) targeting S1pr1 improved stress response and cilia 4) S1pr1 is also expressed in human amygdala astrocytes 5) cilia related genes are also abundant in human astrocytes 4) disrupting astrocyte cilia leads to changes in gene expression and changes in neurons. However, they did not show amygdala astrocytes contribute causally to stress-related behaviors through signaling related to their primary cilia – to do so they would have needed to show that disruption of astrocyte cilia cause stress related behavior. The did causally link astrocyte amygdala S1pr1 to the stress response. Further, the link between S1pr1 and cilia is not clearly articulated in the manuscript except to say that cilia signal with Gpcrs.
4. Related to the point above, it is not clear if the authors are claiming S1pr1 is expressed in cilia, or the alteration of S1pr1 generally in astrocytes is sufficient to rescue alterations observed in cilia.
5. There is gap in logic between the following aspects of the manuscript: 1) Therefore, targeting native, highly expressed astrocyte S1pr1 Gi-GPCRs produced similar restorative effects to those observed with astrocyte Gi-GPCR chemogenetics and the following paragraph 2) Since astrocytes regulate neurons and other cells locally within their territories, we deleted the primary cilium gene Arl13b in amygdala astrocytes (based on methods provided, this was actually all astrocytes) and performed bulk RNAseq to shed light on how gene expression reflecting all amygdala cells was altered.
6. Is S1pr1 gene or protein expression altered in the context of stress in mice or is it changed in human data? Does the ligand change in the context of stress?
7. Can the authors speculate regarding the following statement? 'We found that amygdala astrocytes expressed more cilium-related genes than most other regions evaluated . i.e. what does this mean, are their cilia longer or more numerous?
8. Are the cilia changes restricted to amygdala, or astrocytes? At a minimum this should be discussed, but to support the manuscript, should be quantified.
9. The authors provide evidence that anxiety behaviors in mice can be attenuated through the administration of antidepressants. These experiments distract from the primary goals of the paper, which is determining the effects of stress on amygdala astrocytes. Additional context should be provided as to why these experiments were included. Additionally, it would be useful to see whether cilia are affected by these drugs, considering later in the manuscript poneismod is utilized and similarly attenuates anxiety behaviors.
10. The authors state that S1pr1 is highly specific to astrocytes. However, figure 5d clearly shows high levels of S1pr1 expression in endothelial cells. Additionally, extended data fig 12b shows very little S1pr1 expression in humans. The authors should comment on the potential effects ponesimod may have on endothelial cells, the discrepancies in S1pr1 expression, and how this fits into the context of their study.
11. Please rephrase sentence 1 on page 8 to reflect that Arll3b was deleted in all Aldh1l1+ astrocytes, as well as the sentence in the discussion, 'Genetic disruption of primary cilia by deletion of a key gene (Arl13b) within amygdala astrocytes resulted in non-cell-autonomous effects, perturbing the molecular properties of neighboring parenchymal cells, including neurons.
12. The following statement in not accurate' In this study, we identified a causal contributory role for amygdala astrocytes in

the modulation of stress-related behaviors, mediated via signaling pathways associated with their primary cilia'. This study demonstrated manipulation of amygdala astrocyte S1pr1 was causally linked the modulation of stress-related behaviors. No direct link to cilia was provided.

MINOR CONCERNS
1. "Stress evoked molecular changes…" paragraph should specify the isolation of amygdala astrocyte-specfic RNA-seq
2. The authors utilize DREADDS to target GPCR signaling, which are known to affect baseline characteristics of expressing cells, but do not evaluate baseline differences here. As the authors follow up on and confirm their findings with pharmacological approaches, these experiments are not necessary, but should be acknowledged nonetheless. In general, the authors do not indicate limitations of their study.
3. The authors show that activation of GPCR signaling via deschloroclozapine in DREADD expressing astrocytes in response to stress elevates Ca2+ microdomain firing. Does this return Ca2+ activation to levels seen in unstressed mice?
4. Under "Molecular properties of amygdala astrocytes" authors should specify Rpl22HA Ribotag Mice were crossed with Aldh1l1 specific mice to generate astrocyte specificity.
5. Are there any known off targets of ponesimod? Regardless, this should be acknowledged in the text.
6. Another exciting finding from the authors is that in response to cilia elimination via Arl13b deletion, many key DEGs altered are related to perineuronal nets and perisynaptic extracellular matrix. Have the authors confirmed these changes via IHC through WFA? Providing this not only strengthens the conclusions here, but would provide additional foundations for future studies.
7. Under "Mouse Models", the authors should provide the IACUC protocol numbers relevant to their study
8. Did the authors assess any sex differences?
9. Under "Sucrose preference test (SPT)", authors should include whether animals were returned to group housing following social isolation, as long-term social isolation can affect glial reactivity in the brain.
10. Under "Von Frey test", the word (Ref) is present, and is likely a typo.
11. Under "Immunohistochemistry (IHC)", third paragraph, the authors should include key metrics for their analysis, including smoothing factor, type of thresholding used (absolute or local intensity), and how the threshold value was calculated for both thresholding and segmentation. This would aid in reproducibility of the analysis.
12. Under "analysis of biotinylated proteins using mass spectrometry", the authors can remove the sentence of where the data is deposited, as it is included in the data availability section
13. Did the authors perform any power analysis prior to experimentation to determine sample size, if so, how was this completed and if not, why this was not done.
14. The authors state no data points were excluded from any experiment. Did the authors have any exclusion criteria, such as adverse reactions following surgery, to drugs, etc.?
15. Fig 2i would benefit from visualizing the raw signal to see the effectiveness of segmentation.

Referee #5

(Remarks to the Author)
I co-reviewed this manuscript with one of the reviewers who provided the listed reports.

Version 1:

Reviewer comments:

Referee #1

(Remarks to the Author)
Review of revised manuscript ''Amygdala astrocyte primary cilium mechanisms contribute to aberrant behaviours triggered by stress''.

The authors have done an excellent job incorporating the comments into the manuscript and have significantly improved the manuscript with additional experiments that significantly adds value to the manuscript. We appreciate the rigour the authors have put into addressing our concerns and while the experiments and additional data have addressed some, not all of our questions have been addressed. For instance, the questions around the mechanistic molecular or circuit level link were not addressed experimentally. Two major points that the authors should address are:

Major points:
The authors provided human sequencing data that shows disruption of key cilia genes in neuropsychiatric diseases to provide rationale of relevance. However, the authors report both gene expressions that both increased and decreased in disease and these do not appear to be the same in the genes that were changed in the conditionally knocked out mice or in the stress mouse models. The authors should include an analysis of the overlap between these genes that changed between humans and mice and if there is no overlap, explain/speculate why. Dysregulation of ciliated genes in neuropsychiatric diseases in human in either direction is not obviously causal, thus the authors need to link this back to the causal data obtained from mice where stress causes cilia gene and subsequent length changes or the conditional KO where stress changes and cilia length changes were observed.
Additionally, to underscore the relevance of the sequencing data that revealed cilia-related gene changes to depression, the

authors should include human sequencing data where such changes are not observed for instance in Alzheimer's Disease or Parkinson's Disease.

Referee #2

(Remarks to the Author)
I co-reviewed this manuscript with one of the reviewers who provided the listed reports.

Referee #3

(Remarks to the Author)
The authors have addressed my comments.

Referee #4

(Remarks to the Author)
The technical considerations raised have been addressed. Conceptually, this manuscript reflects substantial effort and presents one of the first thorough descriptions of astrocyte cilia, yet, a clearly contextualized relationship between astrocytic S1pr1 and cilia remains. This should be acknowledged in the discussion.

Consider reducing the lengthy discussion in CRS, WAS, and SNI induce stress-related behavioral phenotypes. These are established stress-inducing models.

To increase clarity, I suggest moving antidepressant experiments to “stress-evoked changes to astrocytic primary cilia”.

Referee #5

(Remarks to the Author)
I co-reviewed this manuscript with one of the reviewers who provided the listed reports.

Open Access This Peer Review File is licensed under a Creative Commons Attribution 4.0 International License, which permits use, sharing, adaptation, distribution and reproduction in any medium or format, as long as you give appropriate credit to the original author(s) and the source, provide a link to the Creative Commons license, and indicate if changes were made.
In cases where reviewers are anonymous, credit should be given to 'Anonymous Referee' and the source.
The images or other third party material in this Peer Review File are included in the article's Creative Commons license, unless indicated otherwise in a credit line to the material. If material is not included in the article's Creative Commons license and your intended use is not permitted by statutory regulation or exceeds the permitted use, you will need to obtain permission directly from the copyright holder.
To view a copy of this license, visit https://creativecommons.org/licenses/by/4.0/

Dear [redacted]

Thank you for the careful review of our manuscript and for the opportunity to revise it. You will see in the point-by-point responses below that we have addressed every Reviewer comment with new data, analyses, and requested revisions. The revisions are marked in blue text.

The revised manuscript now includes six main figures, 18 Extended data figures, and 12 Excel files reporting Source data and additional evaluations of the RNAseq and proteomic data. The title of the manuscript has changed slightly to reflect the request from Reviewers that we do not use the adverb "causally".

In addition, we have formatted the revised manuscript according to your guidelines and included all the requested sections (statistics, reproducibility, data and code availability, methods, ethics statement, extended data, supplementary information, source data and third-party rights). The figures are also formatted according to guidelines.

Kindly note that four new authors have been added to reflect the contents of the revised manuscript and these contributions are stated clearly in the Contributions section and reflect new experiments requested by the Reviewers.

Please let us know if there is something we have missed. With these changes, we hope the manuscript will be considered suitable for publication.

All the best,

[redacted]

Baljit S. Khakh PhD
Professor (on behalf of all authors)
April 4th, 2026

General comments to [redacted] all five Reviewers,

Thank you for the careful review of our manuscript and for the opportunity to revise it. As you will see below, we have addressed every comment raised by the reviewers and editor with new data, analysis, expanded explanations, and revisions. The revised manuscript is much improved because of the comments, and we thank you for your thoughtful guidance and feedback.

In summarizing, the editor wrote that "*While they [the Reviewers] find your work of potential interest, as do we, they have raised important concerns that in our view need to be addressed before we can consider publication in Nature*". We are pleased that the Reviewers and the Editors see the potential interest of our work. We summarize here the major changes to address the comments from all five reviewers. In the subsequent comments to each Reviewer, we cover all points in turn.

1) As requested, we performed several new experiments to be confident that we could image the astrocyte primary cilium and report these data on pages 4-5 with a new results section and accompanying figures. In these experiments, we included new data from two mouse models to genetically impair primary cilia in astrocytes (Arl13b and Ift88 deletions) as validation of the imaging approaches (page 4-5). We also used three different antibody markers and assessed

primary cilia of both astrocytes and neurons, as requested. These data are reported on pages 4-5 and show we can image astrocyte primary cilia reliably – a key point raised by the reviewer 3.

2) We had reported data for major depressive disorder, but Reviewer 1 asked us to explore if astrocyte primary cilia mechanisms were altered in another human psychiatric condition with snRNAseq from postmortem data. We recruited the Steve McCarroll lab as collaborators and reported these data in Fig 5r-u, which shows this was the case for psychosis spectrum disorder. The evidence we provide that astrocyte primary cilia genes are altered in different neuropsychiatric disorders is now very strong (Fig 5) and addresses a key point from reviewer 1.

3) As requested, we disrupted amygdala astrocyte primary cilia genetically (Ift88 mice) and show directly that this alters astrocytic gene expression and is sufficient to cause some stress-related behaviors (page 9). These data support other parts of the manuscript and show that disrupting amygdala astrocyte primary cilia can cause molecular and some behavioral phenotypes that were triggered by stress and reported in earlier figures. This was a key point from reviewers 3 and 4.

4) As requested, we report cohorts of mice across batches (Fig 1 here) to show that the reduction of amygdala primary cilium length was seen in all batches. In fact, the previous submission also included multiple batches, but they were spread across different figures. We added additional batches during the revisions (see Fig 1 in this document), addressing a key point from reviewer 1.

5) As requested, we report new data to show that whereas the primary cilium of amygdala astrocytes decreases in length, that of neurons does not (Fig 2q, t; ED Fig 6). Furthermore, under stress-related conditions the effect was observed for amygdala astrocyte primary cilia but not for astrocytes from the cortex (ED Fig 5). These data show specificity of the effect and provide data to address a key point raised during review by reviewers 1, 3 and 4.

6) As requested, we made many edits to the text throughout to make the flow clearer, to explain the logic and to place our findings into context with the past literature, including commenting on the stress-related models employed. Such clarifications of logic were requested by all reviewers and include an expanded discussion at the request of reviewers 3 and 4.

In addition, we have followed editorial instructions to revise the manuscript according to the detailed formatting guidelines and included all the necessary sections requested (statistics, reproducibility, data and code availability, methods, ethics statement, extended data, supplementary information, source data and third-party rights).

Finally, we have also made changes to the text and figure legends in a traceable way in blue font so you can see what we added. Considering the substantial new data added, the order and calling out of some of the figures has changed and has been checked for accuracy.

Please let us know if there is something we have missed. With these changes, we hope the manuscript will be considered suitable for publication.

Referee #1 (Remarks to the Author):

"In this study, the authors investigate the role of astrocytes in the mouse amygdala in mediating stress-related behaviours. They report a link between ciliated astrocytes in the

amygdala and stress-related behaviours. Upon exposure of mice to distinct paradigms of stress, amygdala astrocytes undergo changes in gene expression and their cilia become shorter. They also show that the GPCR receptor S1PR1, expressed on amygdala astrocytes, when activated with Ponesimod mitigates the effect of stress on cilia length and leads to improved physiological and behavioural changes in mice upon exposure to stress. Although this study provides an interesting link between stress and ciliated astrocytes, the biological significance of the results is not clear and the relevance of these results to human neuropsychiatric disorders is unknown. Moreover, the study lacks sufficient molecular or circuit level mechanistic insights into the link between astrocytes, their cilia, and stress responses in the mouse brain."

We thank the reviewer for the thoughtful summary of our study and for highlighting the novel link between stress and ciliated astrocytes. We appreciate the reviewer's comments regarding the biological significance of these findings and the need for additional context. In the revised manuscript, we have addressed these points with new experiments, analyses, and textual revisions. All additions to the manuscript are indicated in blue.

To address the reviewer's comment regarding biological significance and relevance to human neuropsychiatric disorders, we have incorporated new analyses of new human postmortem amygdala datasets in collaboration with the Steve McCarroll laboratory. Specifically, we examined the expression of primary cilium-associated genes in amygdala astrocytes using single-nucleus RNA-sequencing data from individuals with psychosis spectrum disorder. These new results, now presented in Fig. 5, reveal alterations in astrocytic ciliary gene signatures in this human disease, supporting the translational relevance of our findings. Together with the Phenopedia and postmortem human major depressive disorder analysis already included in the manuscript (Fig. 5), these data substantially strengthen the evidence that astrocyte ciliary pathways identified in our mouse studies are relevant to human neuropsychiatric disorders and are altered in human astrocytes.

We also appreciate the reviewer's suggestion regarding circuit-level mechanisms linking astrocytes, their primary cilia, and stress responses. Our study was intentionally designed to investigate astrocyte-specific mechanisms, an area that has remained largely unexplored in stress biology. We now clarify this rationale in the Introduction (pages 2-3), where we state:

"Our experimental design deliberately prioritized defining astrocyte mechanisms – an approach uniquely positioned to uncover novel astrocyte mechanisms that neuron-centric studies have not yet explored. While synaptic and neuronal circuit mechanisms of stress still require dedicated circuit-mapping approaches for full definition[1-3], the astrocyte-focused findings we report provide essential molecular entry points to meaningfully assess astrocyte contributions to those circuits moving forward. Accordingly, based on our findings, we formulated and tested hypotheses centered on astrocytes and their primary cilia (**Fig. 1a**)."

We have also added additional discussion (page 12) outlining how the present findings enable future circuit-level investigations:

"Moreover, although electrophysiological studies of how astrocyte cilia influence neural circuits do not yet exist, our findings and tools now enable such circuit-level dissection. Detailed mapping of stress-related neuronal circuit dynamics, studies of which are incomplete[1-3], downstream of astrocyte

ciliary signaling, will require complementary approaches including brain slice and *in vivo* electrophysiology. These represent important directions for much future work."

Finally, we fully agree that defining the circuit-level consequences of astrocyte ciliary signaling represents an important next step. Such experiments will require extensive in vivo electrophysiological and circuit-mapping approaches in multiple mouse models that extend beyond the scope of the present study and will likely take several years. Such work would represent a specific manuscript. We have therefore clarified this point in the revised manuscript and outline these directions as priorities for future work (see text in blue above).

We hope the reviewer agrees that the additional human data, expanded discussion, and clarification of the study's scope substantially strengthen the manuscript and address these concerns. In addition, all the other comments are addressed in turn below.

Additional Major Concerns

1. "*What is the level of change in primary cilia length that would be biologically meaningful? In this study, the change in cilia length in amygdala astrocytes does not appear robust. For example, the changes shown in Figure 1 appear rather modest with large variation. Also, it is not clear whether and how the study was powered to detect biologically meaningful changes in primary cilia length*"

We thank the reviewer for raising these important points regarding the biological interpretation and robustness of the observed changes in primary cilium length.

Regarding the magnitude of change, there is currently no established consensus defining a specific threshold at which alterations in primary cilium length become biologically meaningful. However, numerous studies have demonstrated that the primary cilium is a highly dynamic organelle in which relatively modest increases or decreases in length can significantly influence ciliary signaling and downstream cellular function. To clarify this point, we have added the following text to the revised manuscript, which also includes discussion of points requested by reviewer 2 (page 11):

"Although a precise threshold for functionally meaningful changes in primary cilium length has not been established, the primary cilium is recognized as a morphologically dynamic organelle in which even modest alterations in length, either increases or decreases, can influence signaling and cellular function[4-9]. In addition to altering the amount, spatial distribution, or effective density of signaling components within the cilium, changes in ciliary length may influence cellular signaling through several other mechanisms[8,10]. Shortening of the primary cilium may reduce the available membrane surface for receptor localization, potentially affecting signal sensitivity to extracellular ligands. It may also influence intraflagellar transport dynamics, thereby altering the trafficking, delivery, or turnover of signaling proteins within the ciliary compartment. Changes in length could additionally modify the spatial organization of sub-ciliary domains (e.g., base, shaft, and tip)[8,10], potentially affecting interactions among receptors and scaffolding proteins. Because the primary cilium can function as a mechanosensor, shorter cilia may also alter the mechanical properties of the organelle and its ability to transduce physical stimuli. Finally, cilium length may influence cellular states linked to ciliary dynamics, such as quiescence, all of which could contribute to altered downstream signaling outcomes. These possibilities may contribute to the changes reported in this study, but we are aware of no cases where these mechanisms have been teased apart and are not aware of genetic tools to do so systematically – reflecting

the fact that exploring primary cilium biology *in vivo* is a nascent area of biology. Under normal homeostatic conditions, critical functions would be served by primary cilia around 4-5 µm in length, but such functions would change with shorter primary cilia. Consistent with a role for primary cilia in neurobiological responses, genetic ciliopathies frequently include neurological manifestations[10], supporting the view that ciliary perturbations can affect brain function. Together, our findings indicate that stress induces coordinated changes in cilium-associated genes, proteins and morphology in amygdala astrocytes, consistent with a role for astrocytic ciliary alterations in stress-related pathology. Further mechanistic studies are now needed to unlock the signaling and biophysics of the primary cilium."

We also appreciate the opportunity to clarify the robustness of the observed effect. The experiments shown in the original manuscript were performed across multiple independent experimental batches that were presented in different figures depending on the associated analyses. Below we now summarize these data across six independent experimental batches and include pooled analyses in the last panel (Fig 1 here). In each batch, stress exposure produced a consistent and statistically significant reduction in astrocyte primary cilium length, and the same effect is observed in the pooled dataset. These clarifications further demonstrate the reproducibility of the findings.

In addition, to provide a clearer visualization of the distribution of measurements in the figures, we now include histograms of primary cilium length in the revised figures (Fig 2r, ED Fig 5, 6, 7), which reveal a consistent shift in the modal distribution following stress.

With respect to statistical power, these experiments were not initially designed to detect changes in cilium length, as this phenotype was an unexpected finding and emerged during our analyses. Subsequent experiments were therefore performed in independent cohorts of ~4 mice per group per batch, which was the practical cohort size that could be processed simultaneously for consistent tissue preparation and imaging. Across these independent cohorts, the reduction in astrocyte primary cilium length was reproducible and statistically significant, as mentioned above.

Finally, in response to this and other reviewer comments (especially Reviewer 3), we performed additional validation experiments to further support the robustness of the measurements. These experiments, now described on pages 4-5, include: **(i)** measurements using three independent ciliary antibody markers, **(ii)** genetic disruption of astrocytic primary cilia through Arl13b and Ift88 deletion, and **(iii)** comparative analyses across astrocytes and neurons as well as in amygdala and cortex. These analyses confirmed significant reductions in primary cilium length specifically in amygdala astrocytes, whereas neuronal cilia and astrocyte cilia in cortex did not show comparable changes. The specificity of the effect also supports the robustness of the phenomenon and its measurement.

Together, these additional analyses and experiments strengthen the robustness and interpretation of the observed stress-associated changes in astrocyte primary cilium length, and we sincerely thank the reviewer for calling for these clarifications and new experiments. These additions have made our work stronger, and we greatly appreciate the guidance.

2. "*What is the relevance of the distinct paradigms used in this study in human neuropsychiatric disorders? In other words, what is the predictive validity of finding a change in primary ciliary length in such a paradigm?*"

We thank the reviewer for raising the important question of how the paradigms used in this study relate to human neuropsychiatric disorders and the predictive validity of the observed ciliary changes. We have clarified this point throughout the revised manuscript.

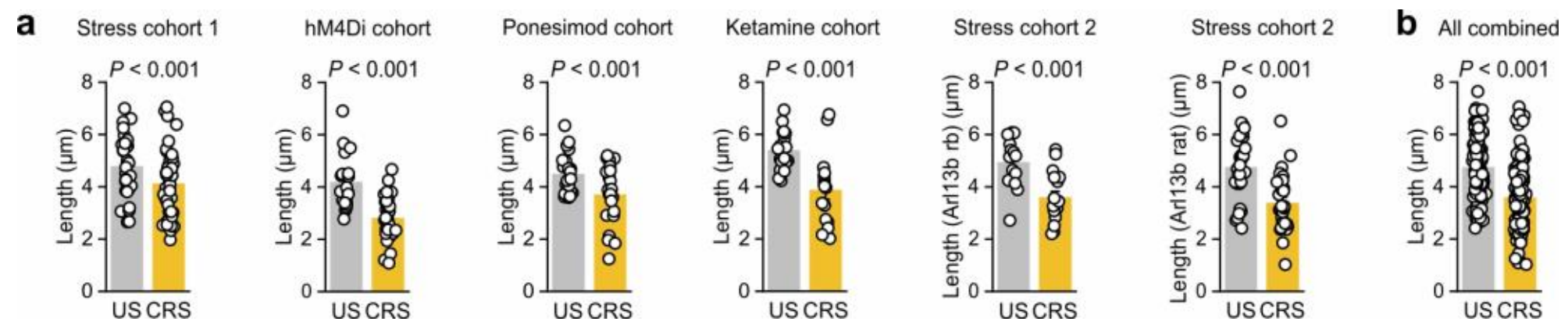

Fig 1. Robustness of reduction in primary cilium length after CRS. a, Cilium length of BLA astrocytes from US and CRS mice from different cohorts, for our most comprehensive data. All measurements were based on IHC staining of Arl13b (rabbit antibody) except the last panel which is based on staining with a rat Arl13b antibody. Stress cohort 1: 33 astrocytes from 4 US mice, 45 astrocytes from 4 CRS mice; hM4Di cohort: 20 astrocytes from 4 US mice, 34 astrocytes from 4 CRS mice; ponesimod cohort: 23 astrocytes from 4 US mice, 23 astrocytes from 4 CRS mice; ketamine cohort: 18 astrocytes from 4 US mice, 16 astrocytes from 4 mice; stress cohort 2 (Arl13b rb antibody): 14 astrocytes from 4 US mice, 17 astrocytes from 4 mice; stress cohort 2 (Arl13b rat antibody): 30 astrocytes from 4 US mice, 27 astrocytes from 4 mice. **b,** Combined data for all cohorts. Bars represent the mean and error bars (sometimes obscured by the data points) represent the standard error of the mean. Normal distribution was determined using OriginPro 2024. If the data were normally distributed, an unpaired Student's two-tailed t-test was performed; if they were not normally distributed, a two-tailed Mann-Whitney test was performed.

More specifically, regarding the relevance of the experimental paradigms, the stress models used in this study - chronic restraint stress (CRS) and water aversion stress (WAS) - are widely used and well-characterized approaches for inducing physiological and behavioral phenotypes that parallel core features of human anxiety and mood-related disorders (additional key references are now cited in the text on page 2). In our experiments, both paradigms produced robust and reproducible physiological and behavioral changes consistent with stress-associated pathology. These included reductions in body and thymus weight, increased adrenal weight and circulating corticosterone levels, as well as behavioral alterations such as increased anxiety-like behavior (open field test), anhedonia (sucrose preference test), and depression-like behavior (forced swim test). These phenotypes represent widely used translational readouts relevant to human conditions including major depression, stress, anxiety disorders, bipolar disorder, and schizophrenia. We also initially evaluated a chronic pain model (spared nerve injury, SNI). Although this paradigm produced the expected hypersensitivity to pain, it did not reproduce the stress-associated physiological and behavioral alterations described above and was therefore not pursued further in this study.

Key references to the stress models and their value are provided in the revised manuscript. However, importantly, the goal of the stress paradigms used here is not to fully model complex human psychiatric disorders, but rather to reproduce key neurobiological consequences of chronic stress that are strongly implicated in their underlying pathophysiology. Within this framework, these models provide a tractable system to investigate cellular and molecular mechanisms through which chronic stress may affect brain function.

To address the relevance of primary cilium alterations in human neuropsychiatric disease, we examined independent human astrocyte transcriptomic datasets. In previously published single-nucleus RNA sequencing data from individuals with major depressive disorder (MDD), cilium-related genes were found by us to be robustly expressed in human astrocytes and multiple cilium-associated genes were differentially expressed across all astrocyte clusters compared to neurotypical controls (Fig. 5o-q). To further explore this question, we established a collaboration that enabled the inclusion of additional new human data in the revised manuscript. Specifically, we analyzed RNA-seq data from basolateral amygdala (BLA) astrocytes obtained from individuals with psychosis spectrum disorder, including bipolar disorder and schizophrenia, and compared them with unaffected controls. In this dataset, 74 cilium-related genes were differentially expressed in PSD astrocytes from the 457 cilium-associated genes detected in the experiment (Fig. 5t-u).

Together, these human datasets indicate that cilium-related molecular pathways are altered in astrocytes across multiple neuropsychiatric disorders. These findings converge with our mouse data, in which stress paradigms that model key aspects of stress-related pathology produce structural alterations in astrocyte primary cilia. Notably, a pharmacological intervention used in humans (ketamine) that rescued stress-induced behavioral phenotypes also restored primary cilium length (Page 5; ED Fig 7). The convergence between human transcriptomic alterations in ciliary pathways and the stress-induced structural ciliary phenotype observed in our mouse models supports the relevance of astrocyte primary cilium biology to stress-related neuropsychiatric disease and provides a rationale for further investigation.

All the preceding points have been clarified throughout the revised manuscript, and we thank the reviewer for prompting us to do so.

3. "*Are the changes in cilia genes and cilia length in mice exposed to stress specific to astrocytes or do the changes also occur in other cell types such as excitatory neurons or inhibitory interneurons? The authors should consider investigating the transcriptomic changes in their mouse models with single cell sequencing to evaluate how cilia gene changes in astrocytes impact the transcriptome of the other cell types that interact with astrocytes.*"

We thank the reviewer for this insightful comment. To address whether stress-induced changes in primary cilia are specific to astrocytes or also occur in other cell types, we performed additional analyses examining cilia length in both astrocytes and neurons. In the amygdala, the reduction in primary cilium length following stress was observed in astrocytes but not in neurons (Fig. 2q, t). We also conducted similar analyses in the cortex and found that primary cilium length was not altered in either astrocytes or neurons in this region (ED Fig 5 and 6). These new data directly address the reviewer's question and are now included on page 5.

With respect to the reviewer's suggestion regarding single-cell RNA sequencing, we considered this approach to evaluate how astrocyte cilia disruption might influence other cell types in the amygdala. However, because the mouse amygdala has been comprehensively characterized at the single-cell level in recent work[11], we instead leveraged these published datasets – which is in accord with the purpose and availability of those atlas-type studies. Specifically, we used established cell-enriched and cell type-defining gene signatures derived from these single-cell data[11] and mapped them onto our bulk RNA-seq datasets to infer how different amygdala cell populations are affected by astrocyte primary cilia gene disruption.

These analyses indicate that multiple amygdala cell populations, including neurons, exhibit transcriptional changes and are presented in Fig. 5d-f of the manuscript (also ED Fig 18).

Finally, we note that although single-cell RNA sequencing is a powerful approach for cell classification, our study was designed to use deep RNA sequencing with the RiboTag method. This strategy provides substantially greater sequencing depth and enables detection of a larger number of transcripts that are being translated by the ribosome[12,13], while also overcoming the relatively low capture rate of astrocytes in single-cell protocols[14]. For these reasons, we prioritized RiboTag-based profiling using fully validated genetic mouse models[12,15-17]. We agree that future studies using increasingly sensitive single cell approaches that capture relevant numbers of astrocytes may further expand on these findings.

4. *"The authors do not provide a mechanistic molecular or circuit level link of how deregulation of primary cilia length might lead to functional changes that cause a behavioural outcome. In the absence of such insights, the study is premature and the claim that targeting astrocytic primary cilia might provide a novel treatment strategy for stress-related conditions is an overstatement".*

We appreciate the reviewer's suggestion regarding circuit-level mechanisms linking astrocytes, their primary cilia, and stress responses. We addressed this on page 3 above and kindly point the reviewer to that response.

In retrospect, we agree that we overstated the clinical relevance of the primary cilium findings and have now toned down those aspects and focused the study on the interesting cell biology and the potential translational relevance. On the other hand, reviewer 4 asked for additions that were related to translational relevance and so we had to strike a balance between comments from reviewer 1 and 4. We thank the reviewer for this feedback and would be happy to work with the editor to make sure we get the balance right between reviewers' feedback.

5. *"Ponesimod is a specific agonist of the S1PR; however, in effect it acts as a functional antagonist (Kruger et al., 2023). Upon binding to the S1PR, it leads to internalisation of the receptor therefore acting as a functional antagonist and reduction in overall S1P signalling in the periphery. The authors should characterize how ponesimod activity on the S1PR on astrocytes links to changes in cilia. In addition, is expression of S1P or S1PR1 deregulated in the amygdala of patients with neuropsychiatric disorders?"*

We thank the reviewer for these helpful comments.

Ponesimod has been described as a functional antagonist of S1PR1 in peripheral lymphocytes, where ligand-induced receptor internalization suppresses S1PR1 signaling and prevents lymphocyte egress from lymphoid organs (Kruger et al., 2023). To evaluate whether similar functional antagonism occurs in astrocytes, we examined calcium signaling as a functional readout of S1PR1 activation. In acute brain slices, bath application of ponesimod induced an increase in calcium activity in astrocytes (ED Fig 13d-f). To further test whether in-vivo ponesimod exposure induces receptor internalization and functional antagonism, we performed calcium imaging in BLA astrocytes in brain slices prepared after intraperitoneal injection of ponesimod (1 hr, 30 mg/kg) or vehicle. If ponesimod induced functional antagonism in astrocytes, we would expect a diminished calcium response to a second ponesimod

application. However, bath application of ponesimod produced similar increases in both the amplitude and frequency of calcium signals in astrocytes from vehicle- and ponesimod-treated mice (ED Fig 13g-h). These data suggest that under these conditions ponesimod does not produce functional antagonism of S1PR1 signaling in astrocytes.

Regarding the link between S1pr1 and the primary cilium, we appreciate the opportunity to clarify this point. We did not intend to suggest that S1pr1 localizes to the primary cilium. New immunohistochemical analysis of S1pr1 in astrocytes did not reveal ciliary localization (ED Fig 14b). Consistent with this, our new proximity-dependent in vivo primary cilium proteomics dataset using ARL13B in the cortex (Fig. 6) did not identify S1pr1 in the ciliary proteome either, whereas it was detected in plasma membrane proteome (Fig 4a-c). In accord with these data, the CilioGenics, CiliaCarta, PCPD and Syscilia databases of primary cilium genes and proteins also do not identify S1pr1 as being within the primary cilium. In the revised manuscript, we have clarified that our findings support a model in which S1pr1 acts from the plasma membrane rather than from the primary cilium (see pages 11 and 12). Future detailed genetic studies will be required to define the molecular mechanisms linking S1pr1 activation at the plasma membrane to downstream effects on primary cilium signaling.

Finally, we examined whether S1PR1 expression is altered in neuropsychiatric disorders using available human single-cell RNA-seq datasets. Although S1PR1 is found in human amygdala astrocytes (Fig 4), it was not identified as a differentially expressed gene in these datasets. We have now included this information in the manuscript (page 10). This observation is consistent with our mouse RNA-seq and proteomic data where S1pr1 expression did not change following stress (Fig 4c) and suggests that S1PR1 signaling pathways remain intact, potentially supporting the therapeutic relevance of targeting this pathway – although we have toned that aspect down because of the previous comment from reviewer 1.

<u>Minor Comments:</u>

1. *"The authors describe chronic restraint stress (CRS) as having the more robust phenotype but the cilium length change was less significant than with water avoidance stress (WAS) (p<0.05 vs p<0.005), and the volume having a greater change in CRS than in WAS, are the differentially expressed cilium genes able to account for this morphological difference? Figure 2j."*

We thank the reviewer for the opportunity to clarify these points.

<u>First,</u> the p-values reported in the manuscript reflect the probability associated with the statistical test rather than the magnitude of the effect. Therefore, the difference between $p < 0.05$ and $p < 0.005$ should not be interpreted as indicating that one stress paradigm produces a larger biological effect than the other.

<u>Second,</u> the comment appears to conflate two separate findings in the manuscript. CRS produced a more robust behavioral phenotype compared with WAS (Fig 1d-f). However, when examining primary cilia morphology, CRS and WAS both resulted in comparable reductions in astrocyte cilium length and volume relative to control animals (Fig 2q, s). Therefore, we did not observe a significant difference between the magnitude of cilium shortening induced by CRS versus WAS. Consistent with these morphological findings, analysis of cilium-related transcriptional pathways revealed parallel changes in both stress paradigms (ED Fig 2d).

Because the reduction in cilium length was similar between CRS and WAS, our data do not indicate a differential morphological phenotype that would require explanation by distinct transcriptional mechanisms.

We have revised the manuscript to clarify the distinction between behavioral outcomes and ciliary morphological changes and to avoid potential confusion regarding the interpretation of p-values.

2. "*Can the authors detect a reduction in GFAP in the CSF as they report a reduction in GFAP+ cells in the amygdala with CRS and WAS? Figure 1h.*"

We thank the reviewer for this suggestion. We believe there may be a slight misunderstanding regarding our findings. In the manuscript, we report a reduction in the number of GFAP-positive astrocytes, but we did not observe a change in the total number of astrocytes (Fig 1j-l). Thus, our data indicate a decrease in GFAP expression rather than astrocyte loss.

In most clinical and experimental contexts, elevated GFAP levels in cerebrospinal fluid (CSF) are associated with astrocyte injury, hypertrophy, or cell death, such as during trauma or neurodegenerative disease. In our stress models (CRS and WAS), we do not observe evidence of astrocyte loss or neurodegeneration. Instead, the data indicate a reduction in GFAP expression within astrocytes in the amygdala. Because the total number of astrocytes remains unchanged and GFAP expression is reduced rather than increased, we would not expect a detectable increase in CSF GFAP levels. Moreover, CSF GFAP measurements typically reflect large-scale astrocytic injury across substantial brain regions. Detecting subtle reductions in GFAP expression originating from a single brain region, such as the amygdala without any loss of astrocytes, would likely be below the sensitivity of current CSF assays. For these reasons, we do not believe that measuring CSF GFAP would provide an informative readout in our CRS or WAS models.

3. *"Image 1h shows a reduction in GFAP intensity rather than a reduction in the number of GFAP+ cells in the nuclei."*

We thank the reviewer for this comment. We believe there may be a slight misunderstanding. GFAP is an intermediate filament protein localized to the astrocytic cytoskeleton and is not present in the nucleus; accordingly, we did not quantify "GFAP+ nuclei." Instead, we first quantified the total number of astrocytes using the well-established astrocyte nuclear marker Sox9. We then determined the proportion of these Sox9+ astrocytes that were GFAP-positive. Using this approach, we observed that stress reduced the proportion of Sox9+ astrocytes that were GFAP+ (Fig 1l). We have revised the text to clarify this analysis and avoid confusion.

4. *"Can the authors speculate why CRS and WAS produced cilium genes that were unique given that the significantly changed biological processes (Extended Figure 2e) were by and large the same but have different significance? Was the net outcome the same despite these unique gene differences?"*

We agree with the reviewer's interpretation. Biological processes and pathways are typically supported by multiple, partially overlapping gene sets, and disruptions to the same pathway can therefore arise through changes in different individual genes. In our dataset, CRS and WAS each produced somewhat distinct sets of differentially expressed cilium-related genes, but these gene changes converged on largely overlapping biological processes and pathways (ED Fig 2d). Thus, despite differences at the level of individual genes, the overall functional outcome at the pathway level was largely similar between the two stress paradigms, as the Reviewer also concluded.

5. *"The authors could consider moving the molecular properties of amygdala astrocytes to the start of the paper."*

We appreciate this thoughtful suggestion. We considered restructuring the manuscript accordingly. However, after implementing revisions requested by other reviewers and reassessing the overall flow and logic of the manuscript, we found that these data fit most logically in Fig. 5 of the revised manuscript. In this position, they provide important context by demonstrating that the transcriptomic profile of amygdala astrocytes differs from astrocytes in other brain regions, which helps frame the subsequent molecular findings presented in the study.

6. *"Error bars should be defined in the figure legends."*

We have included definitions of error bars in the figure legends.

Reference:

1. Kruger, T.M., Valenzuela, B., Thompson, C.D., Ouwerkerk-Mahadevan, S., and Ruixo, J.J.P. (2023). Clinical Pharmacokinetics of Ponesimod, a Selective S1P1 Receptor Modulator, in the Treatment of Multiple Sclerosis. Clin. Pharmacokinet. 62, 1533–1550. https://doi.org/10.1007/s40262-023-01308-5.

Referee #2 (Remarks to the Author):

"I co-reviewed this manuscript with one of the reviewers who provided the listed reports."

Thank you. Your points are all addressed in responses to Referee 1.

Referee #3 (Remarks to the Author):

"*The molecular mechanisms linking exposure to stress and aberrant behavior are still mostly unknown. Understanding these mechanisms is important for our ability to treat multiple psychiatric disorders. Pelaz et al. investigated the mechanisms by which amygdala astrocytes influence stress-related behavior. The authors mice to various stress models such as chronic restraint stress (CRS) or water aversion stress (WAS), to induce stress-related behavior. These stressful conditions evoked transcriptional and proteomic responses in amygdala astrocytes. Using immunohistochemistry, the authors detected a reduction in the length and volume of primary cilia in amygdala astrocytes following stress. Moreover, given that importance of GPCR signaling in primary cilia signaling, the authors evaluated the ability of DREADD-driven Gi activation (hM4Di) to rescue stress-evoked behavioral deficits. hM4Di activation was sufficient to rescue the stress-evoked behavioral deficit and length reduction of primary cilia.*

Finally, based on their finding that the modulation of GPCR signaling is beneficial in the context of stress-related behavior, the authors evaluated the potential therapeutic effect of activating the astrocyte-expressed S1PR GPCR with the clinical-grade agonist Ponesimod, finding that it rescues the behavioral deficits induced by stress.

Given the significant impact of stress on global health in modern society, studying stress-induced behavioral changes and the mechanisms responsible for them is timely. In addition, this study is particularly exciting because as it identifies contributions of amygdala astrocytes to stress-evoked behavioral deficits. Indeed, linking astrocytic primary cilia to behavioral output is highly novel and original. Moreover, the beneficial effects of Ponesimod, a drug already in clinical use, open unexpected therapeutic avenues. The techniques used are cutting-edge, and the data produced is informative and resourceful. The authors are leaders in the field, and the studies are properly controlled and described. However, I believe that some issues have to be addressed before this work is ready for publication."

We thank the reviewer for the supportive comments regarding the timeliness, the novelty and the originality of the study, as well as the positive comments regarding the quality and suitability of the techniques and the generated data. We also appreciate that they recognized our past work. We are humbled by this support.

We have now addressed the thoughtful and helpful comments raised by the reviewer with new data, analyses and revisions. Kindly see the responses to the following points.

Major comments

1. *"Given that cilia are very small structures (~4 µm in length), it would be helpful if the authors could further justify the use of immunohistochemistry for their visualization. Alternatively, reproducing the key findings with methods such as electron microscopy could further strengthen the impact of the study."*

Thank you for this suggestion, which we addressed with a new section of the manuscript reporting new experiments on pages 4-5. Thus, in response to this and other reviewer comments, we performed additional validation experiments to further support the measurements. These experiments, now described on pages 4-5, include: **(i)** measurements using three independent ciliary antibody markers, **(ii)** genetic disruption of astrocytic primary cilia through Arl13b and Ift88 deletion, and **(iii)** comparative analyses across astrocytes and neurons and in amygdala and cortex. These analyses confirmed significant reductions in primary cilium length specifically in amygdala astrocytes, whereas neuronal cilia and astrocyte cilia in cortex did not show comparable changes. The specificity of the effect also supports the robustness of the phenomenon and its measurement. The primary cilium genetic disruption experiments showed specificity of the immunostaining methods used to measure cilia. Overall, these new experiments provide the requested justification.

On pages 4-5 we write:

"Amygdala astrocytes possess primary cilia

To determine if changes in cilium-related gene and protein expression following CRS and WAS (**Fig. 2a-g**) affected astrocyte primary cilia, we performed IHC using the cilium-enriched markers Arl13b and AC3[18-21]. We started with validations with these markers. We identified Arl13b-positive primary cilia emerging from S100β-positive amygdala astrocytes (**Fig. 2h, Extended data Fig. 4a**). Consistent with past studies[22], the primary cilium marker AC3 was located within the same structures but with patterns dissimilar to Arl13b. This was apparent from line profiles (**Fig. 2i**), areas of Arl13b and AC3 staining

(**Fig. 2j**), and their overlap (**Fig. 2k**). The more uniform expression of Arl13b allowed us to estimate the length of astrocyte primary cilia at ~5 µm, consistent with previous reports[22]. To further validate IHC to image astrocytic primary cilia, we selectively disrupted amygdala astrocyte primary cilia in *Arl13b^f/f^* and *Ift88^f/f^* mice[23] with astrocyte-specific Cre AAVs in relation to control tdTomato AAVs (tdT; **Fig. 2m**). Arl13b is a small GTPase whereas Ift88 is a core protein of the intraflagellar transport machinery that traffics proteins in and out of the cilium; both are required for ciliary signaling and genetic reduction of either causes cilial disruption[23]. Arl13b and AC3 immunostaining was observed in S100β-positive astrocytes from tdT mice, but not from Cre mice (**Fig. 2n**). In accord, the proportion of astrocytes with primary cilia dropped from >80% to ~10% in Ift88 and Arl13b deletion mice (**Fig. 2o; Extended data Fig. 4b-c;** IHC with rabbit and rat Arl13b antibodies produced similar findings). In addition, we examined by RNAscope the expression of two known cilium genes (*Arl3* and *Arl6*) and detected expression in ~95% of amygdala S100β-positive astrocytes (**Extended data Fig. 4d, e**). Furthermore, primary cilium related genes, including *Arl13b* and *Ift88*, were abundantly expressed in human postmortem amygdala astrocytes from published data[24] (**Extended data Fig. 4f**). Our validation studies with three antibody markers (rat Arl13b, rabbit Arl13b, AC3) in control mice and those with genetic disruption of astrocytic primary cilia (Arl13b and Ift88 deletions), as well as evaluations of gene expression in mice and humans (**Fig. 2h-o**; **Extended data Fig. 4a-f**), provide strong evidence that amygdala astrocytes possess primary cilia and that we could measure them with IHC."

2. "*I am curious about the biological significance of cilia shortening. From a signaling perspective, how does this change affect cellular function, particularly in astrocytes, and what would be expected under homeostatic conditions? Adding such a discussion to the manuscript would provide valuable context and make the paper even more engaging for readers.*"

Thank you. This was a great suggestion for additional discussion. We added the following section to the discussion on page 11.

"Although a precise threshold for functionally meaningful changes in primary cilium length has not been established, the primary cilium is recognized as a morphologically dynamic organelle in which even modest alterations in length, either increases or decreases, can influence signaling and cellular function[4-9]. In addition to altering the amount, spatial distribution, or effective density of signaling components within the cilium, changes in ciliary length may influence cellular signaling through several other mechanisms[8,10]. Shortening of the primary cilium may reduce the available membrane surface for receptor localization, potentially affecting signal sensitivity to extracellular ligands. It may also influence intraflagellar transport dynamics, thereby altering the trafficking, delivery, or turnover of signaling proteins within the ciliary compartment. Changes in length could additionally modify the spatial organization of sub-ciliary domains (e.g., base, shaft, and tip)[8,10], potentially affecting interactions among receptors and scaffolding proteins. Because the primary cilium can function as a mechanosensor, shorter cilia may also alter the mechanical properties of the organelle and its ability to transduce physical stimuli. Finally, cilium length may influence cellular states linked to ciliary dynamics, such as quiescence, all of which could contribute to altered downstream signaling outcomes. These possibilities may contribute to the changes reported in this study, but we are aware of no cases where these mechanisms have been teased apart and are not aware of genetic tools to do so systematically – reflecting the fact that exploring primary cilium biology *in vivo* is a nascent area of biology. Under normal homeostatic conditions, critical functions would be served by primary cilia around 4-5 µm in length, but such functions would change with shorter primary cilia. Consistent with a role for primary cilia in neurobiological responses, genetic ciliopathies frequently include neurological manifestations[10], supporting the view that ciliary perturbations can affect brain function. Together, our findings indicate

that stress induces coordinated changes in cilium-associated genes, proteins and morphology in amygdala astrocytes, consistent with a role for astrocytic ciliary alterations in stress-related pathology. Further mechanistic studies are now needed to unlock the signaling and biophysics of the primary cilium."

3. *"It would be helpful if the authors could better explain the transition from astrocyte cilia studies to GPCRs. While it is true that GPCR signaling is important in cilia, GPCRs are also broadly critical in many other contexts. Please expand the justification on why the study moves from cilia to GPCRs."*

Thank you for requesting this clarification. We have revised the manuscript to better explain the conceptual transition from astrocyte cilia to GPCR signaling and to make the rationale more explicit in the relevant section (page 6). Specifically, we added the following text:

"GPCRs are known to regulate multiple aspects of astrocyte morphology[25] and signaling[26]. Because we observed reduced astrocyte primary cilium lengths, we next considered signaling pathways that are strongly associated with this organelle. Primary cilia are well-established hubs for GPCR-mediated signaling[27], and GPCR pathways also play key roles in astrocyte function, including within the amygdala[28]. Based on this overlap, we analyzed our proteomic datasets (**Fig. 2d-f**) from US, CRS, and WAS groups to identify GPCRs expressed in amygdala astrocytes and to determine their predicted G-protein coupling[29,30] (**Fig. 3a-c**). Based on these data we hypothesized that activation of GPCR signaling pathways might influence primary cilium morphology. Among the GPCRs detected in astrocytes by proteomics, most were predicted to couple to Gi and/or Gq proteins[31-34] (**Fig. 3a-c**; e.g., Gi-coupled receptors S1pr1, Fzd2, Grm2, Grm3; Gq-coupled receptors Grm5, Grm1, Sstr2, Chrm3), which led us to test whether astrocyte-specific activation of GPCR signaling[28-30,35] pathways could influence stress-evoked behaviors."

4. *"In the stress-induced model, Gi-DREADD activation restores cilia length and rescues behavior. However, to establish a stronger link and exclude the potential contribution of other brain regions, it would be valuable to examine how AAV-driven Arl13b inactivation in amygdala astrocytes affects behavior and/or neuronal circuit function."*

This was an excellent suggestion, which we addressed on page 9 with the following section with new experiments by deleting Ift88 – another primary cilium gene – the deletion of which we validated most extensively with three different antibodies (Fig 2m-o).

"Next, we used our AAV strategy to disrupt astrocyte primary cilia only in the amygdala in adult mice (**Fig. 2m**) and performed astrocyte-specific RNAseq with MACS[36] (**Fig. 5h-l**; **Extended data Fig. 17b-j**). Arl13b and Ift88 deletion from amygdala astrocytes resulted in 142 and 129 DEGs, respectively (**Fig. 5h-l**). In both cases, the key pathways were glial reactivity, extracellular matrix (ECM), cilium biology and collagen containing ECM (**Fig. 5j, l**). We validated collagen with IHC and found that it was decreased, and we validated perineuronal nets known to be replete with ECM components with WFA and found a significant increase (**Extended data Fig. 17k-n**) – both these changes were in accord with RNAseq (**Fig. 5d-l**). Furthermore, astrocyte reactivity genes were mostly down regulated (**Extended data Fig. 17j**), which is consistent with the lower levels of GFAP following stress (**Fig. 1k, l**). Next, we performed behavior on mice in which amygdala astrocyte Ift88 was deleted and was assessed with three markers (**Fig. 2o**) and RNAseq (**Fig. 5k**). We found significant deficits in stress-related behaviors measured by OFT, but not in those measured by SPT and FST (**Fig. 5m**; 16-17 mice per group). Overall, these data provide strong evidence that loss of primary cilia within amygdala astrocytes alone can alter astrocytes, surrounding cells, and lead to some stress-related behaviors. These data imply that amygdala

astrocyte primary cilia signaling contributes markedly to anxiety states, in accord with recent work[37], whereas other stress-related behaviors (**Fig. 1**) require convergence of additional changes evoked by CRS and WAS."

5. *"Since much of the work focuses on manipulating and staining for Arl13b, it would be helpful if the authors could clarify why this target was selected. Does Arl13b detected in downregulated cilia-related pathways?"*

Thank you for this helpful comment. Arl13b was not downregulated in any of our RNA-seq or proteomics datasets, except in the *Arl13b* f/f MACS RNA-seq dataset where its reduction was expected due to genetic deletion. We selected Arl13b primarily because it is currently one of the most robust and widely used markers for labeling primary cilia, including in astrocytes[23]. The data we show on pages 4-5 also supports the reliability of this marker and therefore provides the requested clarification for its use. But please do also note in the preceding comment we expanded to use Ift88 deletions as well, and we used AC3 staining as an additional metric. Therefore, our findings are not dependent on just Arl13b deletion or Arl13b staining.

<u>Minor comments</u>

1. *"In ED Fig. 4a, Arl13b immunoreactivity is a bit dim, and overlay panels can be visually improved for the readers."*

We have improved the images and added more examples in the main figures and in the extended data.

2. *"I cannot find ED Fig. 8a in the main manuscript"*

We carefully checked and although the figure order has changed due to the revisions, all the figures are now cited in the main manuscript.

3. *"Could you please discuss downregulated astrocyte DEGs in Fig. 2b?"*

Down-regulated astrocyte-enriched DEGs shown in Fig. 2b refer to DEGs from the IP fraction (i.e., genes corresponding to mRNA isolated from astrocyte ribosomes) that are down-regulated in CRS or WAS and also satisfy the condition of being astrocyte enriched by having a $\log_2$ fold change over the input fraction (RNA from all cells) > 1 with an adj. *P*-value < 0.05. This does not imply a loss of astrocytic phenotype, but it does indicate that genes that were highly enriched in astrocytes compared to other cell types in the controls were down-regulated in CRS or WAS. We have clarified this in the legend for Fig 2b.

4. *"The authors may want to move some non-significant data from main Figs to Extended Data Figs to bring focus on the significant differences detected."*

Thank you. We have moved some of the panels showing statistically non-significant changes to Extended Data to focus on the differences. The figure order has changed in revision, however, to reflect the new data we added.

5. *"Making the subheadings more specific could the readability of the manuscript."*

Thank you. We have improved subheadings to make them more specific.

Referee #4 (Remarks to the Author):

"In the current study, Pelaz et al. present findings that amygdala astrocyte GPCRs, specifically S1pr1, contribute casually to molecular and behavioral phenotypes in response to stress. The authors also find that in response to chronic stressors, astrocyte cilia present as smaller and correlate with increased anxiety like behavior, while RNA sequencing of astrocytes demonstrates dysregulated gene expression, including those related to cilia. Near-proteomic approaches, also identify cilium related proteins. Chemogenetic targeting of GPCRs and pharmacological modification of S1pr1 rescued anxiety like behavior and astrocyte cilia changes. Human RNAscope and RNA-seq data also demonstrates alterations in cilia related genes. Finally, the authors demonstrate knocking out the cilia protein Arll3b globally in astrocytes modified gene expression in astrocytes and neurons. This is an interesting paper, providing new information related to gene and protein expression in amygdala astrocytes and how these change in the context of stress. The authors also provide novel and intriguing information related to astrocyte cilia. Finally, this work causally links amygdala astrocyte S1pr1 to the stress response, as noted in the title. The methodological and statistical approaches used are appropriate, and the quality of the data are high. The major concern noted to this manuscript, in its current iteration, is that the results presented do not support the conclusion that astrocyte cilia are causally linked to the stress response. This and several additional concerns are noted below."

We thank the reviewer for pointing out the novelty, interest and quality of our results. We addressed the issue related to the causal link between cilium and stress as well as all the comments below with new experiments, analyses and revisions. The reviewer's insightful points have made the manuscript stronger and clearer, and we are very grateful for the opportunity to improve our manuscript. Thank you.

Major concerns

1. *"The authors provide limited background information on cilia and their known functions in astrocytes. Considering the paper is centered around astrocyte primary cilia, additional details regarding this should be included in the Main section of the text and discussion."*

We thank the reviewer for this point. In fact, there are only a couple of papers on astrocyte primary cilia and we now mention them on page 2 and reference the relevant literature throughout, as requested. We also cite key reviews on cilium biology, recognizing that a summary of the reviews is not possible within the current manuscript's word limits. Furthermore, the request for greater discussion was requested by reviewer 2 and we have added and expanded section to the discussion on pages 10-11.

"Although a precise threshold for functionally meaningful changes in primary cilium length has not been established, the primary cilium is recognized as a morphologically dynamic organelle in which even modest alterations in length, either increases or decreases, can influence signaling and cellular function[4-9]. In addition to altering the amount, spatial distribution, or effective density of signaling components within the cilium, changes in ciliary length may influence cellular signaling through several other mechanisms[8,10]. Shortening of the primary cilium may reduce the available membrane surface for receptor localization, potentially affecting signal sensitivity to extracellular ligands. It may also influence intraflagellar transport dynamics, thereby altering the trafficking, delivery, or turnover of signaling proteins within the ciliary compartment. Changes in length could additionally modify the spatial organization of sub-ciliary domains (e.g., base, shaft, and tip)[8,10], potentially affecting interactions among receptors and scaffolding proteins. Because the primary cilium can function as a mechanosensor, shorter cilia may also alter the mechanical properties of the organelle and its ability to transduce physical

stimuli. Finally, cilium length may influence cellular states linked to ciliary dynamics, such as quiescence, all of which could contribute to altered downstream signaling outcomes. These possibilities may contribute to the changes reported in this study, but we are aware of no cases where these mechanisms have been teased apart and are not aware of genetic tools to do so systematically – reflecting the fact that exploring primary cilium biology *in vivo* is a nascent area of biology. Under normal homeostatic conditions, critical functions would be served by primary cilia around 4-5 μm in length, but such functions would change with shorter primary cilia. Consistent with a role for primary cilia in neurobiological responses, genetic ciliopathies frequently include neurological manifestations[10], supporting the view that ciliary perturbations can affect brain function. Together, our findings indicate that stress induces coordinated changes in cilium-associated genes, proteins and morphology in amygdala astrocytes, consistent with a role for astrocytic ciliary alterations in stress-related pathology. Further mechanistic studies are now needed to unlock the signaling and biophysics of the primary cilium."

2. *"Similarly, there is no discussion of S1pr1 gene or protein expression in the context of stress, or its ligand S1p- does it change, or is it known to change in the context of stress? The vast majority of S1p comes from blood, it seems this data should exist."*

Thank you for these comments. We have fully revised the relevant results section to report our data on S1pr1 more clearly and in relation to the stress-related contexts we study we do now report what happens to S1pr1 at the gene and protein level. Thus, on pages 7-8 we write:

"We confirmed that S1pr1 was highly and specifically expressed in astrocytes using RNAseq and near-plasma membrane proteomics from US mice (**Fig. 4c**) and found its expression was not altered by stress at the RNA or protein level (**Fig. 4c**)."

Later on page 10 we write,

"Consistent with the mouse data (**Fig. 4c**), *S1PR1* was not a DEG in the neuropsychiatric disorders reported in **Fig. 5n-u**."

We found no articles about the amygdala and S1P/S1PR1 levels in any neuropsychiatric disease. We only found one paper showing increased S1P levels after treatment of stress-related disorders[38]. We cite this paper on page 11 with the following sentences but are reluctant to draw any greater comparisons with our work because of the request by reviewer 1 to tone down such assertions.

"S1pr1 expression was robustly detected in amygdala astrocytes in both mouse and human tissue, emphasizing the translational potential of these findings related to S1pr1 drugs approved for use in humans[39]. The high level of S1pr1 in the plasma membrane proteome of amygdala astrocytes suggests that it exerts its actions from that location, rather than from the primary cilium. A past study has reported that blood S1P1 levels increase when stress-related behaviors were treated[38], but the relevance of those clinical data to our work will require further study. We also detected S1pr1 mRNA in regions corresponding to endothelial cells and future work should explore the function of this GPCR in those cells."

3. *"The authors demonstrate 1) cilia are different in stress 2) astrocytes have altered gene expression, including cilia related genes 3) targeting S1pr1 improved stress response and cilia 4) S1pr1 is also*

expressed in human amygdala astrocytes 5) cilia related genes are also abundant in human astrocytes 4) disrupting astrocyte cilia leads to changes in gene expression and changes in neurons. However, they did not show amygdala astrocytes contribute causally to stress-related behaviors through signaling related to their primary cilia – to do so they would have needed to show that disruption of astrocyte cilia cause stress related behavior. The did causally link astrocyte amygdala S1pr1 to the stress response. Further, the link between S1pr1 and cilia is not clearly articulated in the manuscript except to say that cilia signal with Gpcrs."

Thank you. In relation to causation, this was an important point also raised by reviewer 3. We addressed it on page 9 with the following section reporting new experiments. By disrupting Ift88 genetically in amygdala astrocytes, we triggered some stress related behaviors and molecular changes within astrocytes.

"Next, we used our AAV strategy to disrupt astrocyte primary cilia only in the amygdala in adult mice (**Fig. 2m**) and again performed amygdala bulk RNA seq (**Extended data Fig. 18**) and astrocyte-specific RNAseq with MACS[36] (**Fig. 5h-l**; **Extended data Fig. 17b-j**). Arl13b and Ift88 deletion from amygdala astrocytes resulted in 142 and 129 DEGs, respectively (**Fig. 5h-l**). In both cases, the key pathways were glial reactivity, extracellular matrix (ECM), cilium biology and collagen containing ECM (**Fig. 5j, l**). We validated collagen with IHC and found that it was decreased, and we validated perineuronal nets known to be replete with ECM components with WFA and found a significant increase (**Extended data Fig. 17k-n**) – both these changes were in accord with RNAseq (**Fig. 5d-l**). Furthermore, astrocyte reactivity genes were mostly down regulated (**Extended data Fig. 17j**), which is consistent with the lower levels of GFAP following stress (**Fig. 1k, l**). Next, we performed behavior on mice in which amygdala astrocyte Ift88 was deleted and was assessed with three markers (**Fig. 2o**) and RNAseq (**Fig. 5k**). We found significant deficits in stress-related behaviors measured by OFT, but not in those measured by SPT and FST (**Fig. 5m**; 16-17 mice per group). Overall, these data provide strong evidence that loss of primary cilia within amygdala astrocytes alone can alter astrocytes, surrounding cells, and lead to some stress-related behaviors. These data imply that amygdala astrocyte primary cilia signaling contributes markedly to anxiety states, in accord with recent work[37], whereas other stress-related behaviors (**Fig. 1**) require convergence of additional changes evoked by CRS and WAS."

In relation to the second point for clearer articulation, we have revised the manuscript to better explain the conceptual transition from astrocyte cilia to GPCR signaling and to make the rationale more explicit in the relevant section (page 6). Specifically, we added the following text:

"GPCRs are known to regulate multiple aspects of astrocyte morphology[25] and signaling[26]. Because we observed reduced astrocyte primary cilium lengths, we next considered signaling pathways that are strongly associated with this organelle. Primary cilia are well-established hubs for GPCR-mediated signaling[27], and GPCR pathways also play key roles in astrocyte function, including within the amygdala[28]. Based on this overlap, we analyzed our proteomic datasets (**Fig. 2d-f**) from US, CRS, and WAS groups to identify GPCRs expressed in amygdala astrocytes and to determine their predicted G-protein coupling[29,30] (**Fig. 3a-c**). Based on these data we hypothesized that activation of GPCR signaling pathways might influence primary cilium morphology. Among the GPCRs detected in astrocytes by proteomics, most were predicted to couple to Gi and/or Gq proteins[31-34] (**Fig. 3a-c**; e.g., Gi-coupled receptors S1pr1, Fzd2, Grm2, Grm3; Gq-coupled receptors Grm5, Grm1, Sstr2, Chrm3), which led us to test whether astrocyte-specific activation of GPCR signaling[28-30,35] pathways could influence stress-evoked behaviors."

4. *"Related to the point above, it is not clear if the authors are claiming S1pr1 is expressed in cilia, or the alteration of S1pr1 generally in astrocytes is sufficient to rescue alterations observed in cilia."*

Thank you for this comment. We addressed this on pages 7-8 with new data and textual changes. On page 7-8 we write:

"We confirmed that S1pr1 was highly and specifically expressed in astrocytes using RNAseq and near-plasma membrane proteomics from US mice (**Fig. 4c**) and found its expression was not altered by stress at the RNA or protein level (**Fig. 4c**). In accord S1pr1 was detected in amygdala astrocytes from a mouse snRNAseq database[11] (**Fig. 4d; Extended data Fig. 13a, b**). Using RNAscope we found that all S100β-positive amygdala astrocytes expressed S1pr1 transcripts (**Fig. 4e**; n = 3 mice). Expression was dense in astrocyte somata but was also found within end feet and in end foot-adjacent regions that are likely endothelial cells[40] (**Extended data Fig. 14a**). S1pr1 was not found within astrocyte primary cilia (**Extended data Fig. 14b**). In accord, with the mouse data, *S1PR1* was expressed in human astrocytes from snRNAseq databases[24] (**Fig. 4f**; **Extended data Fig. 13c**) and by performing RNAscope experiments we found that ~50% of *ALDH1L1*-positive amygdala astrocytes from neurotypical human subjects expressed *S1PR1* (**Fig. 4g, h**; n = 4 human brain samples)."

Please also note that Fig 6 and text on page 10 now report the astrocyte primary cilium proteome (for the first time) and whereas we identified multiple known cilial proteins, we did not find S1pr1. This analysis also supports the paragraph above.

5. "There is gap in logic between the following aspects of the manuscript: 1) Therefore, targeting native, highly expressed astrocyte S1pr1 Gi-GPCRs produced similar restorative effects to those observed with astrocyte Gi-GPCR chemogenetics and the following paragraph 2) Since astrocytes regulate neurons and other cells locally within their territories, we deleted the primary cilium gene Arl13b in amygdala astrocytes (based on methods provided, this was actually all astrocytes) and performed bulk RNAseq to shed light on how gene expression reflecting all amygdala cells was altered."

Thank you for this. We have revised large parts of the manuscript to make the logic of the various sections clearer. The first part of this comment seems to be a repeat of preceding ones (comments 3 and 4) and address S1pr1 and GPCRs. We kindly refer the reviewer to those comments and our detailed responses.

In terms of deleting Arl13b from all astrocytes, the reviewer is correct and those data are included in Fig 5d-g. However, to address the reviewer's comment directly we include three new experiments by deleting primary cilia genes only with amygdala astrocytes. These data are reported in Fig 5h-m (ED Fig 18). We believe the inclusion of these new data on page 9 with textual clarifications of the logic improves the presentation and addresses the key points with new data. These new data directly show that deleting primary cilia genes within amygdala astrocytes can alter their molecular properties and lead to some stress-related behaviors. This is an exciting finding, and we thank the reviewer for prompting us to do this work.

6. *"Is S1pr1 gene or protein expression altered in the context of stress in mice or is it changed in human data? Does the ligand change in the context of stress?"*

Thank you. This seems to be a repeat of comment 2 above. We kindly refer the reviewer to that response.

7. *Can the authors speculate regarding the following statement? 'We found that amygdala astrocytes expressed more cilium-related genes than most other regions evaluated . i.e. what does this mean, are their cilia longer or more numerous?*

Thank you. This is an excellent comment. As requested, we provide the necessary speculation on pages 8-9 with the following sentences.

"However, consistent with our findings related to the primary cilium (**Fig. 1-4**), we found that amygdala astrocyte expression of cilium-related genes[23] was higher than most other regions (**Fig. 5c; Extended data Fig. 17a)**, which suggests that astrocyte primary cilium biology may be region specific and relevant for the amygdala. This is consistent with the emerging field of astrocyte diversity[41] and the functions of astrocyte subtypes[15,17,42-44]. In these regards, the molecular profiles we report enable mechanistic study of amygdala astrocyte responses *in vivo*[28,37]."

8. *Are the cilia changes restricted to amygdala, or astrocytes? At a minimum this should be discussed, but to support the manuscript, should be quantified.*

Thank you. This comment was shared with Reviewer 1. We now report new data to address this directly. To address whether stress-induced changes in primary cilia are specific to astrocytes or also occur in other cell types, we performed additional analyses examining cilia length in both astrocytes and neurons. In the amygdala, the reduction in primary cilium length following stress was observed in astrocytes but not in neurons (Fig. 2q, t). We also conducted similar analyses in the cortex and found that primary cilium length was not altered in either astrocytes or neurons in this region (ED Fig 5 and 6). These new data directly address the reviewer's question and are now included on page 5.

9. *"The authors provide evidence that anxiety behaviors in mice can be attenuated through the administration of antidepressants. These experiments distract from the primary goals of the paper, which is determining the effects of stress on amygdala astrocytes. Additional context should be provided as to why these experiments were included. Additionally, it would be useful to see whether cilia are affected by these drugs, considering later in the manuscript poneismod is utilized and similarly attenuates anxiety behaviors."*

We appreciate this point. Since reviewer 1 asked to us comment more about the face validity of the stress experiments, for which the response to drugs is a key part, we have opted to report the data in the main manuscript with the figures now only in the Extended data. Accordingly, the text has been very much shortened, and we think this addresses the comment from the reviewer about the previous version being distracting in these regards. On pages 3-4 we now write

"Since stress-related behaviors such as depression are often treated with antidepressants[45], we determined which of the metrics reported in the previous section were altered by two drugs used to treat depression[46,47] (30 mg/kg ketamine and 10 mg/kg fluoxetine; n = 10 mice per group, respectively, and n = 9-10 vehicle controls) for CRS because it produced the most reliable behavioral changes (**Fig. 1**). While both fluoxetine and ketamine improved some CRS-evoked behaviors, ketamine was more effective (**Extended data Fig. 1c-g**). Antidepressants are incompletely effective[48], and so complete rescue of the stress-related behavioral changes was not expected and did not occur (**Extended data Fig. 1c-g**)."

As requested by the reviewer, we also performed experiments to see if the reduction in primary cilium length caused by stress was affected by ketamine and report these data on page 5 with the following sentences

"We also performed a specific set of experiments to assess the effects of ketamine, which reduced some of the stress-related behavioral changes (**Extended data Fig. 1f, g**). We found that ketamine's effects were accompanied by significant restoration of amygdala astrocyte primary cilium length: no effects were observed for neuronal primary cilia (**Extended data Fig. 7**). We do not interpret this result to suggest that ketamine works via the astrocyte primary cilium, but instead that the astrocyte primary cilium is recovered during the beneficial response to ketamine, which is known to have a complex pharmacological mode of action[49]."

10. *"The authors state that S1pr1 is highly specific to astrocytes. However, figure 5d clearly shows high levels of S1pr1 expression in endothelial cells. Additionally, extended data fig 12b shows very little S1pr1 expression in humans. The authors should comment on the potential effects ponesimod may have on endothelial cells, the discrepancies in S1pr1 expression, and how this fits into the context of their study."*

Thank you. We are happy to address this and think there was a slight misunderstanding also. You see, former ED Fig. 12b (now ED Fig. 13c) shows all human basal and lateral amygdala cells, where most *S1pr1* expression was found in the cluster that corresponds to astrocytes (yellow). The other cells express very little. To be sure human astrocytes express S1PR1, we nonetheless performed RNAscope experiments in ALDH1L1 positive astrocytes, and these data are reported in Fig 4g,h. We have made these points clearer in the revised manuscript.

However, we also agree with the reviewer that in our mouse RNAscope experiments (Fig 4e, ED Fig 14a) we do see S1pr1 in areas corresponding to blood vessels. We accurately report this on page 8 with the following sentences.

"Using RNAscope we found that all S100β-positive amygdala astrocytes expressed S1pr1 transcripts (**Fig. 4e**; n = 3 mice). Expression was dense in astrocyte somata but was also found within end feet and in end foot-adjacent regions that are likely endothelial cells[40] (**Extended data Fig. 14a**)."

Later in the discussion we write

"We also detected S1pr1 mRNA in regions corresponding to endothelial cells and future work should explore the function of this GPCR in those cells."

11. *"Please rephrase sentence 1 on page 8 to reflect that Arll3b was deleted in all Aldh1l1+ astrocytes, as well as the sentence in the discussion, 'Genetic disruption of primary cilia by deletion of a key gene (Arl13b) within amygdala astrocytes resulted in non-cell-autonomous effects, perturbing the molecular properties of neighboring parenchymal cells, including neurons."*

Thank you. We have made those changes (now on page 9) but also note that we have now included new data where Arl13b (and Ift88) were deleted from only amygdala astrocytes, which addresses the broader point of astrocyte and amygdala specific perturbation of primary cilia. As requested, the discussion has been changed to address the reviewer's comments and to summarize the new data.

12. *"The following statement in not accurate' In this study, we identified a causal contributory role for amygdala astrocytes in the modulation of stress-related behaviors, mediated via signaling pathways associated with their primary cilia'. This study demonstrated manipulation of amygdala astrocyte S1pr1 was causally linked the modulation of stress-related behaviors. No direct link to cilia was provided."*

Thank you. We have toned down our assertions in these regards throughout the manuscript and are grateful for this wise guidance. Kindly note that we have now included data where Arl13b (and Ift88) was deleted from only amygdala astrocytes, and that we report molecular changes, changes in primary cilia and in some stress-related behaviors (Fig 5h-m; page 9). These new data, along with the more cautious interpretation suggested, address the comment by the reviewer.

<u>Minor concerns</u>
1. *"Stress evoked molecular changes…" paragraph should specify the isolation of amygdala astrocyte-specfic RNA-seq"*

We thank the reviewer for pointing this out and have updated the text with the suggested changes on page 4.

2. *"The authors utilize DREADDS to target GPCR signaling, which are known to affect baseline characteristics of expressing cells, but do not evaluate baseline differences here. As the authors follow up on and confirm their findings with pharmacological approaches, these experiments are not necessary, but should be acknowledged nonetheless. In general, the authors do not indicate limitations of their study."*

Thank you. We did in fact evaluate if hM4Di affected baseline behaviors of relevance to this study in unstressed mice. These data are reported in ED Fig 10b. No effects were observed, which is why we adopted the overall experimental plan. We have now made this clearer on page 6 near the bottom.

In addition, the limitations of the study are mentioned throughout, and we have toned some aspects down as requested.

3. *"The authors show that activation of GPCR signaling via deschloroclozapine in DREADD expressing astrocytes in response to stress elevates Ca2+ microdomain firing. Does this return Ca2+ activation to levels seen in unstressed mice?"*

Yes. We had included a control group in the hM4Di calcium signaling experiment (ED Fig. 12) that was omitted in the previous version for the sake of simplicity. The data show that the frequency of calcium signaling in hM4Di stressed mice returns to levels seen in hM4Di unstressed mice in both somata and microdomains.

4. *"Under "Molecular properties of amygdala astrocytes" authors should specify Rpl22HA Ribotag Mice were crossed with Aldh1l1 specific mice to generate astrocyte specificity."*

Thank you. We did this on page 8 as suggested.

5. *"Are there any known off targets of ponesimod? Regardless, this should be acknowledged in the text."*

Based on the FDA Drug Approval Package for PONVORY (ponesimod), based on the known off-target binding activity, Ponesimod showed greater than 50% inhibition of binding to three targets: Endothelin ETA receptor ~ 74% inhibition; Monoamine oxidase MAO-B ~62% inhibition and Protein serine/threonine kinase Ca^{2+}/calmodulin-dependent II (CaMKII) ~63% inhibition. However, several factors were suggested to show that these off-target effects are negligible, - principally the approximately 1,750-fold difference between the S1PR1 Ki (5.7 nM) and the screening concentration (10 μM) suggests that at clinically relevant doses in humans, ponesimod's effects will be dominated by S1PR1 activation. We have briefly mentioned that off-target effects will need to be considered and feel the right place to do that was in the methods on page 25 – especially since reviewer 3 asked us to tone down the translational aspects of the work in the main text.

6. *"Another exciting finding from the authors is that in response to cilia elimination via Arl13b deletion, many key DEGs altered are related to perineuronal nets and perisynaptic extracellular matrix. Have the authors confirmed these changes via IHC through WFA? Providing this not only strengthens the conclusions here, but would provide additional foundations for future studies."*

We thank the reviewer for this suggestion that strengthens the findings of our study. Yes, we used IHC to confirm some of the RNAseq changes. We found decreased collagen IV levels and increased WFA staining, suggesting an altered perisynaptic extracellular matrix in response to cilium deletion in BLA astrocytes. These results are shown in ED Fig. 17k-n and were predicted by the RNAseq data - as the reviewer correctly stated.

7. *"Under "Mouse Models", the authors should provide the IACUC protocol numbers relevant to their study"*

We thank the reviewer for pointing this out. The information is provided on page 22.

8. *"Did the authors assess any sex differences?"*

In the present study, we used approximately equal numbers of male and female mice across all stress paradigms and behavioral/molecular analyses. We found no sex differences in stress-induced physiological and behavioral changes, astrocyte ciliary morphology, molecular dysregulation, or responses to DREADD or S1PR1 agonist manipulations. A statement to this effect is made on page 22.

9. *"Under "Sucrose preference test (SPT)", authors should include whether animals were returned to group housing following social isolation, as long-term social isolation can affect glial reactivity in the brain."*

We thank the reviewer for raising this important point regarding housing conditions and potential confounding effects of social isolation on glial responses. In our study, animals were not returned to group housing following the SPT because after SPT the animals were immediately euthanized for tissue collection. This applied to all groups.

10. *"Under "Von Frey test", the word (Ref) is present, and is likely a typo."*

Thank you for pointing out this typo. We have corrected the text.

11. *"Under "Immunohistochemistry (IHC)", third paragraph, the authors should include key metrics for their analysis, including smoothing factor, type of thresholding used (absolute or local intensity), and how the threshold value was calculated for both thresholding and segmentation. This would aid in reproducibility of the analysis."*

We have included the key metrics in the methods section as suggested on page 27.

12. *"Under "analysis of biotinylated proteins using mass spectrometry", the authors can remove the sentence of where the data is deposited, as it is included in the data availability section."*

Thank you. We have updated the text with the suggested changes.

13. *"Did the authors perform any power analysis prior to experimentation to determine sample size, if so, how was this completed and if not, why this was not done."*

Thank you for raising this point. No formal *a priori* power analysis was performed before initiating these experiments. This is largely because there was no prior work specifically examining the contribution of amygdala astrocytes to stress-related behaviors assessed, and therefore no reliable estimates of expected effect sizes or variance were available to inform such calculations. Instead, sample sizes were determined based on group sizes commonly used in comparable behavioral neuroscience studies, as well as in our laboratory's prior experience with behavioral paradigms and other astrocyte manipulation experiments. These sample sizes are consistent with those typically used in the field and are reported in the data reporting summary submitted with the manuscript. Importantly, the key findings were replicated across independent experimental cohorts and across complementary experimental approaches, which supports the robustness of the observed effects. In addition, all replicate-level measurements are provided in the source data files to ensure full transparency. By reporting the complete datasets, our study now provides empirically derived parameters that will enable accurate power calculations for future work examining astrocyte contributions to stress-related behaviors.

14. *"The authors state no data points were excluded from any experiment. Did the authors have any exclusion criteria, such as adverse reactions following surgery, to drugs, etc.?"*

Yes, we did have pre-established exclusion criteria that we applied consistently across all experiments and groups. Regarding the data points, what we meant to write was that no data points were excluded after the exclusion criteria. Pre-established exclusion criteria included:

Surgical complications: Evidence of infection at the injection site, excessive bleeding, or failure to recover from anesthesia within expected time frames. Incorrect cannula placement or incomplete viral expression.

Clinical signs requiring immediate euthanasia: If symptom 1 or symptom 2 is observed during daily general health observations, the animal will be excluded and euthanized promptly. Symptom 1: Decreased body temperature (reduced skin warmth), persistent sedation (e.g., huddling), or significantly reduced resistance to handling. Symptom 2: Persistent seizures and unresponsiveness with poor prognosis for recovery.

Drug-related adverse reactions: Unexpected severe reactions to ketamine, fluoxetine, ponesimod, or DCZ (e.g., respiratory depression, severe tremors, convulsions) that met the clinical criteria outlined above or required veterinary intervention.

We think these pre-established conditions are standard as they essentially state that mice that became sick were not used. Our mouse protocols also require that mice that become sick are not used in experiments. Over the course of 4 years and ~300 mice, the application of these exclusion criteria meant that 5 mice were removed. This information is now provided in the data reporting summary, and we thank the reviewer for the comment to add this.

15. *"Fig 2i would benefit from visualizing the raw signal to see the effectiveness of segmentation."*

We included the raw signal in the previous version of the manuscript but were remiss in not explicitly calling it out in the text. We have now included the call out in the text and the raw signal can be found in ED Fig. 4h. In addition, we have now included more examples of raw cilium staining throughout the manuscript in the main figures and in the extended data (for example, in Fig. 2h, 2n; ED Fig. 5c-d).

Referee #5 (Remarks to the Author):

"I co-reviewed this manuscript with one of the reviewers who provided the listed reports."

Thank you. Your points are all addressed in responses to Referee 4.

Thank you to all five reviewers. Your comments have made our manuscript much better. Thank you.

References cited here

Note that the citation numbers in this file are not the same as in the manuscript, but in each case the citation number is correct and corresponds to the correct paper. In other words, the response to reviewers and manuscript files were processed separately in Endnote.

1 Daviu, N., Bruchas, M. R., Moghaddam, B., Sandi, C. & Beyeler, A. Neurobiological links between stress and anxiety. *Neurobiology of stress* **11**, 100191, doi:10.1016/j.ynstr.2019.100191 (2019).

2 Zhang, W. H., Zhang, J. Y., Holmes, A. & Pan, B. X. Amygdala Circuit Substrates for Stress Adaptation and Adversity. *Biological psychiatry* **89**, 847-856, doi:10.1016/j.biopsych.2020.12.026 (2021).

3 Maren, S. & Holmes, A. Stress and Fear Extinction. *Neuropsychopharmacology* **41**, 58-79, doi:10.1038/npp.2015.180 (2016).

4 Yang, J., Dong, Y., Liu, J., Peng, Y., Wang, D., Li, L., Hu, X., Li, J., Wang, L., Chu, J., Ma, J., Shi, H. & Shi, S. H. Primary ciliary protein kinase A activity in the prefrontal cortex modulates stress in mice. *Neuron* **113**, 1276-1289.e1275, doi:10.1016/j.neuron.2025.02.002 (2025).

5 Macarelli, V., Leventea, E. & Merkle, F. T. Regulation of the length of neuronal primary cilia and its potential effects on signalling. *Trends in cell biology* **33**, 979-990, doi:10.1016/j.tcb.2023.05.005 (2023).

6 Miyoshi, K., Kasahara, K., Murakami, S., Takeshima, M., Kumamoto, N., Sato, A., Miyazaki, I., Matsuzaki, S., Sasaoka, T., Katayama, T. & Asanuma, M. Lack of dopaminergic inputs elongates

the primary cilia of striatal neurons. *PLoS One* **9**, e97918, doi:10.1371/journal.pone.0097918 (2014).
7 Schou, K. B., Pedersen, L. B. & Christensen, S. T. Ins and outs of GPCR signaling in primary cilia. *EMBO reports* **16**, 1099-1113, doi:10.15252/embr.201540530 (2015).
8 Hilgendorf, K. I., Myers, B. R. & Reiter, J. F. Emerging mechanistic understanding of cilia function in cellular signalling. *Nature reviews. Molecular cell biology* **25**, 555-573, doi:10.1038/s41580-023-00698-5 (2024).
9 Derderian, C., Canales, G. I. & Reiter, J. F. Seriously cilia: A tiny organelle illuminates evolution, disease, and intercellular communication. *Dev Cell* **58**, 1333-1349, doi:10.1016/j.devcel.2023.06.013 (2023).
10 Mill, P., Christensen, S. T. & Pedersen, L. B. Primary cilia as dynamic and diverse signalling hubs in development and disease. *Nature reviews. Genetics* **24**, 421-441, doi:10.1038/s41576-023-00587-9 (2023).
11 Sun, W., Liu, Z., Jiang, X., Chen, M. B., Dong, H., Liu, J., Südhof, T. C. & Quake, S. R. Spatial transcriptomics reveal neuron-astrocyte synergy in long-term memory. *Nature* **627**, 374-381, doi:10.1038/s41586-023-07011-6 (2024).
12 Sanz, E., Yang, L., Su, T., Morris, D. R., McKnight, G. S. & Amieux, P. S. Cell-type-specific isolation of ribosome-associated mRNA from complex tissues. *Proc Natl Acad Sci U S A* **106**, 13939-13944, doi:10.1073/pnas.0907143106. PMCID: PMC2728999 (2009).
13 Bravo-Ferrer, I., Khakh, B. S. & Díaz-Castro, B. Cell-specific RNA purification to study translatomes of mouse central nervous system. *STAR protocols* **3**, 101397, doi:10.1016/j.xpro.2022.101397 (2022).
14 O'Dea, M. R. & Hasel, P. Are we there yet? Exploring astrocyte heterogeneity one cell at a time. *Glia* **73**, 619-631, doi:10.1002/glia.24621 (2025).
15 Chai, H., Diaz-Castro, B., Shigetomi, E., Monte, E., Octeau, J. C., Yu, X., Cohn, W., Rajendran, P. S., Vondriska, T. M., Whitelegge, J. P., Coppola, G. & Khakh, B. S. Neural circuit-specialized astrocytes: transcriptomic, proteomic, morphological, and functional evidence. *Neuron* **95**, 531-549. PMCID: PMC5811312 (2017).
16 Srinivasan, R., Lu, T. Y., Chai, H., Xu, J., Huang, B. S., Golshani, P., Coppola, G. & Khakh, B. S. New Transgenic Mouse Lines for Selectively Targeting Astrocytes and Studying Calcium Signals in Astrocyte Processes In Situ and In Vivo. *Neuron* **92**, 1181-1195, doi:10.1016/j.neuron.2016.11.030. PMCID: PMC5403514 (2016).
17 Endo, F., Kasai, A., Soto, J. S., Yu, X., Qu, Z., Hashimoto, H., Gradinaru, V., Kawaguchi, R. & Khakh, B. S. Molecular basis of astrocyte diversity and morphology across the CNS in health and disease. *Science* **378**, eadc9020, doi:10.1126/science.adc9020. PMCID Pending (2022).
18 Higginbotham, H., Eom, T. Y., Mariani, L. E., Bachleda, A., Hirt, J., Gukassyan, V., Cusack, C. L., Lai, C., Caspary, T. & Anton, E. S. Arl13b in primary cilia regulates the migration and placement of interneurons in the developing cerebral cortex. *Dev Cell* **23**, 925-938, doi:10.1016/j.devcel.2012.09.019 (2012).
19 Higginbotham, H., Guo, J., Yokota, Y., Umberger, N. L., Su, C. Y., Li, J., Verma, N., Hirt, J., Ghukasyan, V., Caspary, T. & Anton, E. S. Arl13b-regulated cilia activities are essential for polarized radial glial scaffold formation. *Nat Neurosci* **16**, 1000-1007, doi:10.1038/nn.3451 (2013).
20 Kasahara, K., Miyoshi, K., Murakami, S., Miyazaki, I. & Asanuma, M. Visualization of astrocytic primary cilia in the mouse brain by immunofluorescent analysis using the cilia marker Arl13b. *Acta medica Okayama* **68**, 317-322, doi:10.18926/amo/53020 (2014).
21 Sterpka, A. & Chen, X. Neuronal and astrocytic primary cilia in the mature brain. *Pharmacological research* **137**, 114-121, doi:10.1016/j.phrs.2018.10.002 (2018).
22 Lahaie, S. C., Fok, A. H. K., Nicholls, J. M., Lee, H., Syed, T. A., Chierzi, S., Hatada, S., Egawa, N., Schober, A. L., Wong, T. Y. M., Royston, R., Siddiqi, K., Kubota, Y. & Murai, K. K. Characterization of astrocytic primary cilia in the adult mouse cortex and hippocampus. *Heliyon* **11**, e44010, doi:https://doi.org/10.1016/j.heliyon.2025.e44010 (2025).

23 Wang, L., Guo, Q., Acharya, S., Zheng, X., Huynh, V., Whitmore, B., Yimit, A., Malhotra, M., Chatterji, S., Rosin, N., Labit, E., Chipak, C., Gorzo, K., Haidey, J., Elliott, D. A., Ram, T., Zhang, Q., Kuipers, H., Gordon, G., Biernaskie, J. & Guo, J. Primary cilia signaling in astrocytes mediates development and regional-specific functional specification. *Nat Neurosci* **27**, 1708-1720, doi:10.1038/s41593-024-01726-z (2024).

24 Siletti, K., Hodge, R., Mossi Albiach, A., Lee, K. W., Ding, S. L., Hu, L., Lönnerberg, P., Bakken, T., Casper, T., Clark, M., Dee, N., Gloe, J., Hirschstein, D., Shapovalova, N. V., Keene, C. D., Nyhus, J., Tung, H., Yanny, A. M., Arenas, E., Lein, E. S. & Linnarsson, S. Transcriptomic diversity of cell types across the adult human brain. *Science* **382**, eadd7046, doi:10.1126/science.add7046 (2023).

25 Baldwin, K. T., Murai, K. K. & Khakh, B. S. Astrocyte morphology. *Trends in cell biology* **34**, 547-565, doi:10.1016/j.tcb.2023.09.006 (2024).

26 Khakh, B. S. & McCarthy, K. D. Astrocyte calcium signaling: from observations to functions and the challenges therein. *Cold Spring Harb Perspect Biol* **Jan 20;7(4):a020404. doi: 10.1101/cshperspect.a020404.** (2015).

27 Wang, C. E. & Ogden, S. K. G Protein-Coupled Receptor Signal Intersection at the Primary Cilium. *BioEssays : news and reviews in molecular, cellular and developmental biology*, e70015, doi:10.1002/bies.70015 (2025).

28 Bukalo, O., O'Sullivan, R., Tanisumi, Y., Mendez, A., Weinholtz, C., Zimmerman, S., Offenberg, V., Carpenter, O., Bhagwat, H., Mosley, S., O'Malley, J. J., Lyons, K., Fang, Y., Goldschlager, J., Ostroff, L. E., Penzo, M. A., Wake, H., Halladay, L. R. & Holmes, A. Astrocytes enable amygdala neural representations supporting memory. *Nature*, doi:10.1038/s41586-025-10068-0 (2026).

29 Soto, J. S., Neupane, C., Kaur, M., Pandey, V., Wohlschlegel, J. A. & Khakh, B. S. Astrocyte Gi-GPCR signaling corrects compulsive-like grooming and anxiety-related behaviors in Sapap3 knockout mice. *Neuron* **112**, 3412-3423.e3416, doi:10.1016/j.neuron.2024.07.019 (2024).

30 Yu, X., Nagai, J., Marti-Solano, M., Soto, J. S., Coppola, G., Babu, M. M. & Khakh, B. S. Context-Specific Striatal Astrocyte Molecular Responses Are Phenotypically Exploitable. *Neuron* **108**, 1146-1162.e1110, doi:10.1016/j.neuron.2020.09.021. PMCID: PMC7813554 (2020).

31 Flock, T., Hauser, A. S., Lund, N., Gloriam, D. E., Balaji, S. & Babu, M. M. Selectivity determinants of GPCR-G-protein binding. *Nature* **545**, 317-322, doi:10.1038/nature22070 (2017).

32 Harding, S. D., Armstrong, J. F., Faccenda, E., Southan, C., Alexander, S. P. H., Davenport, A. P., Spedding, M. & Davies, J. A. The IUPHAR/BPS Guide to PHARMACOLOGY in 2024. *Nucleic acids research* **52**, D1438-d1449, doi:10.1093/nar/gkad944 (2024).

33 Inoue, A., Raimondi, F., Kadji, F. M. N., Singh, G., Kishi, T., Uwamizu, A., Ono, Y., Shinjo, Y., Ishida, S., Arang, N., Kawakami, K., Gutkind, J. S., Aoki, J. & Russell, R. B. Illuminating G-Protein-Coupling Selectivity of GPCRs. *Cell* **177**, 1933-1947.e1925, doi:10.1016/j.cell.2019.04.044 (2019).

34 Pándy-Szekeres, G., Munk, C., Tsonkov, T. M., Mordalski, S., Harpsøe, K., Hauser, A. S., Bojarski, A. J. & Gloriam, D. E. GPCRdb in 2018: adding GPCR structure models and ligands. *Nucleic acids research* **46**, D440-d446, doi:10.1093/nar/gkx1109 (2018).

35 Shah, D., Gsell, W., Wahis, J., Luckett, E. S., Jamoulle, T., Vermaercke, B., Preman, P., Moechars, D., Hendrickx, V., Jaspers, T., Craessaerts, K., Horré, K., Wolfs, L., Fiers, M., Holt, M., Thal, D. R., Callaerts-Vegh, Z., D'Hooge, R., Vandenberghe, R., Himmelreich, U., Bonin, V. & De Strooper, B. Astrocyte calcium dysfunction causes early network hyperactivity in Alzheimer's disease. *Cell Rep* **40**, 111280, doi:10.1016/j.celrep.2022.111280 (2022).

36 Holt, L. M., Stoyanof, S. T. & Olsen, M. L. Magnetic Cell Sorting for In Vivo and In Vitro Astrocyte, Neuron, and Microglia Analysis. *Current protocols in neuroscience* **88**, e71, doi:10.1002/cpns.71 (2019).

37 Ghenissa, O., Guayasamin, M., Ngo, K., Duquenne, M., Peyrard, S., Amilhon, B. & Murphy-Royal, C. Basolateral amygdala astrocytes encode anxiety states. *Neuron* **in press** (2026).

38 Werner, F., Schumacher, F., Mühle, C., Adler, W., Schug, C., Schäflein, E., Morawa, E., Kleuser, B., Kornhuber, J., Erim, Y. & Rhein, C. Psychosomatic - psychotherapeutic treatment of stress-

related disorders impacts the sphingolipid metabolism towards increased sphingosine and sphingosine-1-phosphate levels. *European archives of psychiatry and clinical neuroscience* **275**, 2049-2058, doi:10.1007/s00406-025-01985-2 (2025).
39 Pérez-Jeldres, T., Alvarez-Lobos, M. & Rivera-Nieves, J. Targeting Sphingosine-1-Phosphate Signaling in Immune-Mediated Diseases: Beyond Multiple Sclerosis. *Drugs* **81**, 985-1002, doi:10.1007/s40265-021-01528-8 (2021).
40 Weigel, C., Bellaci, J. & Spiegel, S. Sphingosine-1-phosphate and its receptors in vascular endothelial and lymphatic barrier function. *J Biol Chem* **299**, 104775, doi:10.1016/j.jbc.2023.104775 (2023).
41 Khakh, B. S. & Deneen, B. The emerging nature of astrocyte diversity. *Annu Rev Neurosci* **42**, 187-207 (2019).
42 Ollivier, M., Soto, J. S., Linker, K. E., Moye, S. L., Jami-Alahmadi, Y., Jones, A. E., Divakaruni, A. S., Kawaguchi, R., Wohlschlegel, J. A. & Khakh, B. S. Crym-positive striatal astrocytes gate perseverative behaviour. *Nature* **627**, 358-366, doi:10.1038/s41586-024-07138-0 (2024).
43 Hasel, P., Rose, I. V. L., Sadick, J. S., Kim, R. D. & Liddelow, S. A. Neuroinflammatory astrocyte subtypes in the mouse brain. *Nat Neurosci* **24**, 1475-1487, doi:10.1038/s41593-021-00905-6 (2021).
44 John Lin, C. C., Yu, K., Hatcher, A., Huang, T. W., Lee, H. K., Carlson, J., Weston, M. C., Chen, F., Zhang, Y., Zhu, W., Mohila, C. A., Ahmed, N., Patel, A. J., Arenkiel, B. R., Noebels, J. L., Creighton, C. J. & Deneen, B. Identification of diverse astrocyte populations and their malignant analogs. *Nat Neurosci* **20**, 396-405, doi:10.1038/nn.4493. PMCID: PMC5824716 (2017).
45 Cipriani, A., Furukawa, T. A., Salanti, G., Chaimani, A., Atkinson, L. Z., Ogawa, Y., Leucht, S., Ruhe, H. G., Turner, E. H., Higgins, J. P. T., Egger, M., Takeshima, N., Hayasaka, Y., Imai, H., Shinohara, K., Tajika, A., Ioannidis, J. P. A. & Geddes, J. R. Comparative efficacy and acceptability of 21 antidepressant drugs for the acute treatment of adults with major depressive disorder: a systematic review and network meta-analysis. *Lancet* **391**, 1357-1366, doi:10.1016/s0140-6736(17)32802-7 (2018).
46 Walker, A. K., Budac, D. P., Bisulco, S., Lee, A. W., Smith, R. A., Beenders, B., Kelley, K. W. & Dantzer, R. NMDA receptor blockade by ketamine abrogates lipopolysaccharide-induced depressive-like behavior in C57BL/6J mice. *Neuropsychopharmacology* **38**, 1609-1616, doi:10.1038/npp.2013.71 (2013).
47 Berman, R. M., Cappiello, A., Anand, A., Oren, D. A., Heninger, G. R., Charney, D. S. & Krystal, J. H. Antidepressant effects of ketamine in depressed patients. *Biological psychiatry* **47**, 351-354, doi:10.1016/s0006-3223(99)00230-9 (2000).
48 Aan Het Rot, M., Zarate, C. A., Jr., Charney, D. S. & Mathew, S. J. Ketamine for depression: where do we go from here? *Biological psychiatry* **72**, 537-547, doi:10.1016/j.biopsych.2012.05.003 (2012).
49 Li, L. & Vlisides, P. E. Ketamine: 50 Years of Modulating the Mind. *Frontiers in human neuroscience* **10**, 612, doi:10.3389/fnhum.2016.00612 (2016).

Dear [redacted]

Thank you for your email stating that our revised manuscript is accepted in principle. We have now addressed the pending editorial changes and the final points made by the referees. We have made all the requested changes in the revised manuscript and provide a point-by-point response below.

The changes we made are those that we discussed and agreed with you by email a few weeks ago. All changes in the manuscript are shown in blue text so you can spot them.

With these changes, we hope the manuscript will go to press.

All the best,

[redacted]

Baljit S. Khakh PhD
Professor (on behalf of all authors)
June 21st, 2026

Referee #1 (Remarks to the Author):

"*The authors have done an excellent job incorporating the comments into the manuscript and have significantly improved the manuscript with additional experiments that significantly adds value to the manuscript. We appreciate the rigour the authors have put into addressing our concerns and while the experiments and additional data have addressed some, not all of our questions have been addressed. For instance, the questions around the mechanistic molecular or circuit level link were not addressed experimentally. Two major points that the authors should address are:*"

We thank the reviewer for recognizing the additional experiments, the rigor of the study, and the substantial improvement of the manuscript. We also appreciate the remaining concerns, which we address below.

"The authors provided human sequencing data that shows disruption of key cilia genes in neuropsychiatric diseases to provide rationale of relevance. However, the authors report both gene expressions that both increased and decreased in disease and these do not appear to be the same in the genes that were changed in the conditionally knocked out mice or in the stress mouse models. The authors should include an analysis of the overlap between these genes that changed between humans and mice and if there is no overlap, explain/speculate why. Dysregulation of ciliated genes in neuropsychiatric diseases in human in either direction is not obviously causal, thus the authors need to link this back to the causal data obtained from mice where stress causes cilia gene and subsequent length changes or the conditional KO where stress changes and cilia length changes were observed."

The following changes were made after consultation with the Editors.

We agree that this is an important point. As already shared in the Source Data (files 3, 11 and 12) there is overlap between the human neuropsychiatric datasets and the chronic restraint stress model at the level of differentially expressed genes. This supports the relevance of mouse findings to human disease. We agree, however, that the more informative comparison is at the pathway level rather than the individual gene level, because primary cilium programs may be perturbed through different genes in human and mouse datasets. At this level, the overlap is clear and centered on primary cilium biology (e.g., cilium organization, cilium assembly, protein localization to the primary cilium, axoneme assembly, and others). We mention these comparisons in the Main text on page 9 and in Extended Data Fig. 16v-x.

We also agree that direct causal inference in humans is not possible from gene expression datasets, and we therefore avoid that language in the manuscript – as advised during the last review. Accordingly, we use the term contributory rather than causal throughout – again as previously guided. We will also add a brief statement on page 9 noting that differences between the human and mouse datasets at the level of genes likely reflect methodological and biological factors, including snRNA-seq versus RiboTag RNA-seq, age, species, and redundancy among individual cilium-related genes for common mechanisms.

We do not think a direct comparison between the human neuropsychiatric datasets and the Ift88 conditional knockout data is as informative. The Ift88 manipulation was local to the amygdala and produced anxiety-like behavior, whereas the stress paradigms and human datasets capture broader biological and clinical complexity that cannot be recapitulated by a single local gene deletion. For that reason, the chronic restraint stress model is the most appropriate bridge between the mouse and human data.

“Additionally, to underscore the relevance of the sequencing data that revealed cilia-related gene changes to depression, the authors should include human sequencing data where such changes are not observed for instance in Alzheimer’s Disease or Parkinson’s Disease.”

We do not think such comparisons would be appropriate or informative for this manuscript. First, depression is a prevalent comorbidity in dementia including in PD and AD (see PMIDs 36831861, 21537355, 41943747, 36106206) and the available snRNA-seq datasets for PD and AD do not separate depression as an independent variable. Second, the available AD and PD datasets typically derive from brain regions different from the amygdala, which was the focus of our mouse and human studies and is central to stress- and emotion-related phenotypes. As we show in the manuscript, astrocytes differ across brain regions including in their primary cilium-related genes, so cross-disease comparisons across non-matched regions would be difficult to interpret. Third, we do not want to extend our conclusions beyond the systems we studied experimentally – which follows guidance from the first round of reviews to be more balanced.

That said, we revised the Discussion to note that stress- and depression-related phenotypes are common in neurodegenerative disease, and that future studies will be needed to determine whether primary cilium mechanisms contribute to specific neurological disease contexts. This possibility is already mentioned in the Abstract and now in the Discussion on page 12.

Referee #2 (Remarks to the Author):
“I co-reviewed this manuscript with one of the reviewers who provided the listed reports.”

Thank you

Referee #3 (Remarks to the Author):
"The authors have addressed my comments."

Thank you

Referee #4 (Remarks to the Author):
"The technical considerations raised have been addressed. Conceptually, this manuscript reflects substantial effort and presents one of the first thorough descriptions of astrocyte cilia, yet, a clearly contextualized relationship between astrocytic S1pr1 and cilia remains. This should be acknowledged in the discussion."

We thank the reviewer for recognizing the effort of the study and that the technical considerations have been addressed. We have added the suggested statement acknowledging the need for a better contextualization of the relationship between S1pr1 and primary cilia on page 11.

"Consider reducing the lengthy discussion in CRS, WAS, and SNI induce stress-related behavioral phenotypes. These are established stress-inducing models."

We thank the reviewer for this suggestion and have implemented it.

"To increase clarity, I suggest moving antidepressant experiments to "stress-evoked changes to astrocytic primary cilia".

We thank the reviewer for this suggestion. There are two parts to the antidepressant experiments: one where we evaluate the effects of the antidepressants on behavior (ED Fig. 1c-g), which is presented at the beginning of the manuscript, and one where we evaluate the effect of one of the antidepressants on the primary cilium (ED Fig. 7). We prefer to leave the behavioral antidepressant experiments at the beginning of the manuscript with the rest of the behavioral experiments as we believe this makes the work easier to follow. The current flow is the best way to follow the logic of the study.

Referee #5 (Remarks to the Author):
"I co-reviewed this manuscript with one of the reviewers who provided the listed reports."

Thank you

We thank the Editor and all the reviewers for their careful assessment and helpful suggestions. We hope this response addresses the reviewer's concerns clearly and conservatively, without overstating what the data can support.
