# Supplementary material for: Amygdala astrocyte primary cilium mechanisms contribute to stress behaviours
Source: Nature. 2026 Aug 5;657(8131):455–68. doi: 10.1038/s41586-026-10874-0 (PMC13558064; doi:10.1038/s41586-026-10874-0)
Supplement: Supplementary file 2 [file 41586_2026_10874_MOESM1_ESM.pdf]

nature portfolio

Corresponding author(s): Baljit S. Khakh, Katsukuni Mitsui

Last updated by author(s): Jun 21, 2026

Reporting Summary

Nature Portfolio wishes to improve the reproducibility of the work that we publish. This form provides structure for consistency and transparency in reporting. For further information on Nature Portfolio policies, see our Editorial Policies and the Editorial Policy Checklist.

Statistics

For all statistical analyses, confirm that the following items are present in the figure legend, table legend, main text, or Methods section.

n/a	Confirmed	
☐	☒	The exact sample size (*n*) for each experimental group/condition, given as a discrete number and unit of measurement
☐	☒	A statement on whether measurements were taken from distinct samples or whether the same sample was measured repeatedly
☐	☒	The statistical test(s) used AND whether they are one- or two-sided<br>*Only common tests should be described solely by name; describe more complex techniques in the Methods section.*
☒	☐	A description of all covariates tested
☐	☒	A description of any assumptions or corrections, such as tests of normality and adjustment for multiple comparisons
☐	☒	A full description of the statistical parameters including central tendency (e.g. means) or other basic estimates (e.g. regression coefficient) AND variation (e.g. standard deviation) or associated estimates of uncertainty (e.g. confidence intervals)
☐	☒	For null hypothesis testing, the test statistic (e.g. *F*, *t*, *r*) with confidence intervals, effect sizes, degrees of freedom and *P* value noted<br>*Give P values as exact values whenever suitable.*
☒	☐	For Bayesian analysis, information on the choice of priors and Markov chain Monte Carlo settings
☒	☐	For hierarchical and complex designs, identification of the appropriate level for tests and full reporting of outcomes
☒	☐	Estimates of effect sizes (e.g. Cohen's *d*, Pearson's *r*), indicating how they were calculated

Our web collection on statistics for biologists contains articles on many of the points above.

Software and code

Policy information about availability of computer code

Data collection	Human tissue RNAscope: Standard HALO modules were used for data collection.<br>IHC and RNAscope mouse: Fluorescent images were taken using UPlanXApo 4X (NA 0.16), UPlanXApo 10X (NA 0.40), and UPlanFL N 40X (NA 1.30) objective lens on a confocal laser-scanning microscope (FV3000; Olympus) using Fluoview software (Olympus).<br>RNAseq: Sequencing was performed on NovaSeq 6000 or NovaSeq X Plus yielding at least 80 million reads per sample.<br>Proteomics: The data acquisition was carried out on a Bruker timsTOF HT mass spectrometer using a diaPASEF mode of data acquisition. The dia-PASEF windows spanned a precursor range from 300 to 1200 m/z using the following ion mobility parameters: 1/K0 start: 0.60 Vs cm−2; 1/K0 end: 1.60 Vs cm−2; ramp time: 100 ms; accumulation time: 50 ms. Data acquisition for cilium proteomics experiments was acquired on a Vanquish Neo UHPLC system coupled to an Astral mass spectrometer (Thermo Fisher Scientific, Bremen, Germany). Data-independent acquisition (DIA) was performed on the Orbitrap Astral mass spectrometer operating in positive electrospray ionization mode. MS1 spectra were acquired at 240,000 resolution across an m/z range of 380–980, with a normalized AGC target of 500% and a maximum injection time of 3 ms. DIA employed sequential 4 m/z isolation windows covering a precursor range of 380-980 m/z. MS2 scans were acquired at 80,000 resolution using a normalized HCD collision energy of 25%, a normalized AGC target of 500%, and a maximum injection time of 7 ms.
Data analysis	RNaseq: Demultiplexing was performed with Illumina Bcl2fastq2v.17 program. Reads were aligned to the mouse mm10 reference genome using the STAR spliced read aligner v2.7, and 70-90% of the reads were uniquely mapped. Datasets of the 13 regions of the CNS reported previously64 were combined with the amygdala dataset by batch correction using Removing Unwanted Variation (RUVr). Differential gene expression analysis was performed with the Bioconductor package limmaVoom (v3.60) with the FDR threshold set at <0.05. Astrocyte-enriched genes were defined as those with log2 FC (IP/Input) >1, FDR < 0.05, and FPKM > 1. For analysis of astrocyte region-specific genes, genes were analyzed by comparing IP samples with the average of IP samples from the other 13 regions using limma-voom. The gene ontology enrichment analysis for astrocyte region-specific genes was performed using Enrichr version of GO (https://maayanlab.cloud/Enrichr/). Unwanted variation in the US, CRS, WAS dataset was removed using RUVr. Some data are missing due to unexpected mouse deaths,

and for corticosterone, data are missing due to unsuccessful blood collection. All DEGs were obtained using limma-voom.
Proteomics: The dia-PASEF files were searched with DIA-NN using an in-silico library generated from a Uniprot database containing all mouse proteins. The output files obtained after DIA-NN were subsequently analyzed by the Fragpipe analyst to identify differentially regulated proteins. Statistical analysis of differentially expressed proteins (DEPs) was done using the Bioconductor package limma (v3.60). GO pathway analysis for molecular function and biological function was performed using Enrichr (https://maayanlab.cloud/Enrichr/).
Calcium imaging: peaks were detected using pracma v1.9.9 and AUC was calculated using DescTools v0.99.55.
Behavior: Open field behavior data were collected and analyzed using Anymaze (Stoelting Co., Wooddale, IL, USA). The behavioral data of the mice in the conditioned place temperature aversion test were collected and analyzed by Thermal Place Preference-2 (BIO-T2CT, Bioseb, North Pinellas Park, FL, USA).
Confocal microscopy images (IHC, RNAscope) were processed with ImageJ (v1.53u – 1.54p).
All data plotting and statistical analyses were done in OriginPro 2024.

For manuscripts utilizing custom algorithms or software that are central to the research but not yet described in published literature, software must be made available to editors and reviewers. We strongly encourage code deposition in a community repository (e.g. GitHub). See the Nature Portfolio guidelines for submitting code & software for further information.

Data

Policy information about availability of data

All manuscripts must include a data availability statement. This statement should provide the following information, where applicable:

- Accession codes, unique identifiers, or web links for publicly available datasets
- A description of any restrictions on data availability
- For clinical datasets or third party data, please ensure that the statement adheres to our policy

All new astrocyte RNAseq data generated in this study have been deposited at the Gene Expression Omnibus with accession identifier GSE285150 and are also available in summary format in Source data. Previously published data for the 13 regions of the brain can be found with the accession identifier GSE198024. All new astrocyte proteomic data generated in this study has been deposited at the MassIVE repository and can be accessed using the identifier MSV000096647.

Research involving human participants, their data, or biological material

Policy information about studies with human participants or human data. See also policy information about sex, gender (identity/presentation), and sexual orientation and race, ethnicity and racism.

Reporting on sex and gender	Postmortem brain tissue samples were analyzed. Sex was determined based on next-of-kin interviews and review of medical records; gender identity was not available. The cohort included equal numbers of male and female donors, and sex was included as a covariate in all analyses. Informed consent for brain donation and research use was obtained from the donor's legal next-of-kin, including permission to share de-identified individual-level data.
Reporting on race, ethnicity, or other socially relevant groupings	No categorization based on socially constructed or socially relevant variables was used in these analyses.
Population characteristics	Human brain tissue from four donors (2 females, 2 males; mean age = 62.3 years; all White/Caucasian) was included in these analyses. Toxicology screening was negative for substance use and for medications commonly associated with brain disorders (e.g., antipsychotics, antidepressants, and opioids typically used in palliative care). Serological screening was negative for HIV, hepatitis B, and hepatitis C. Expert review of medical records found no evidence of neurological or psychiatric disorders beyond conditions commonly observed in the general population (e.g., occasional migraine or insomnia). Neuropathological examination did not identify diagnostic abnormalities.
Recruitment	Postmortem brain tissue samples were obtained from the Harvard Brain Tissue Resource Center (HBTRC), a NeuroBioBank site supported by the US National Institutes of Health. The HBTRC employs a community-based model and partners with advocacy groups and family foundations focused on specific brain disorders to promote awareness of brain donation for research. Potential donors may also be referred by third parties, such as medical examiners. Prospective donors may register their intent in advance. At the time of death, a representative notifies the HBTRC. Informed consent for donation is obtained from the donor's legal next-of-kin.
Ethics oversight	The HBTRC study protocol, describing all is procedures, is approved by the Institutional Review Board (IRB) at Mass General Brigham and comply with relevant ethical regulations for research involving human postmortem tissue.

Note that full information on the approval of the study protocol must also be provided in the manuscript.

Field-specific reporting

Please select the one below that is the best fit for your research. If you are not sure, read the appropriate sections before making your selection.

☒ Life sciences ☐ Behavioural & social sciences ☐ Ecological, evolutionary & environmental sciences

For a reference copy of the document with all sections, see nature.com/documents/nr-reporting-summary-flat.pdf

Life sciences study design

All studies must disclose on these points even when the disclosure is negative.

Sample size	Sample sizes were based on previous experiments using similar or identical experiments and from the use of similar models by our laboratory. Refs: Srinivasan et al., 2016; Chai et al., 2017, Nagai et al., 2019; Yu et al., 2020; Diaz-Castro et al., 2019; Gangwani et al., 2023; Soto et al Nature 2023; Ollivier et al Nature 2024; Endo et al Science 2022
Data exclusions	No data were excluded from this manuscript unless there were mitigating issues (which was rare as described here). Standard pre-established exclusion criteria relating to the health of the mice were applied: surgical complications (infection, bleeding, failure to recover from anesthesia), clinical signs requiring immediate euthanasia (low body temperature, persistent sedation, marked decreased resistance to handling, seizures, unresponsiveness), drug-related adverse reactions (respiratory depression, severe tremor, convulsion). Thus, for the behavior work, 300 mice were used over nearly 4 years and from these 5 were removed from the dataset because they were moribund and/or died. Three mice did not perform the behaviors and were thus outliers within the assessments and five mice had to be removed from the corticosterone measurements because we failed to measure plasma corticosterone due to technical reasons related to the volume of available blood. In the cilium floxed behavior experiments, one of the measurements of SPT for one mouse was not written down in the notebook and therefore was not included in the report. No mice or data were removed from any of the molecular or cellular studies.
Replication	To verify the reproducibility of the experimental findings, all data collection was done in multiple batches comprising at least three replicates, including RNAseq samples, proteomics samples, behavioral and calcium imaging experiments. All attempts at replication were succesful.
Randomization	For all experiments, mice were randomly allocated in alternation to a group as they became available and of age from the breeding colony or from the vendor.
Blinding	Because of the experimental design and availability of resources (the project spanned over 4 years and involved several experimenters) blinding was not feasible. For behavioral experiments, we minimized subjectivity as much as possible by using automated analysis software whenever possible.

Reporting for specific materials, systems and methods

We require information from authors about some types of materials, experimental systems and methods used in many studies. Here, indicate whether each material, system or method listed is relevant to your study. If you are not sure if a list item applies to your research, read the appropriate section before selecting a response.

Materials & experimental systems

n/a	Involved in the study	
☐	☒	Antibodies
☒	☐	Eukaryotic cell lines
☒	☐	Palaeontology and archaeology
☐	☒	Animals and other organisms
☒	☐	Clinical data
☒	☐	Dual use research of concern
☒	☐	Plants

Methods

n/a	Involved in the study	
☒	☐	ChIP-seq
☒	☐	Flow cytometry
☒	☐	MRI-based neuroimaging

Antibodies

Antibodies used

guinea pig anti-NeuN (1:1000; Synaptic Systems, 266004)
rabbit anti-cFos (1:1000; Synaptic Systems, 226008)
chicken anti-mCherry (1:1,000; Abcam, ab205402)
chicken anti-GFAP (1:1000; Abcam, ab4674)
rabbit anti-S100β (1:1000; Abcam, ab41548)
guinea pig anti-S100β (1:500; Nittobo, MSFR105350)
chicken anti-S100β (1:500, Synaptic Systems, 287006)
rabbit anti-HA tag (1:1000; Abcam, ab9110)
rabbit anti-Septin2 (1:100; Thermo Scientific, 11397-1-AP)
rabbit anti-Arl13b (1:500, Proteintech, 17711-1-AP)
rat anti-Arl13b (1:500, BiCell Scientific, 90413)
rabbit anti-S1pr1 (1:50, Invitrogen, #PA1-1040)
rabbit anti-AC3 (1:4000, Invitrogen, PA5-35382)
rabbit anti-Sox9 (1:500; Millipore, AB5535)
goat anti-collagen IV (1:500; Novus Bio, NBP1-26549)
biotinylated WFA (1:1,000; Vector labs, B-1355-2)
mouse anti-HA antibody (~1:160, Biolegend, 901514)

Alexa Fluor 488 goat anti-guinea pig (A11073)
Alexa Fluor 546 goat anti-chicken (A11040)
Alexa Fluor 647 goat anti-rabbit (A21244)

	streptavidin Alexa Fluor 647 conjugate (S21374)<br>Opal 520 fluorophore (Akoya Biosciences, FP1487001KT)<br>Opal 570 fluorophore (Akoya Biosciences, FP1488001KT)
Validation	Most of the antibodies used in this manuscript have been validated and reproduced by our lab across at least 9 manuscripts by checking cell specificity, background signal, and noting antigen specificity using western blot techniques (Srinivasan et al., 2016; Chai et al., 2017, Nagai et al., 2019; Yu et al., 2020; Diaz-Castro et al., 2019; Gangwani et al., 2023; Soto et al Nature 2023; Ollivier et al Nature 2024; Endo et al Science 2022, Wu et al 2025).<br>The Arl13b antibody (Proteintech, 17711-1-AP) was validated by the supplier by KO/KD and by publications listed on the website.<br>The Arl13b antibody (Proteintech, 17711-1-AP) was validated by the supplier for immunofluorescence of rodent tissue.<br>The AC3 antibody was validated by the supplier and by publications listed on the website.<br>The Septin2 antibody was validated by the supplier and by publications listed on the website.<br>The S1pr1 antibody was validated by the supplier and by publications listed on the website.<br>The collagen IV antibody was validated by the supplier by biological strategies and by publications listed on the website.

Animals and other research organisms

Policy information about studies involving animals; ARRIVE guidelines recommended for reporting animal research, and Sex and Gender in Research

Laboratory animals	Wild-type C57BL/6NTac mice from The Jackson Laboratory or Taconic Biosciences. RiboTag mice (B6N.129-Rpl22tm1.1Psam/J, JAX Stock # 011029) were acquired from JAX and crossed with Aldh1l1-Cre/ERT2 BAC mice (B6N.FVB-Tg(Aldh1l1-Cre/ERT2)1Khakh/J, JAX Stock #029655). Arl13blox/lox x Aldh1l1-creERT2 (Arl13bcKO). GCaMP6f mice (Ai95D (C57BL/6J), JAX stock #028865) mice were acquired from JAX and crossed with Aldh1l1-Cre/ERT2 BAC mice (B6N.FVB-Tg(Aldh1l1-Cre/ERT2)1Khakh/J, JAX Stock #029655) in an in-house breeding colony. Arl13blox/lox and Ift88lox/lox mice were a kind gift from Dr. J. Guo and were maintained in an in-house breeding colony at UCLA. All mice were 2-5 months old when used for experiments, were healthy with no obvious behavioral phenotype, were not involved in previous studies and were euthanized during the light cycle.
Wild animals	The study did not involve wild animals.
Reporting on sex	All experiments contained male and female mice in approximately equal amounts. Sex-specific differences were not of specific interest to our study and were not independently evaluated. No sex differences were observed in the results.
Field-collected samples	This study did not use field-collected samples.
Ethics oversight	All experiments were conducted in accordance with the National Institutes of Health (NIH) Guide for the Care and Use of Laboratory Animals or the Canadian Council of Animal Care guidelines, and were approved and overseen by the Chancellor's Animal Research Committee (ARC) at the University of California, Los Angeles (UCLA), the Institutional Animal Care and Use Committee at ONO Pharmaceutical Co., Ltd., or the University of Calgary Health Sciences Animal Care Committee.

Note that full information on the approval of the study protocol must also be provided in the manuscript.

Plants

Seed stocks	NA
Novel plant genotypes	NA
Authentication	NA
